# How to get your goat: automated identification of species from MALDI-ToF spectra

**DOI:** 10.1093/bioinformatics/btaa181

**Published:** 2020-03-16

**Authors:** Simon Hickinbotham, Sarah Fiddyment, Timothy L Stinson, Matthew J Collins

**Affiliations:** b1 BioArch, University of York, York YO10 5DD, UK; b2 McDonald Institute for Archaeological Research, University of Cambridge, Cambridge, CB2 3ER, UK; b3 North Carolina State University, Raleigh, NC 27695, USA; b4 Evogenomics Section, University of Copenhagen, Copenhagen 1307 K, Denmark

## Abstract

**Motivation:**

Classification of archaeological animal samples is commonly achieved via manual examination of matrix-assisted laser desorption/ionization time-of-flight (MALDI-ToF) spectra. This is a time-consuming process which requires significant training and which does not produce a measure of confidence in the classification. We present a new, automated method for arriving at a classification of a MALDI-ToF sample, provided the collagen sequences for each candidate species are available. The approach derives a set of peptide masses from the sequence data for comparison with the sample data, which is carried out by cross-correlation. A novel way of combining evidence from multiple marker peptides is used to interpret the raw alignments and arrive at a classification with an associated confidence measure.

**Results:**

To illustrate the efficacy of the approach, we tested the new method with a previously published classification of parchment folia from a copy of the Gospel of Luke, produced around 1120 C.E. by scribes at St Augustine’s Abbey in Canterbury, UK. In total, 80 of the 81 samples were given identical classifications by both methods. In addition, the new method gives a quantifiable level of confidence in each classification.

**Availability and implementation:**

The software can be found at https://github.com/bioarch-sjh/bacollite, and can be installed in R using devtools.

**Supplementary information:**

Supplementary data are available at *Bioinformatics* online.

## 1 Introduction

Matrix-assisted laser desorption/ionization time-of-flight (MALDI-ToF) mass spectrometry (MS) is an established tool for discriminating between species from bone fragments in the absence of morphological markers (Buckley *et al.*, 2009), and has many applications in food safety (Di Francesco *et al.*, 2018), forensics (Francese, 2019) and archaeology (Kuckova *et al.*, 2009). Samples of collagen-based artefacts analyzed in this way form MS with distinctive ‘marker’ peaks which form the base data of the identification process, since collagen peptides form a ‘barcode’ of peaks in the MS. Although the principle of assigning peaks to collagen peptides and thus deducing the species is straightforward, several issues must be overcome in order to arrive at a convincing identification. Post-translational modifications of the collagen peptide sequence can shift the expected position of peaks in the spectra as a result of the change in mass. The method of preparation of the sample appears to have an effect on which peptides ‘fly’ in the MALDI-ToF—a much smaller proportion of peaks are observed than would be expected if all of the peptides produced by cutting collagen with perfectly functioning porcine trypsin produced peaks. The MALDI-ToF apparatus requires a degree of calibration, meaning that the observed mass of a peak can be a weak approximation of the theoretical value. Any strategy for successfully tracing the observed peaks in an MS back to a peptide that appears in a species’ collagen sequence must take these issues into account.

For these reasons, direct use of sequence data for species identification using MS has until now been intractable, and a degree of expert judgement and interpretation of other evidence associated with a MS have been needed to arrive at an identification. However, there is a significant time cost in carrying out the manual analysis and a bottleneck in the availability of trained experts. In addition, the resulting identification is merely qualitative, so the degree of confidence in the identification and the merits of possible alternative classifications cannot be stated numerically.

Here, we describe a new software-based approach to the identification of species from MS. With advances in proteomic analysis and increasing availability of sequence data, it has become possible to predict the position of peaks in MS based on the composition of peptides in a sample and then to align the observed peaks with the predicted peak positions. The distribution of isotopes of peptides can also be predicted, along with the effects on the mass of any post-translational modifications. Once predicted masses are available, peaks in the MS can be aligned to the predicted distribution using the well-established technique of cross-correlation. This allows a peak to be either accepted as being caused by the peptide under consideration, or rejected and removed from subsequent analysis. The software was developed in the open-source programming language R, and is available for download as an R package from https://github.com/bioarch-sjh/bacollite.

The methodology we present here is primarily aimed at collagen-based archaeological samples such as skin, bone and hair, but it can be applied to any MS classification problem where the protein sequence is available. To demonstrate the efficacy of the approach, we compare an automated classification with a classification from a human expert for a typical task for which MS would be used. The dataset in question consists of non-destructive samples from eraser crumbs (Fiddyment *et al.*, 2015) of each bifolio of *Lucas glo. cum A* a 12th-century manuscript from St Augustine’s Abbey Canterbury and held in private hands (Gibbons, 2017). Historical evidence indicates that these folia are made of animal skins from only three species: *Ovis*, *Bos* and *Capra*. We present a comparison of the automated and expert classifications to illustrate the efficacy of the new technique.

## 2 Approach

There are three stages to the new approach. First, we use proteomic data to generate a set of ‘theoretical’ peptide marker masses for each species under consideration. The mass distribution of ions for each peptide marker is calculated, taking into account the possibility of post-translation modifications and incorporating the isotopes of each element in the peptide. Second, the presence and magnitude of peaks at the mass of each theoretical peptide are measured by application of cross-correlation. Finally, the correlation data for the set of markers for each candidate taxon are combined to give a score to the identification, with the classification being based on the highest scoring taxon.

### 2.1 Generating ‘theoretical’ peaks

**Fig. 1.**
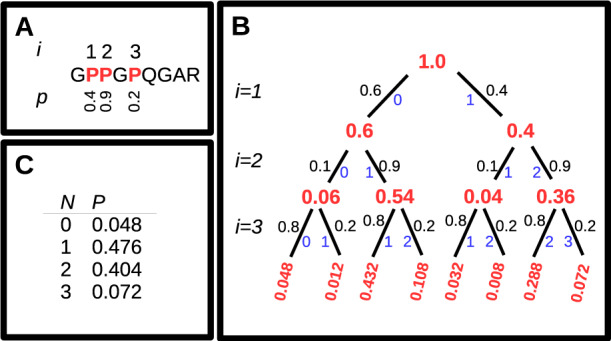
Calculation of the probability of different levels of hydroxylation events in a peptide. (**A**) A peptide with three prolines in its sequence. *i* gives the index of each proline, and *p* shows the hydroxylation probability. These probabilities are provided to illustrate the approach and should not be taken as typical values for the sequence shown. (**B**) How a binary decision tree is used to calculate the probabilities of all possible combinations of hydroxyproline and proline. The calculation goes from the top to bottom of the tree, considering each proline in turn. Numbers in red show the current probability as each proline is considered. Numbers in black show the probability of descending each branch, and numbers in blue show the current number of hydroxylations for each branch. (**C**)The resulting probability for each level of hydroxylation, achieved by summing the probability at the bottom of the decision tree for each level of hydroxylation

In order to carry out the analysis, the collagen sequence(s) of the candidate species must be available. For each candidate species, the following three steps are taken:

1. *Theoretical spectra:* Peptides in MALDI-ToF are generated by digesting the collagen with porcine trypsin, which cuts at arginine (R) and lysine (K; Buckley *et al.*, 2010). It is therefore straightforward to predict the peptides that will be generated by cutting with porcine trypsin by simply splitting the collagen sequence after each instance of R and K in the sequence. This yields a set of candidate peptides whose mass can be calculated.
2. *Missed cleavages:* Porcine trypsin does not always cut at *every* instance of R and K in the collagen molecule, resulting in peptides containing missed cleaves. Our initial evaluations assume that trypsin cuts at all instances of these amino acids, and that the cut occurs precisely between the K or R and the next amino acid downstream. Once we have aligned observed MALDI-ToF peaks with the set of perfectly cleaved peptides, we can then examine which of the remaining peaks can be explained by peptides with missed cleavages.
3. *Post-translation modifications:* Given the raw peptide sequence, it is also necessary to incorporate the common post-translation modifications that are observed in collagen. These are the hydroxylation of proline (P) to hydroxyproline and the deamidation of glutamine (Q) to yield glutamic acid (E). The primary sequence of collagen is highly ordered, with a repeating ‘GlyXaaYaa’ motif, where the ‘Gly’ is glycine, and Xaa and Yaa can be any other amino acid. The probability of hydroxylation of proline is known to be more likely at the Yaa position in this repeating motif (Shoulders and Raines, 2009), and less likely in the Xaa position. Methods for calculating exact probabilities of hydroxylation for a given proline in a collagen sequence are not yet available, but simple estimates based on Xaa–Yaa position can be used to calculate the probabilities of a particular number of hydroxylations, as shown in Figure 1. Deamidation of glutamine can be reasonably assumed to be an ‘all or nothing’ event, with the effect of increasing peptides by 1Da per Glutamine in the sequence.

**Fig. 2.**
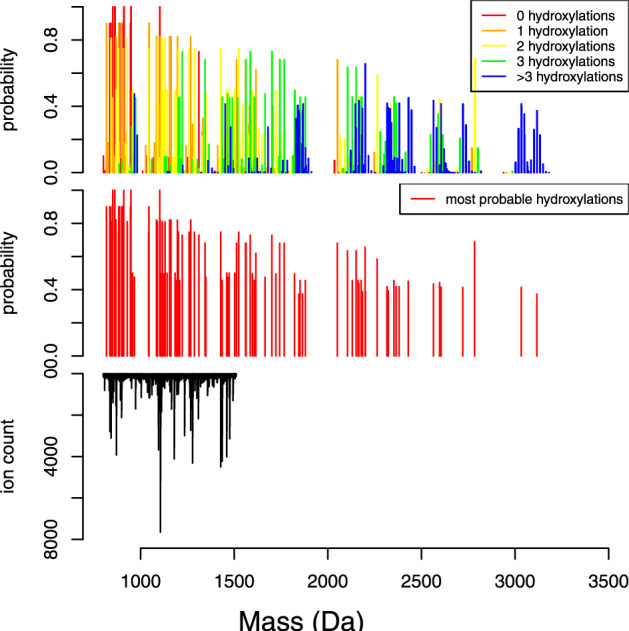
Distribution of theoretical peptides for *Ovis* collagen (top) with the most probable levels of hydroxylation (middle), and compared with ion counts from a MALDI-ToF spectrum of *Ovis* collagen (bottom)

An example of the distribution of theoretical peaks for *Ovis* collagen is shown in Figure 2. The theoretical distribution is plotted above a typical spectrum for *Ovis*, shown in black. It can be seen that there are many more peaks in the theoretical spectrum than the observed sample, but that all the peaks in the real data correspond to the peaks with the more probable levels of hydroxylation as predicted by the theory (known contaminants and missed cleavages can be discounted). Precisely which peptide fragments appear in the MALDI-ToF can be verified by liquid chromatography tandem MS analysis, allowing researchers to determine sets of peptide markers for species identification. The ideal marker peptide is one which is unique to the species under consideration, appears in the majority of spectra of the sample type, has a unique mass among the other peptides in the collagen sequence (and any potential contaminants), and has a high ion count. Having determined all possible peptides in a spectrum, it becomes feasible to identify new markers that can be used to increase confidence in classification. This is the subject of this work in classifying species among a wider range of candidates—known as *ZooMS* (Buckley, 2018).

### 2.2 Aligning theoretical peptides with MS peaks

**Fig. 3.**
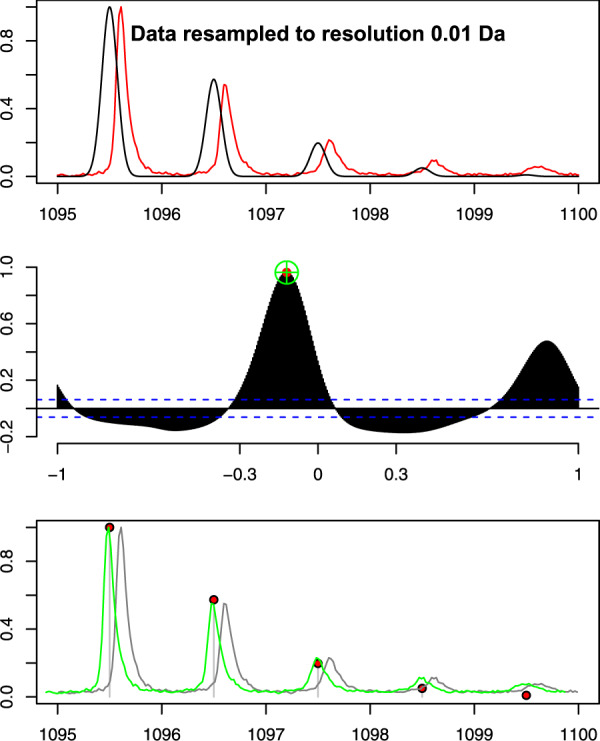
Cross-correlation of peptide ‘GPSGPPGPDGNK’ with a single hydroxyproline at Position 6 with a region of a MALDI-ToF spectrum. Top: MALDI-ToF spectra (in red) and theoretical spectrum for the sequence. Both datasets are resampled every 0.01 Da. Middle: result of the cross-correlation analysis. The highest correlation coefficient is 0.91 at a lag of −0.065 Da. Bottom: alignment of MALDI-ToF peaks with theoretical spectrum achieved by shifting by −0.065 Da. The theoretical peaks are shown as pinheads, the original MALDI-ToF data are shown in grey and the aligned version is shown in green

Having generated a set of theoretical peaks, the next step is to align the observed MALDI-ToF peaks with the calculated masses. As described earlier, observed MALDI-ToF peak masses can be skewed by up to 0.5 Da. Our strategy here is to use cross-correlation to find the best alignment between the calculated isotopic masses of each peptide with a local region of the MALDI-ToF spectra, incorporating the maximum possible drift of 0.5 Da. This process is illustrated in Figure 3. MALDI-ToF data are resampled to regular intervals of 0.01 Da using linear interpolation. The intensity values in the MS data are normalized to the range [0,1]. The theoretical mass distribution of the isotopes is generated by setting the ion distribution values within a zero-valued array of mass–ion pair values with zero ion count. For any peptide, MALDI-ToF peaks do not manifest themselves as single spikes, but tend to form a series of relatively broad peaks due to stochastic effects during the peptide flight. For an individual peptide, peaks for each isotope are spaced at 1 Da apart. The correlation between the theoretical and the observed distribution can be improved by applying Gaussian smoothing to the theoretical distribution of the peptide isotopes as shown in the top row of Figure 3.

The cross-correlation between the local spectrum and the theoretical peak is then calculated. This generates the plot shown in the second row of Figure 3. The alignment is calculated by finding the maximum correlation score within a lag range of ±0.5 Da. As shown in Figure 3, the best correlation is at a lag of −0.065 Da. The local MALDI-ToF data can then be shifted to this position as shown in the bottom row of the figure.

The cross-correlation technique yields two pieces of information that can be used to assess the likelihood that a particular peptide is causing a peak in a MALDI-ToF spectrum: first, the maximum correlation score gives a measure of how well the proposed peptide aligns with the MALDI-ToF peaks; second, the lag shows how much the MALDI-ToF spectra would have to be shifted to obtain the maximum correlation score.

### 2.3 Scoring and classification

Having obtained a correlation score for an individual local alignment for a single peptide, a method is now required to deduce a classification based on these scores. This is not a straightforward task, since the quality of alignments depends on the quality of the peaks which in turn depends upon the entire history of the collagen under consideration—from its formation in a living being, the way it was preserved (often for centuries) through to the way the sample was prepared for analysis. Our experience has shown that it is not feasible to determine a single global threshold on the correlation score below which an aligned match is rejected, since any threshold tends to produce false positives and false negatives due to the high degree of variability of the peaks in the MALDI-ToF. This is certainly a problem in the case of sheep and goat, as they only differ in one high-mass molecular marker (3033 Da in sheep and 3093 Da in goat). The absence of these high-mass peptides would mean that we cannot give a definitive species identification (manually) although the presence of other peaks would be enough to exclude calf. This would be reflected in the automated scoring, as calf would be given a lower score, but sheep and goat would present a similar value. Our analysis is only as good as raw data, and if the collagen preservation is poor the identification will not always be possible.

An alternative approach which we have found to be more robust is to count and combine matches with a correlation score above a *range* of thresholds, and to develop a scoring system based on an analysis of this range. Since a correlation score is always in the range [0,1], it is feasible to use the threshold as a weighting on the number of hits at that threshold level. In the description below, a ‘hit’ is an alignment whose correlation score is higher than a particular threshold.

#### 2.3.1 Classification strategy

Our classification strategy combines evidence for each replicate of a sample when aligned with a set of marker peptides for each target species. The method calculates a score for each candidate species label by considering the number of peptide alignments above correlation thresholds running from 0 to 1 in increments of 0.05. We commence by defining the following variables:

- r∈R is one of the three replicates;
- p∈P is the set of peptides for each taxon *i*;
- *C*_t_ correlation threshold;
- i∈I is the set of taxa from which the identification is drawn;
- $H_{i,t}$ is the number of hits for each peptide/replicate combination for taxon *i* and correlation threshold *C*_t_.

For each replicate *r*, the spectrum is aligned with each peptide *p* for each taxon *i*. An alignment is considered a ‘hit’ and scores 1 if the correlation score $c_{r,p}$ is greater than threshold *C*_t_:

(1) $$H_{i,t}=\sum_{r=1}^{3}\sum_{p\in P_{i}}\left[c_{r,p}>C_{t}\right]$$

The summing equation takes the following form:

(2) $$S_{i}=\sum_{t=0}^{1}\max[0,H_{i,t}-\max(H_{j\neq i,t})]$$

Thus, the measure *S* is high when a particular taxon has more hits than the other taxa under consideration, but relatively low if the sample is more ambiguous. In this way, evidence for an identification of a sample is accumulated using a range of correlation thresholds. The advantages of the measure *S* are that: it yields a confidence score that examines all taxa under consideration; it is high for good samples and low for bad samples; it indicates when more than one possible interpretation is possible; and it is low if the sample is from a species not under consideration. This process allows a complete automated analysis to be carried out once a sample is available.

## 3 Application to a classification problem

**Fig. 4.**
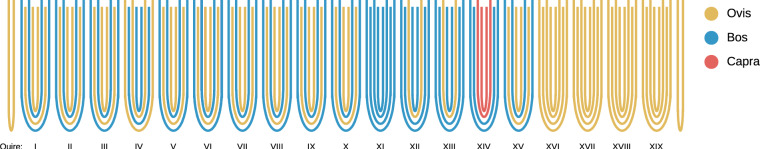
Manual classification of the Glossed Luke Manuscript arranged by quire. Illustration based on the figure in Gibbons (2017, p. 349)

To illustrate how this methodology can be used in practice, we repeat an analysis of the Glossed Luke manuscript data presented in (Gibbons, 2017), where changes in the scribe writing the book corresponds with a change in the species of skin the parchment was made from, as shown in Figure 4. The methods described earlier were used to derive an automated identification of the species for each folio of the manuscript for comparison with the original expert interpretation.

### 3.1 Setting up the automated classifier

We used sequence data for cattle *Bos taurus* and sheep *Ovis aries* referenced in (Buckley, 2016). The Uniprot accesion numbers are: *B.taurus* COL1A1 (P02453); *B.taurus* COL1A2 (P02465); *O.aries* COL1A1 (W5P481); and *O.aries* COL1A2 (W5NTT7). Sequences for goat *Capra aegagrus* were obtained from NCBI accession references NC_030826 REGION: 36105883.36123687 GPC_000002482 for collagen 1A1 and NC_030811 REGION: 108819725.108857001 GPC_000002467 for collagen 1A2. Fasta files for this dataset are included as Supplementary Material.

Parchment is commonly made from sheep, calf and goat skin. During manual classification at least seven peptides are used to identify the sample as being from one of these three species. However there are three crucial peptides that are used to discriminate between the three species and in the case of sheep and goat there is one critical peptide (3033 versus 3093) that differentiates them. The manual identification described in Gibbons (2017) confirmed that each sample came from one of these three species, but to demonstrate how our automated technique handles an outlier candidate species, we have included collagen sequences for European red deer *Cervus elaphus hippelaphus*. Uniprot accession numbers for deer collagen are A0A212D9F7 for COL1A1 and A0A212CJT1 for COL1A2.

When the sequences of these species are cut with porcine trypsin, the majority of peptides are identical for the candidate species. There are 15 different peptides between *Bos* and *Capra*, 14 between *Bos* and *Ovis*, and only 3 between *Ovis* and *Capra*, reflecting the close phylogenetic relationship between these species. Unfortunately, peaks corresponding to the masses of only a small number of these peptides commonly appear in MALDI-ToF spectra. These are shown in Table 1, along with the corresponding peptides for *Cervus*, which also differs from *Ovis* in only one of the peptides used for classification. Note that peptide F1 is the result of a missed cleavage.

**Table 1.** Peptides and masses used to align spectra for classification

Code	Species	Peptide	Mass	*nH*
A1, A2	*Ovis*	TGQPGAVGPAGIR	1180.6	0,1
	*Capra*	TGQPGAVGPAGIR	1180.6	0,1
	*Bos*	IGQPGAVGPAGIR	1192.7	0,1
	*Cervus*	TGQPGAVGPAGIR	1180.6	0,1
F1	*Ovis*	GLTGPIGPPGPAGAPGDKGETGPSGPAGPTGAR	2883.4	2
	*Capra*	GLTGPIGPPGPAGAPGDKGETGPSGPAGPTGAR	2883.4	2
	*Bos*	GLTGPIGPPGPAGAPGDKGEAGPSGPAGPTGAR	2853.4	2
	*Cervus*	GITGPIGPPGPAGAPGDKGETGPSGPAGPTGAR	2883.4	2
G1, G2	*Ovis*	GPSGEPGTAGPPGTPGPQGLLGAPGFLGLPGSR	3017.5	4,5
	*Capra*	GPSGEPGTAGPPGTPGPQGFLGPPGFLGLPGSR	3077.5	4,5
	*Bos*	GPSGEPGTAGPPGTPGPQGLLGAPGFLGLPGSR	3017.5	4,5
	*Cervus*	GPSGEPGTAGPPGTPGPQGIIGPPGFIGIPGSR	3059.5	4,5

*Note*: Differences in sequences compared with that for *Ovis* for each peptide code are highlighted in red. Where two hydroxylation levels are reported, the mass for the lower level is shown.

In order to compare the technique directly with the methods used in Gibbons (2017), we aligned samples with the peptides from each species that corresponded with their respective peaks. We used the same MALDI-ToF spectra as the original study to eliminate differences in classification that could arise via different techniques for generating them. Samples were generated by extracting degraded collagen which was gelatinized prior to enzyme treatment with porcine trypsin. Samples spotted in triplicate were analyzed using a calibrated Ultraflex III (NLD1; Bruker Daltonics) MALDI-ToF instrument in reflector mode. Spectral analysis was performed using the open-source cross-platform software mMass (www.mmass.org; Strohalm *et al.*, 2010).

### 3.2 Classifying folia in the Glossed Luke manuscript

**Fig. 5.**
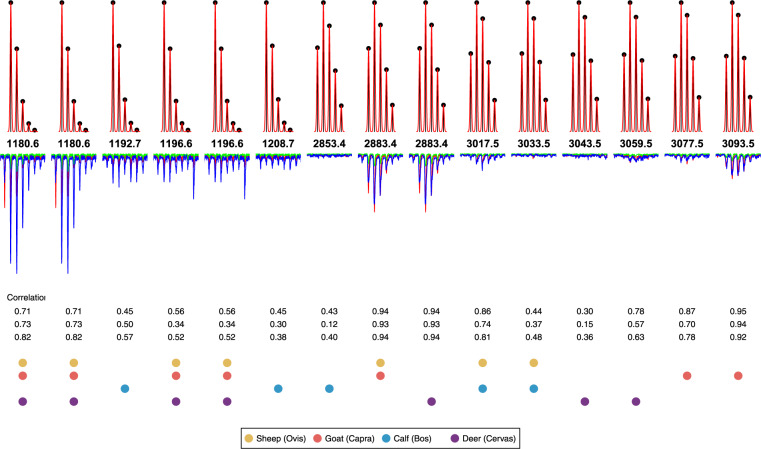
Aligning the reference peaks with sample BZ15 from the Glossed Luke dataset. Top row: reference peaks where mass and intensity for each isotope is shown in black with the result of Gaussian smoothing shown in Red. Second row: mass values of the first theoretical peak of each peptide in Daltons. Third row: sample BZ15 replicates shown in red, green and blue, with inverted y axis to allow comparison with the theoretical peaks. Rows 4–6 show the correlation score for each replicate. Rows 7–10 indicate with a dot whether a peptide is present in sheep (red), goat (green) calf (blue) or deer(black) collagen (see Table 1 for further details)

The automated classification was carried out as follows. The dataset contained three replicates of samples from each folio. Each of these replicates was aligned with each of the peptides shown in Table 1. The maximum permitted lag on the alignment was ±0.5 Da. The alignment process is illustrated for sample BZ15 in Figure 5, which was manually identified as *Capra*. It can be seen from the correlation values score for each of the marker peptides aligned with this sample is higher for *Capra* than for *Ovis*, *Bos* or *Cervus*. The scoring system described in Section 2 was then used to derive a classification of the sample to *Ovis*, *Capra*, *Bos* or *Cervus* from the correlation scores for each sample/peptide combination.

**Fig. 6.**
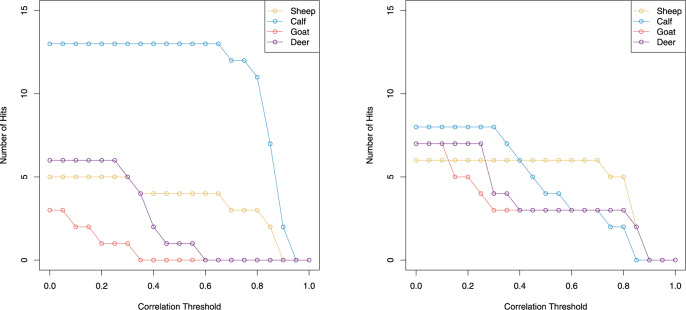
Classification of two samples from the Glossed Luke manuscript. The horizontal axis shows the threshold above which correlations are scored as a ‘hit’. The vertical axis shows the count of hits $H_{i,t}$ (from Equation 1) for each replicate. In this analysis, the maximum number of hits could be fifteen. As the correlation threshold increases, the number of accepted hits decreases. The plot on the left shows a confident ID of *Bos*, whereas the plot on the right shows a more ambiguous classification of *Ovis*

One of the goals of developing an automated classification system is to give a numeric value to the confidence of a classification. This is particularly challenging in MALDI-ToF spectra, where some of the peaks that allow discrimination have a low signal-to-noise ratio. Figure 6 shows how the scoring scheme is used to give a confidence estimate to classification as each of the candidate species. The left-hand panel shows the number of acceptable alignments for the range of different correlation thresholds. Since there are five peptides and three replicates, the maximum possible number of matches is 15. The data for this panel come from folio 28 recto and are classified as *Bos*. It can be seen that for this species, 13 of the 15 alignments are matches up to a correlation threshold of 0.65. The other two species never have more than five matches, and never have more matches than *Bos* no matter what the threshold. Thus, the only non-zero scoring ID is for *Bos*. In contrast, the right-hand panel shows a more ambiguous classification of folio 36 recto. Here, the score for *Ovis* is relatively lower, and the *Bos* classification score is non-zero also. This is because there are more matches for *Bos* at lower correlation thresholds than *Ovis*, but as the threshold increases, the matches for *Ovis* are still higher than the threshold.

### 3.3 Results

When compared with expert interpretation of the spectra from the Glossed Luke manuscript, the automated system agreed with the expert’s own for 80 of the 81 folio samples. Within this classification, three samples from folios 109 to 111 were identified as *Capra* by both methods. 44 *Ovis* samples were manually identified, of which one of these was misclassified as *Bos* by the automated method. The remaining 34 samples were identified as *Bos* by both methods. None of the samples were misclassified as our control species, *Cervus*.

The three replicates for the misclassified sample did not show the peaks used for discrimination consistently. One replicate was of poor quality and depending on which of the remaining two replicates was examined, peaks at 2853 Da (calf) and 2883 (sheep/goat) could be identified. The final identification as sheep was originally achieved by expert visual inspection of the folio in question (and to the general construction of the quires). This misclassification emphasizes the strength of the approach, since both *Bos* and *Ovis* had low scores of 6.25 and 1.70, respectively, compared with scores of over 50 for unambiguous samples.

### 3.4 Getting the goat—context and interpretation

Medieval manuscript books were constructed from bifolia, i.e. parchment sheets folded in half, that were nested inside of one another to form quires; the finished quires were stacked together and sewn into a binding. As shown in Figure 4, the automated classification we present above mirrors the manual classification almost completely, revealing that there are only two goatskin bifolia in the Glossed Luke manuscript, forming four leaves (or 8 pages) in the middle of the 14 quire. The use of such a small amount of goatskin is intriguing. The main Gospel text was completed by two scribes, with the commentary added later in the margins by several other hands. Scribe 1 completed the first 14 quires and began the 15th before the work was taken over by a second scribe. The first 10 quires share the same organization of alternating calf and sheep (although the order is reversed in Quire IV). Quires XI–XIV, however, are dominated by calfskin, with only two bifolia each of sheep and goat. Scribe 1 began the first recto (i.e. obverse) of Quire XV, after which a second scribe completed the remaining Gospel text. All subsequent quires assembled for Scribe 2 are composed entirely of sheep.

It is possible that *Capra* was the only skin available to the scribe when Quire XIV was being completed. There is however another intriguing alternative: Quire XIV comprises two outer bifolia on calfskin with two inner bifolia on goat. The text at the top of the first leaf on goatskin is midway through Luke 16:10. This means that just a few leaves earlier—written on the calfskin portion—we find the sole mention of goats in the Gospel of Luke. In verse 15:29, the famous parable of the prodigal son, the older brother complains that his father had never given him even a single young goat for celebration, while the wayward brother warrants the fattened calf:

at ille respondens dixit patri suo ecce tot annis servio tibi et numquam mandatum tuum praeterii et numquam dedisti mihi hedum ut cum amicis meis epularer (Vulgate Bible)

And he answering, said to his father: Behold, for so many years do I serve thee and I have never transgressed thy commandment: and yet thou hast never given me a kid to make merry with my friends. (Douay-Rheims Bible)

Since this is the first complete book analyzed in this manner, the evidence at this early stage only allows us to speculate. Yet, the clear possibility remains that the placement of the sole goatskin in the manuscript—immediately following the sole mention of goats in the Gospel—might be intentional rather than merely a product of which skins were available; these findings also suggest that in future studies researchers should be aware of the possibility that hides might be intentionally placed in a manner that reflects the text. These observations challenge the assumption that the goatskin was used as a last resort, and provide a potential alternative interpretation that sheds new light on the construction of manuscripts in the 12th century.

## 4 Discussion

We have presented an automated method for the classification of MALDI-ToF spectra at the species level between three closely related mammals. Automated techniques for the analysis of MALDI-ToF spectra gives consistency to the classification task, and allows researchers to describe the route to a particular classification. These methods must accommodate a high degree of variability in the way peptides manifest themselves (if at all) as peaks in MALDI-ToF spectra. Expert manual analysis generally only considers peaks to be present if they have a signal-to-noise ratio of 3. The correlation-based approach allows all candidate peaks to be compared with the simulated peaks derived from genetic data, and is capable of distinguishing signal from noise without thresholding. More degraded samples will give a lower identification score, allowing the confidence of the interpretation to be included with other considerations about the species identity of the artefact under study. For example, this method seems to have the same level of efficiency as a manual interpretation as can be seen in the case of BZ34. If samples are particularly degraded they would not be able to be identified even with an experienced analyst; therefore, we assume the level of efficiency of this software will be very similar to a manual analysis.

Although ‘black box’ AI techniques such as deep learning can yield convincing classifications, the means by which the classification is arrived can be hard to resolve due to the manner in which the weights in the neural network are used to produce it. This lack of transparency in the reasoning that has been used to derive a classification becomes problematic when trying to resolve species identification where other potentially conflicting information is available, especially in the case where training data has been labelled only via expert opinion. In contrast, the method presented here has a clear methodology based on statistical analysis of the shape of peaks with respect to the peptides which caused them.

In the example study, it is remarkable how the peptide data tally with the manual analysis—both in the identification of the discriminatory peptide markers and the final assignment of taxon to sample. We plan to extend this approach to classification tasks with a higher number of candidate taxa and with a greater number of marker peptides.

## Funding

This work was supported by the Marie Curie International Fellowship [PALIMPSEST FP7-PEOPLE-2011-IEF 299101], the British Academy Postdoctoral Fellowship funding, Danish National Research Foundation [DNRF128] and the ERC Investigator [295729-CodeX and 787282-Beasts to Craft].

*Conflict of Interest*: none declared.

## Supplementary Material

btaa181_Supplementary_Data
