# Supplementary material for: How to get your goat: automated identification of species from MALDI-ToF spectra
Source: Bioinformatics. 2020 Mar 16;36(12):3719–25. doi: 10.1093/bioinformatics/btaa181 (PMC7320604; doi:10.1093/bioinformatics/btaa181)
Supplement: btaa181_Supplementary_Data [file btaa181_supplementary_data.zip › btaa181-Suppl_Data/Species_ID_Using_Bacollite.pdf]

Aligning Sample To Peptides for sheep,cow,goat and deer

Simon Hickinbotham

1 Introduction

This tutorial will demonstrate how to load three replicates of a sample from text files and align them with some peptides from four different species using the `bacollite` R package.The basic process of aligning peaks to peptides will be described first, and then details regarding the process of classifying the spectra based on peptide information will be given. To summarise, the process involves the following steps:

- Load a MALDI dataset
- Load theoretical peptides for several species
- Align the MALDI data with the peptides
- Give a score to the peptides from each species, to deduce the species ID
- Generate graphical representation of the scoring

This document is provides as supplementary information to the paper "How to Get Your Goat: Automated Identification of Species from MALDI-ToF Spectra". The experiments reported in that paper were carried out in R in the same way as shown below, using the `bacollite` package developed by the authors. For more information, please browse vignettes for this package, which can be downloaded from `https:\\github.com\bioarch-sjh\bacollite`.

To run the code in this document, the bacollite package must be installed, and this in turn requires the devtools package.

```
install.packages("devtools")
devtools::install_github("bioarch-sjh/bacollite",force=T)
```

Once installed, the package can be loaded in the usual way

```
require(bacollite)
```

2 How to load data

2.1 Setting the data directory

We can load data from a range of different text files using bacollite, which was designet to be very flexible in terms of the filename and the delimiter between columns of data. For loading raw data in Bruker format, see the package `MALDIquantForeign` on CRAN.

The bacollite package provides some example data files that can be used during this tutorial. We need some R code to find where they are on our system. To get R to tell us the folder that the data is in we use the following command:

```
fpath <- system.file("extdata",package="bacollite")
```

The variable `fpath` now holds the path to the sample files that come with bacollite. We'll be using the `fpath` variable in what follows. With your own data, you would know where the samples are on your file system and you'd create `fpath` using something like:

```
fpath <- "c:\mydata\"
```

2.2 Loading data files for three replicates

The MALDI data files that we are using are called:

`20150326_SF_YG65_94_I11.txt`
`20150326_SF_YG65_94_I14.txt`
`20150326_SF_YG65_94_I17.txt`

You can see that the last part of the file name before the "`.txt`" is the spot reference, and there are three spots: `I11`, `I14` and `I17`. We need this information to use the `load.sample` function to load the three samples into R, like this:

```
library(bacollite)
froot = sprintf("%s/20150326_SF_YG65_94_",fpath)
sample <- load.sample(froot = froot,spots = c("I11","I14","I17"),name="folio 42")
```

Let's go through this line by line:

- `library(bacollite)` loads the package.
- `froot = sprintf("%s/20150326_SF_YG65_94_",fpath)` creates the text that is common to the file path of all three samples, based on the `fpath` text string that we created above.
- `sample <- load.sample(froot = froot,spots = c("I11","I14","I17"),name="folio 42")` loads the data from the files into an R data object called `sample`.

Let's have a look at the structure of the `sample` object using R's `str` function - this will show you what the arguments mean:

```
str(sample)
#> List of 5
#>  $ name: chr "folio 42"
#>  $ spot: chr [1:3] "I11" "I14" "I17"
#>  $ s1  :'data.frame':    126186 obs. of  2 variables:
#>   ..$ mass     : num [1:126186] 500 500 500 500 500 ...
#>   ..$ intensity: int [1:126186] 7 8 15 10 11 30 26 38 8 8 ...
#>  $ s2  :'data.frame':    126186 obs. of  2 variables:
#>   ..$ mass     : num [1:126186] 500 500 500 500 500 ...
#>   ..$ intensity: int [1:126186] 9 4 6 8 0 4 15 26 8 16 ...
#>  $ s3  :'data.frame':    126186 obs. of  2 variables:
#>   ..$ mass     : num [1:126186] 500 500 500 500 500 ...
#>   ..$ intensity: int [1:126186] 2 0 25 4 11 10 11 27 13 4 ...
```

This tells us that `sample` is a list of five pieces of data:

- `name` - the name of the data. We called it "folio 42". Having names is useful when you are running lots of samples!
- `spot` - the three spot codes for the three samples
- `s1` - the sample data for the first file (`I11`)
- `s2` - the sample data for the second file (`I14`)
- `s3` - the sample data for the third file (`I17`)

Each of the three sample data entries in the list is a data frame containing the MALDI spectrum data that we'll use for the analysis.

3 How to load peptides

There are several sets of peptide sequences that come with `bacollite`. The package comes with some sets of peptides that we can use to descriminate between samples from sheep, cow and goat, as described in the paper [1]. The peptides in `dm_sheep` are:

	seq	nhyd	mass1
2516	TGQPGAVGPAGIR	0	1180.636
2518	TGQPGAVGPAGIR	1	1196.631
804	GLTGPIGPPGPAGAPGDKGETGPSGPAGPTGAR	2	2883.416
2207	GPSGEPGTAGPPGTPGPQGLLGAPGFLGLPGSR	4	3017.490
2209	GPSGEPGTAGPPGTPGPQGLLGAPGFLGLPGSR	5	3033.485

Those in `dm_cow` are:

	seq	nhyd	mass1
900	IGQPGAVGPAGIR	0	1192.673
902	IGQPGAVGPAGIR	1	1208.668
282	GLTGPIGPPGPAGAPGDKGEAGPSGPAGPTGAR	2	2853.406
796	GPSGEPGTAGPPGTPGPQGLLGAPGFLGLPGSR	4	3017.490
798	GPSGEPGTAGPPGTPGPQGLLGAPGFLGLPGSR	5	3033.485

Those in `dm_goat` are:

	seq	nhyd	mass1
904	TGQPGAVGPAGIR	0	1180.636
906	TGQPGAVGPAGIR	1	1196.631
282	GLTGPIGPPGPAGAPGDKGETGPSGPAGPTGAR	2	2883.416
798	GPSGEPGTAGPPGTPGPQGFLGPPGFLGLPGSR	4	3077.490
800	GPSGEPGTAGPPGTPGPQGFLGPPGFLGLPGSR	5	3093.485

Finally, those in `dm_deer` are:

	seq	nhyd	mass1
1781	TGQPGAVGPAGIR	0	1180.636
1783	TGQPGAVGPAGIR	1	1196.631
677	GITGPIGPPGPAGAPGDKGETGPSGPAGPTGAR	2	2883.416
1488	GPSGEPGTAGPPGTPGPQGIIGPPGFIGIPGSR	4	3043.505
1490	GPSGEPGTAGPPGTPGPQGIIGPPGFIGIPGSR	5	3059.500

Details of how to generate peptide sets from raw amino acid sequences are detailed in a separate vignette.

4 Run the alignment

OK, now we've got a sample and some peptides, we can do alignments, using the ms_fit function. Let's do this for each set of markers. If the variable `doplot` is set to `TRUE` then a graphical output will be produced

for each peptide:

```r
par(mfrow=c(4,5))
gauss <- 0.2
sheep_fit <- ms_fit(peptides = dm_sheep, sample = sample,doplot = T,force=T,gauss = 0.2)
cow_fit   <- ms_fit(peptides = dm_cow,   sample = sample,doplot = T,force=T,gauss = 0.2)
goat_fit  <- ms_fit(peptides = dm_goat,  sample = sample,doplot = T,force=T,gauss = 0.2)
deer_fit  <- ms_fit(peptides = dm_deer,  sample = sample,doplot = T,force=T,gauss = 0.2)
```

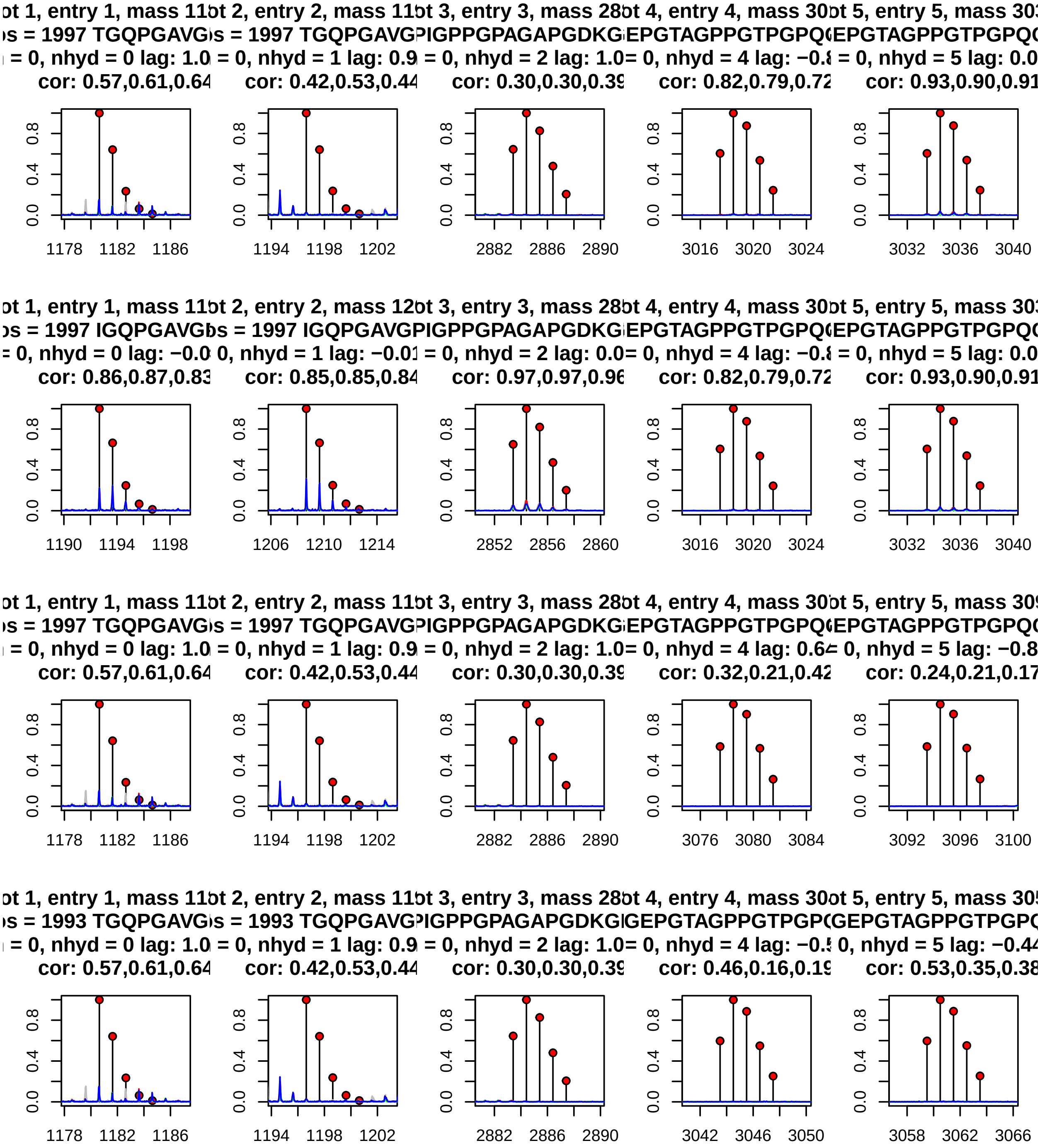

The information in these plots is as follows:

- The x axis shows the mass range under consideration
- The y axis is a scale from 0 to 1
- Each sample is plotted with a coloured line *and* a grey line. Aligned samples 1,2 and 3 are coloured red, green and blue respectively. There original (unshifted) mass positions are also indicated with a grey line. This is useful for *bad* alignments, because it becomes clear that
- The intensity of each sample is scaled by the highest peak in the same spectrum. This allows for a better comparison across many peptides, becuase you see clearly where peaks are small.
- The isotopic distribution of the target peptides is shown as a seris of five "pinheads". These are scaled by the relative value of the most common isotope. For lower masses, this is usually the first isotope but for higher masses it is usually the second.
- As much information as possible is placed in the title of each plot, but this can be overkill if you want to see mmultiple plots in one figure as shown above.

At this point, we have a set of correlation data for each replicate sample with the peptides for each species. The next step is to use this data to carry out a classification of the sample.

5 Perform a classification

We carry out a classification by passing the data objects we created with `ms_fit` into the classfier function `cor_id`. Let's write this as if it was a function first, then call it - that'll help us explain the process.

First we need to create a list of the alignments, and a vector of names for the species ID like this:

```
cordata <- list()
cordata[[1]]<-sheep_fit
cordata[[2]]<-cow_fit
cordata[[3]]<-goat_fit
cordata[[4]]<-deer_fit
corlab <- c("Sheep","Cow","Goat","Deer")
```

The reason we put this data into a list is because it makes it easier to process each species in turn. If we have a list of peptides by species we can use a loop to iterate through every sample.

The first stage in the classification is to determine whether a correlation between a sample and a peptide is a 'hit'. Rather than use a single threshold on the correlation, we use a range of thresholds (stored in the variable `corlim` below) and see if each alignment is above each value in that range. The code below organises this process:

```
# Set up
corlim = seq(0,1,0.05)
scores <- vector(length = length(cordata))
scores[] <- 0
laglim <- 0.6

# Massage the raw cordata into a form we can work with:
cld <- list()
for(cc in 1:length(cordata)){
  cld[[cc]] <- corlim_data(cordata[[cc]],laglim)
}
```

We can look at the structure of the `cld` object, which gives us an idea of what the code above achieves:

```
str(cld)
#> List of 4
#>  $ :'data.frame':    21 obs. of  3 variables:
#>   ..$ cl: num [1:21] 0 0.05 0.1 0.15 0.2 0.25 0.3 0.35 0.4 0.45 ...
```

```
#>   ..$ nh: int [1:21] 5 5 5 5 5 5 5 5 5 5 ...
#>   ..$ sc: num [1:21] 0.00586 0.00586 0.00586 0.00586 0.00586 ...
#>  $ :'data.frame':    21 obs. of  3 variables:
#>   ..$ cl: num [1:21] 0 0.05 0.1 0.15 0.2 0.25 0.3 0.35 0.4 0.45 ...
#>   ..$ nh: int [1:21] 14 14 14 14 14 14 14 14 14 14 ...
#>   ..$ sc: num [1:21] 0.0622 0.0622 0.0622 0.0622 0.0622 ...
#>  $ :'data.frame':    21 obs. of  3 variables:
#>   ..$ cl: num [1:21] 0 0.05 0.1 0.15 0.2 0.25 0.3 0.35 0.4 0.45 ...
#>   ..$ nh: int [1:21] 2 2 2 2 2 1 1 1 1 0 ...
#>   ..$ sc: num [1:21] 0.000388 0.000388 0.000388 0.000388 0.000388 ...
#>  $ :'data.frame':    21 obs. of  3 variables:
#>   ..$ cl: num [1:21] 0 0.05 0.1 0.15 0.2 0.25 0.3 0.35 0.4 0.45 ...
#>   ..$ nh: int [1:21] 5 5 5 5 4 4 4 4 2 2 ...
#>   ..$ sc: num [1:21] 0.00271 0.00271 0.00271 0.00271 0.00236 ...
```

As you can see, we have a list of four data frames, one for each species. Each data frame has a set of values for each correleation threshold under consideration. The `cl` variable holds the correlation threshold, the `nh` values give the number of hits for each threshold, and the `sc` value is the sum of the ion counts for the peaks that are classed as a hit (we won't be using this variable for this analysis).

The main part of the classification is to combine this data into a score for each candidate species ID, following equation (2) in the paper. Scores are accumulated for each correlation threshold value. For each given correlation threshold and the number of 'hits' for that threshold for each species:

```
#initialise the scores for each sample
for(ss in 1:length(cld))
  cld[[ss]]$cumscore = 0

#for each correlation threshold
for(cl in 1:length(corlim)){

  #for each species
  for(ss in 1:length(cld)){

    #get the number of hits for this species
    nh <- cld[[ss]]$nh[cl]
    maxonh<-0

    #get the max number of hits for the other candidate species
    for(tt in 1:length(cld)){
      if(ss != tt){
        maxonh <- max(maxonh,cld[[tt]]$nh[cl])
      }
    }

    #add to the score if nh is greater than maxonh
    if(nh > maxonh){
      cld[[ss]]$cumscore[cl] <- (nh-maxonh)*corlim[cl]
    }
  }
}
```

With these values to hand, we can report the score and accumulate the scores for each species into a structure to hold the result:

```
result <- data.frame("id" = corlab, "score" = 0)

for(ss in 1:length(cld)){
  message(sprintf("Score for species %d (%s) = %f" ,ss,corlab[ss],sum(cld[[ss]]$cumscore)))
  result$score[ss] <- sum(cld[[ss]]$cumscore)
}
#> Score for species 1 (Sheep) = 0.000000
#> Score for species 2 (Cow) = 72.700000
#> Score for species 3 (Goat) = 0.000000
#> Score for species 4 (Deer) = 0.000000
```

This gives a score fore each species. In this case, the sample is unambiguously classified as cow. Sometimes, where a sample is noisy or contaminated, there can be non-zero scores for more than one species. *This is useful*, as it gives us an indication as to whether the automated classification is particularly strong or not.

The final stage in the analysis is to generate a graphical representation of the scoring process. Let's create that, and then discuss its features

```
title = sprintf("Sample '%s': manual ID: '%s'; Calc ID: '%s'\nscores ",sample$name,"unknown",result$id[[illegible]

for(ss in 1:nrow(result)){
  title = sprintf("%s %s = %0.3f",title,result$id[ss],result$score[ss])
}

par(mar = c(4,4,5,4))
plot(NA,xlab="Correlation Threshold",ylab = "Number of Hits",ylim=c(0,15),xlim = c(0,1), main = title)

points(x=corlim,y=cld[[1]]$nh,col="#e2ba5e")
points(x=corlim,y=cld[[2]]$nh,col="#3597c6")
points(x=corlim,y=cld[[3]]$nh,col="#e3645f")
points(x=corlim,y=cld[[4]]$nh,col="#793787")

lines(x=corlim,y=cld[[1]]$nh,col="#e2ba5e")
lines(x=corlim,y=cld[[2]]$nh,col="#3597c6")
lines(x=corlim,y=cld[[3]]$nh,col="#e3645f")
lines(x=corlim,y=cld[[4]]$nh,col="#793787")


legend("topright",legend = corlab,col=c("#e2ba5e","#3597c6","#e3645f","#793787"),lty = 1,pch=1)
```

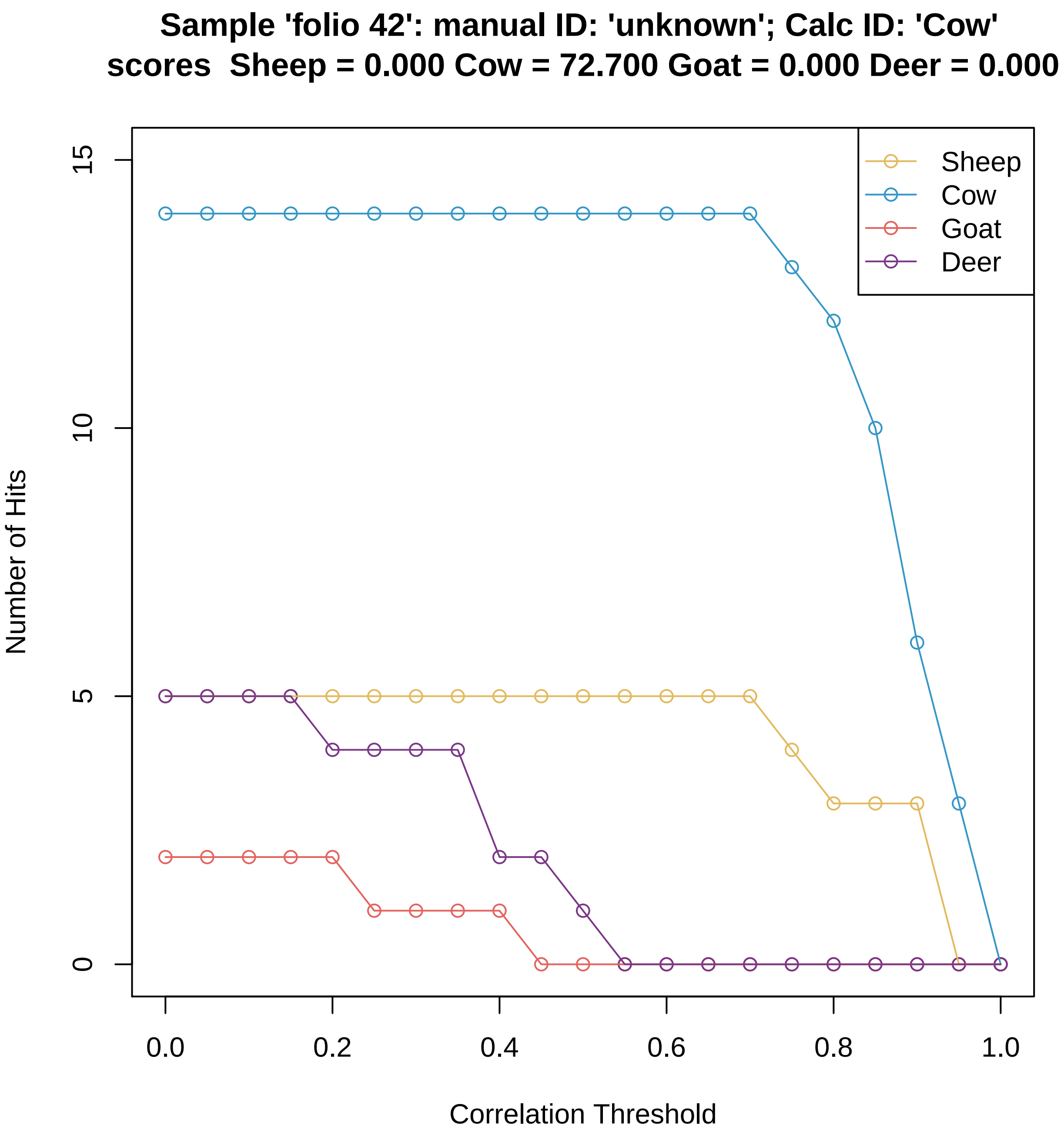

This gives us a clear graphical representation of the correlation scores. Let's make some observations about this plot:

- There are five peptides for each species and three replicates, so the maximum number of hits a sample can score is `3 x 5 = 15`.
- Note that we have set a limit on the amount of permissable lag in the alignment between observed and theoretical peaks to 0.6 Da. This is why the number of hits when the correlation threshold is zero is not 15.
- Cow has hits for 14 of the 15 peptides up to a correlation threshold of 0.7, so this is a pretty straightforward classification.
- Sheep has six peptides in common with cow - which is why the number of hits stays level at 5 to the same classification threshold.
- By contrast, goat and deer have *no* peptides in common with cow, so the number of hits diminishes with lower correlation thresholds.
