# Supplementary material for: How to get your goat: automated identification of species from MALDI-ToF spectra
Source: Bioinformatics. 2020 Mar 16;36(12):3719–25. doi: 10.1093/bioinformatics/btaa181 (PMC7320604; doi:10.1093/bioinformatics/btaa181)
Supplement: btaa181_Supplementary_Data [file btaa181_supplementary_data.zip › btaa181-Suppl_Data/corlim_analysis_incdeer_SI.pdf]

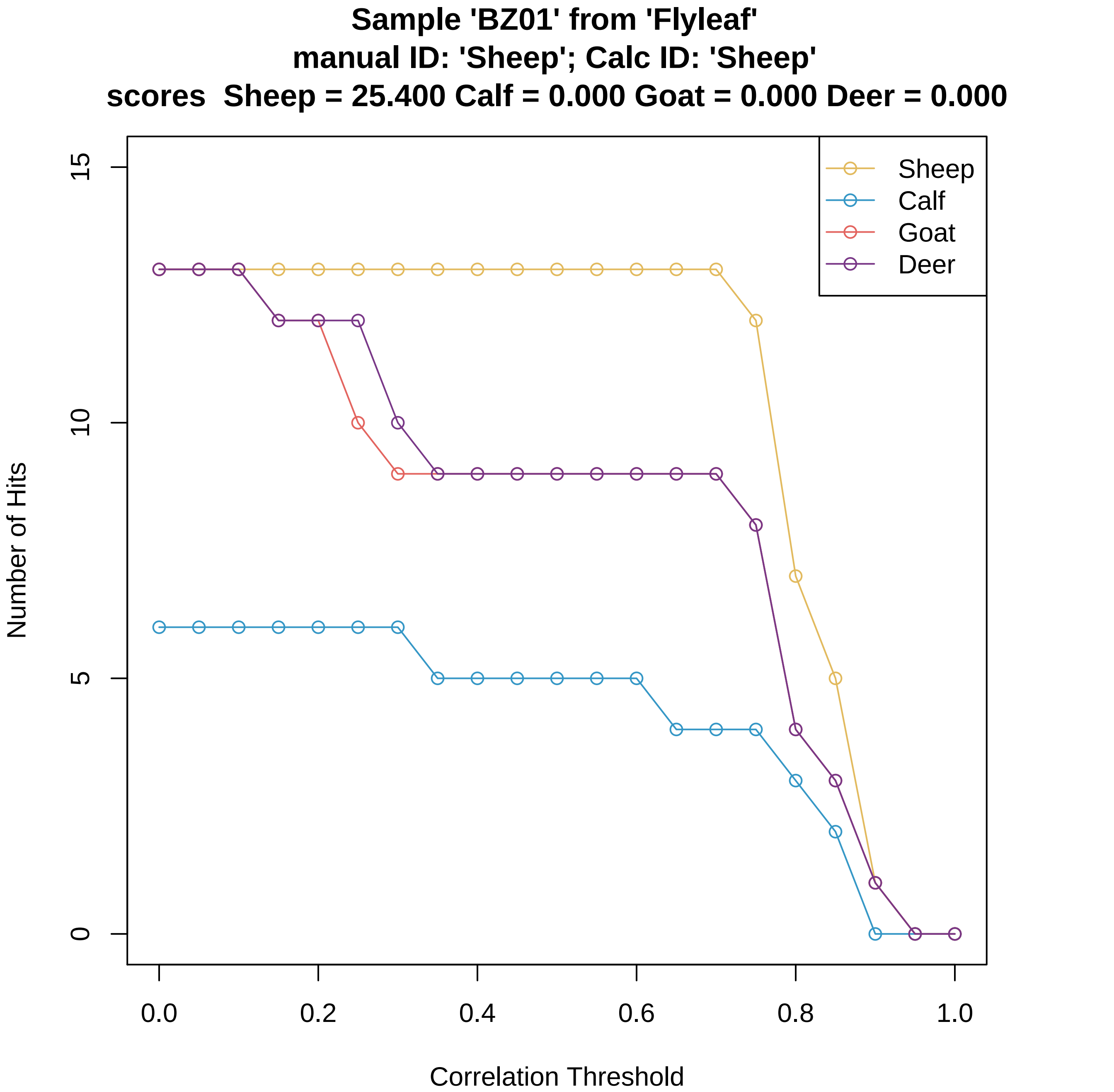

Sample 'BZ01' from 'Flyleaf'
manual ID: 'Sheep'; Calc ID: 'Sheep'
scores Sheep = 25.400 Calf = 0.000 Goat = 0.000 Deer = 0.000
Sheep
Calf
Goat
Deer
Number of Hits
0
5
10
15
0.0
0.2
0.4
0.6
0.8
1.0
Correlation Threshold

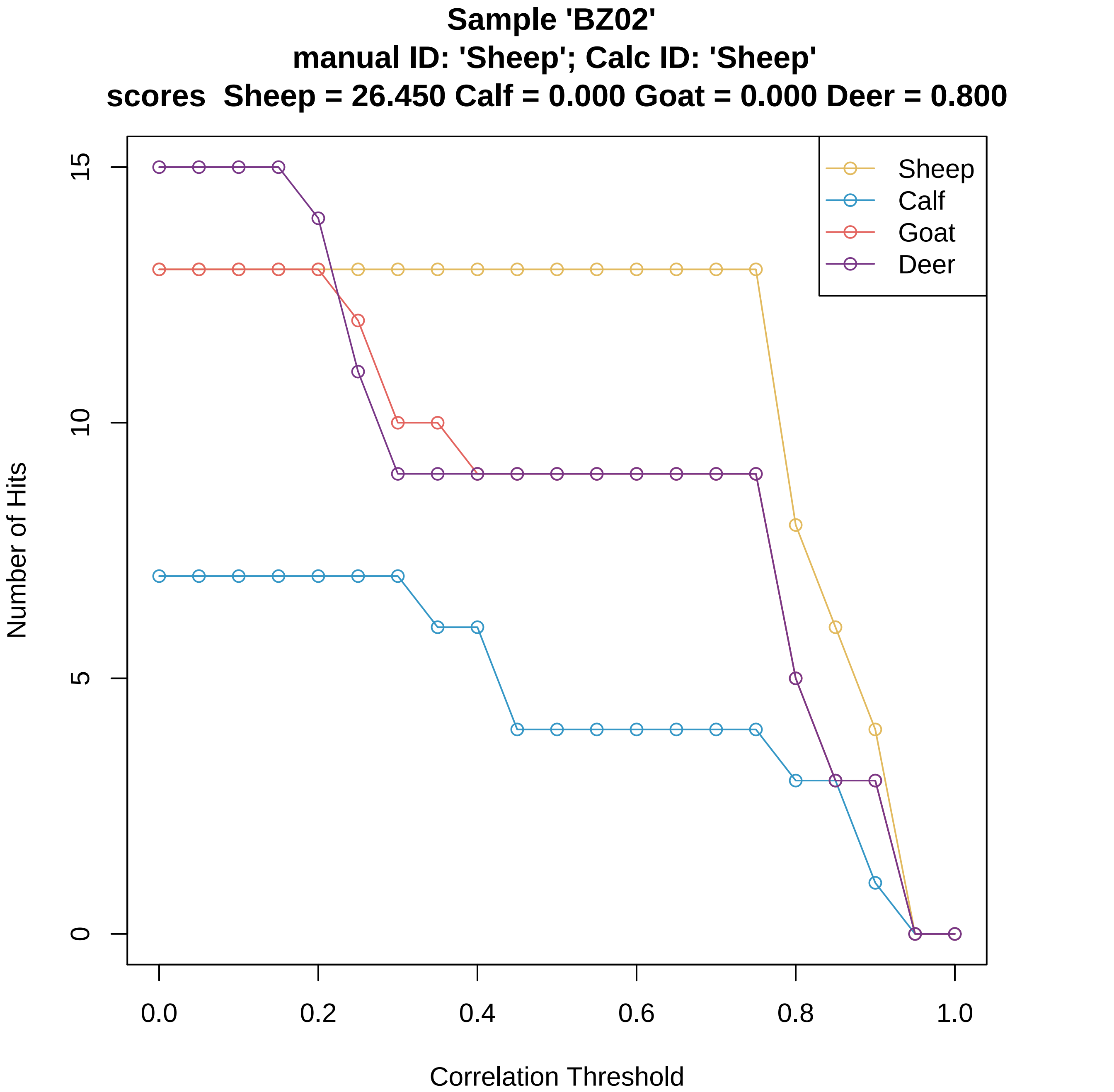
Sample 'BZ02'
manual ID: 'Sheep'; Calc ID: 'Sheep'
scores Sheep = 26.450 Calf = 0.000 Goat = 0.000 Deer = 0.800
Number of Hits
Correlation Threshold
Sheep
Calf
Goat
Deer
0
5
10
15
0.0
0.2
0.4
0.6
0.8
1.0

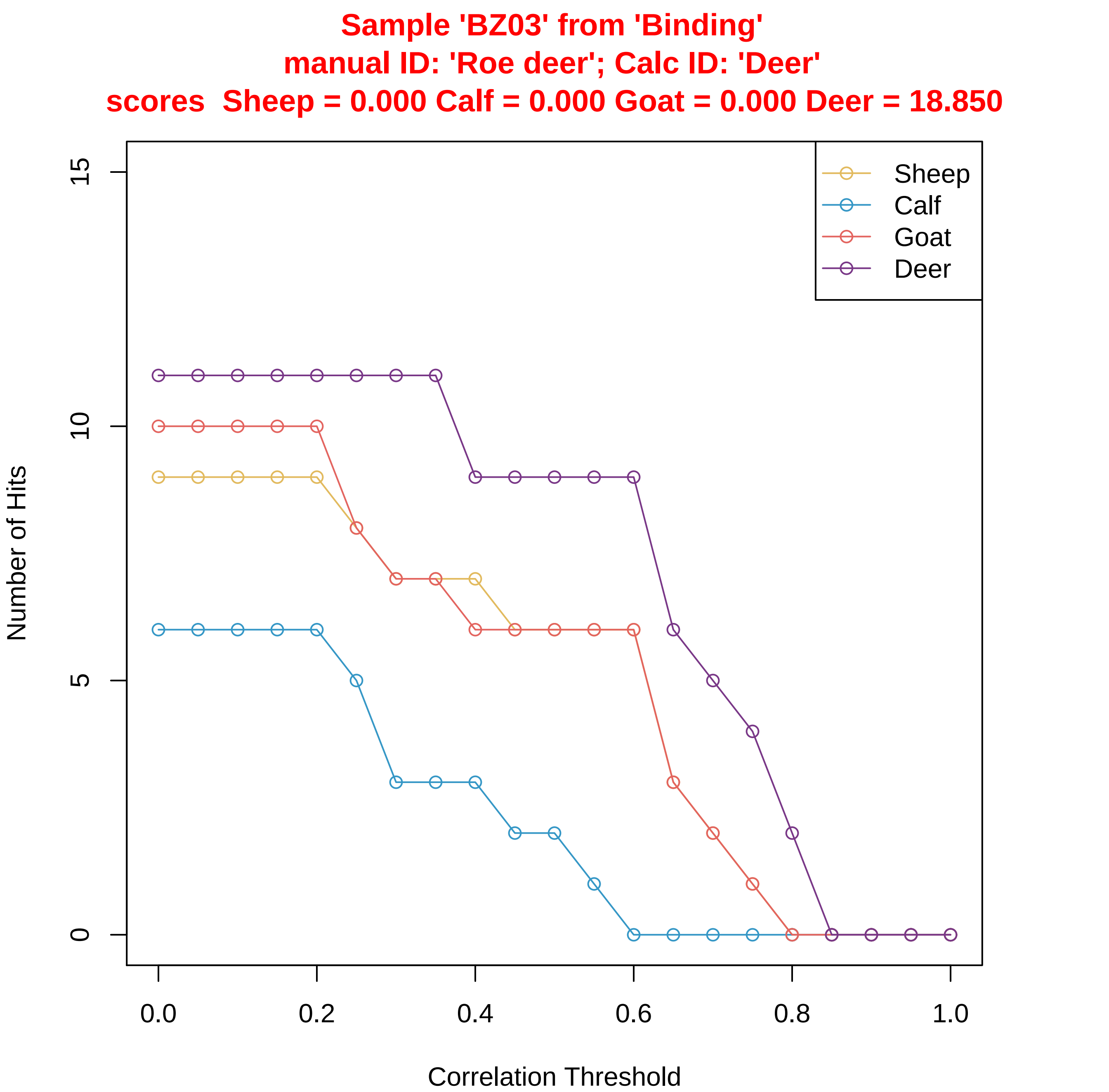
Sample 'BZ03' from 'Binding'
manual ID: 'Roe deer'; Calc ID: 'Deer'
scores Sheep = 0.000 Calf = 0.000 Goat = 0.000 Deer = 18.850
Sheep
Calf
Goat
Deer
Number of Hits
Correlation Threshold
0
5
10
15
0.0
0.2
0.4
0.6
0.8
1.0

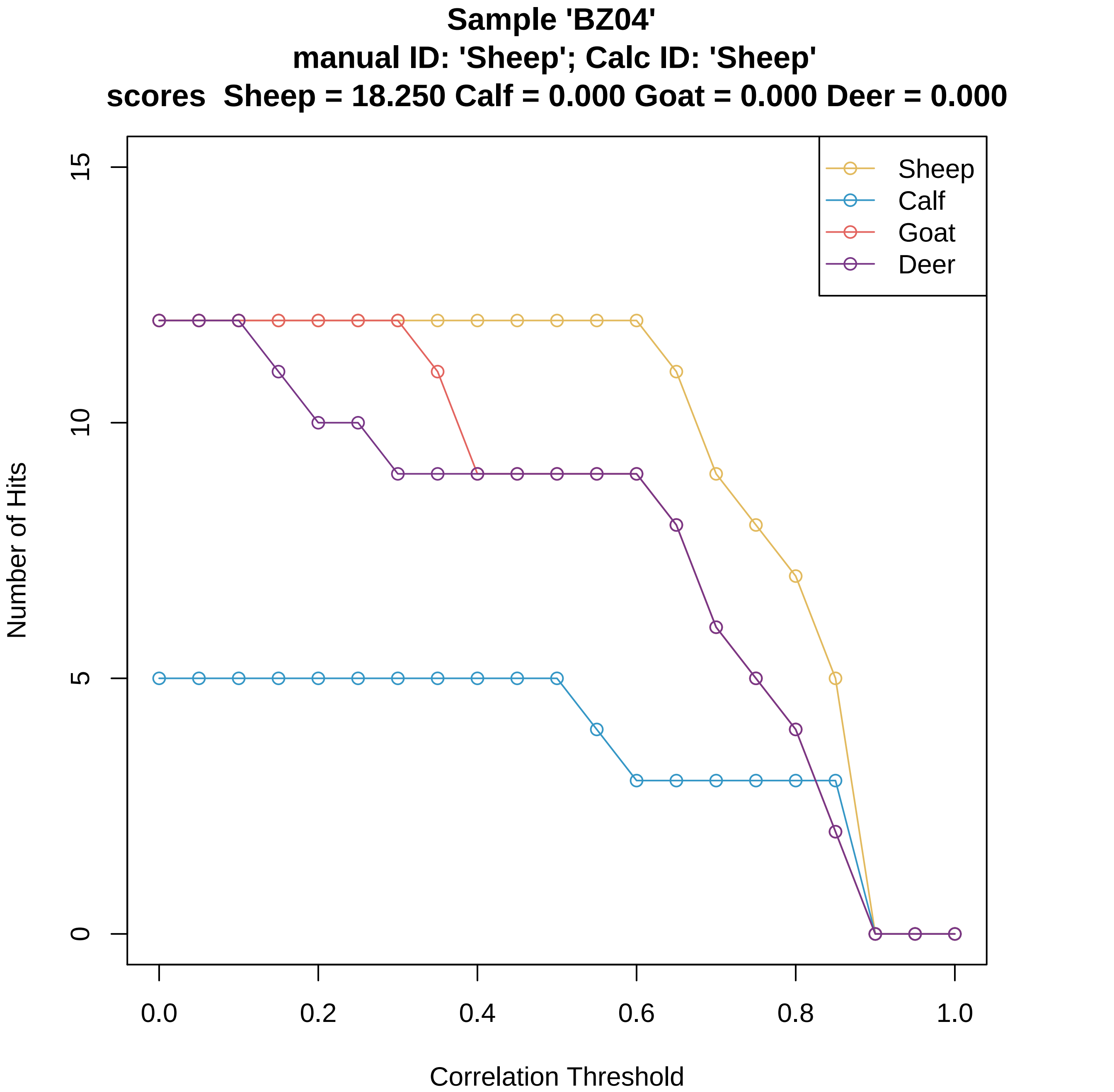
Sample 'BZ04'
manual ID: 'Sheep'; Calc ID: 'Sheep'
scores Sheep = 18.250 Calf = 0.000 Goat = 0.000 Deer = 0.000
Sheep
Calf
Goat
Deer
Number of Hits
0
5
10
15
0.0
0.2
0.4
0.6
0.8
1.0
Correlation Threshold

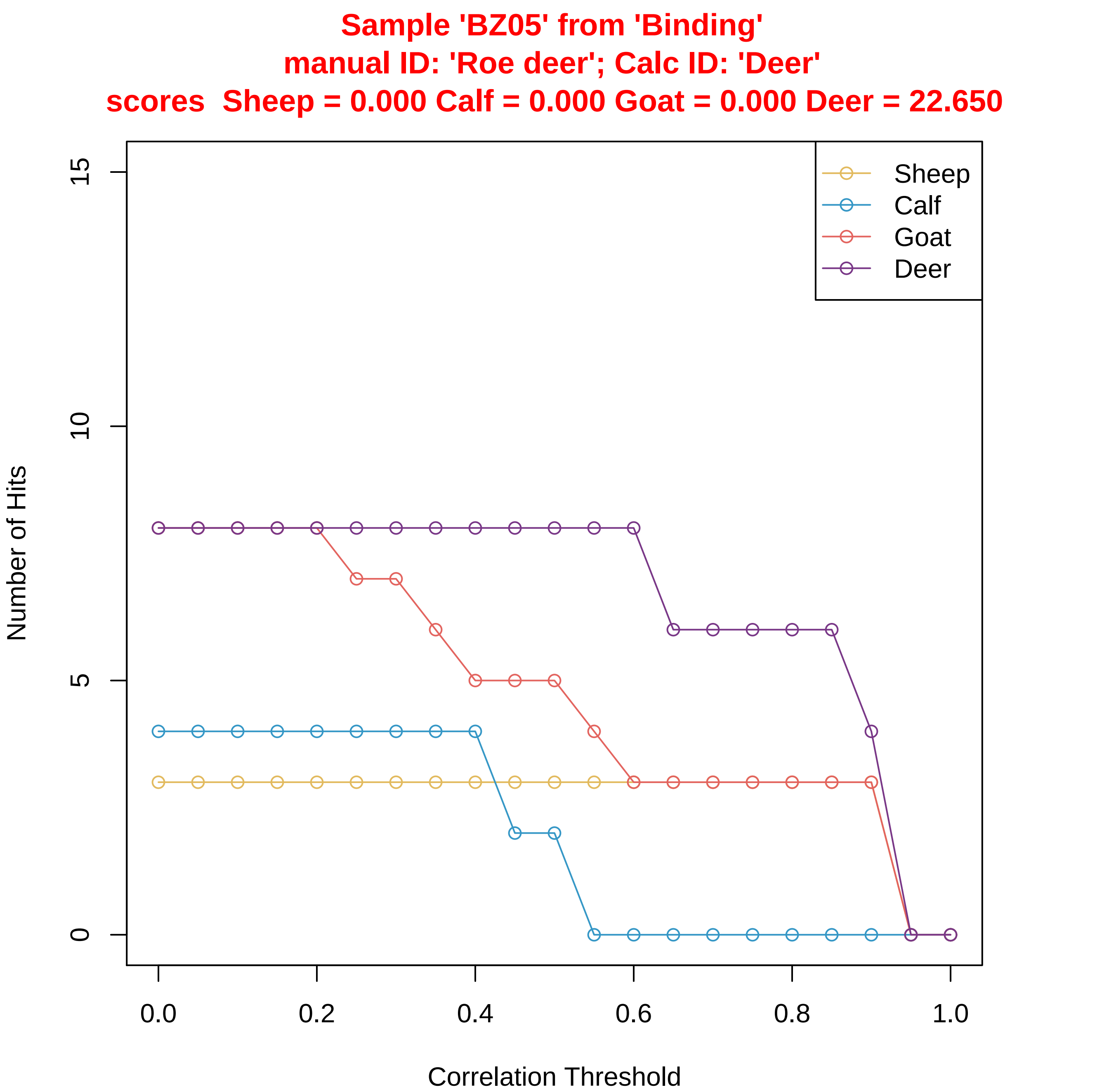
Sample 'BZ05' from 'Binding'
manual ID: 'Roe deer'; Calc ID: 'Deer'
scores Sheep = 0.000 Calf = 0.000 Goat = 0.000 Deer = 22.650
Sheep
Calf
Goat
Deer
Number of Hits
Correlation Threshold
0
5
10
15
0.0
0.2
0.4
0.6
0.8
1.0

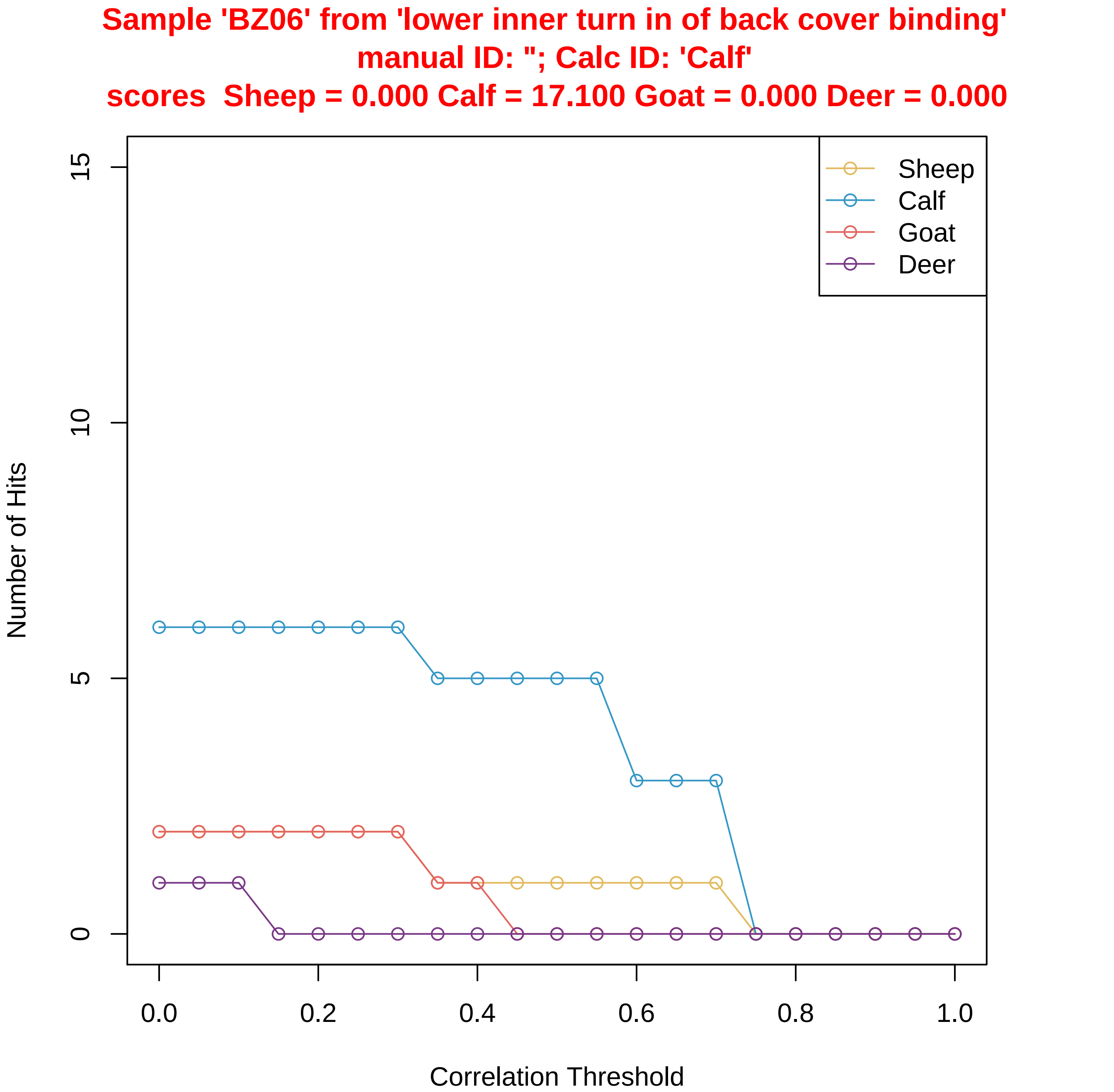

Sample 'BZ06' from 'lower inner turn in of back cover binding'
manual ID: ''; Calc ID: 'Calf'
scores Sheep = 0.000 Calf = 17.100 Goat = 0.000 Deer = 0.000
Sheep
Calf
Goat
Deer
Number of Hits
0
5
10
15
0.0
0.2
0.4
0.6
0.8
1.0
Correlation Threshold

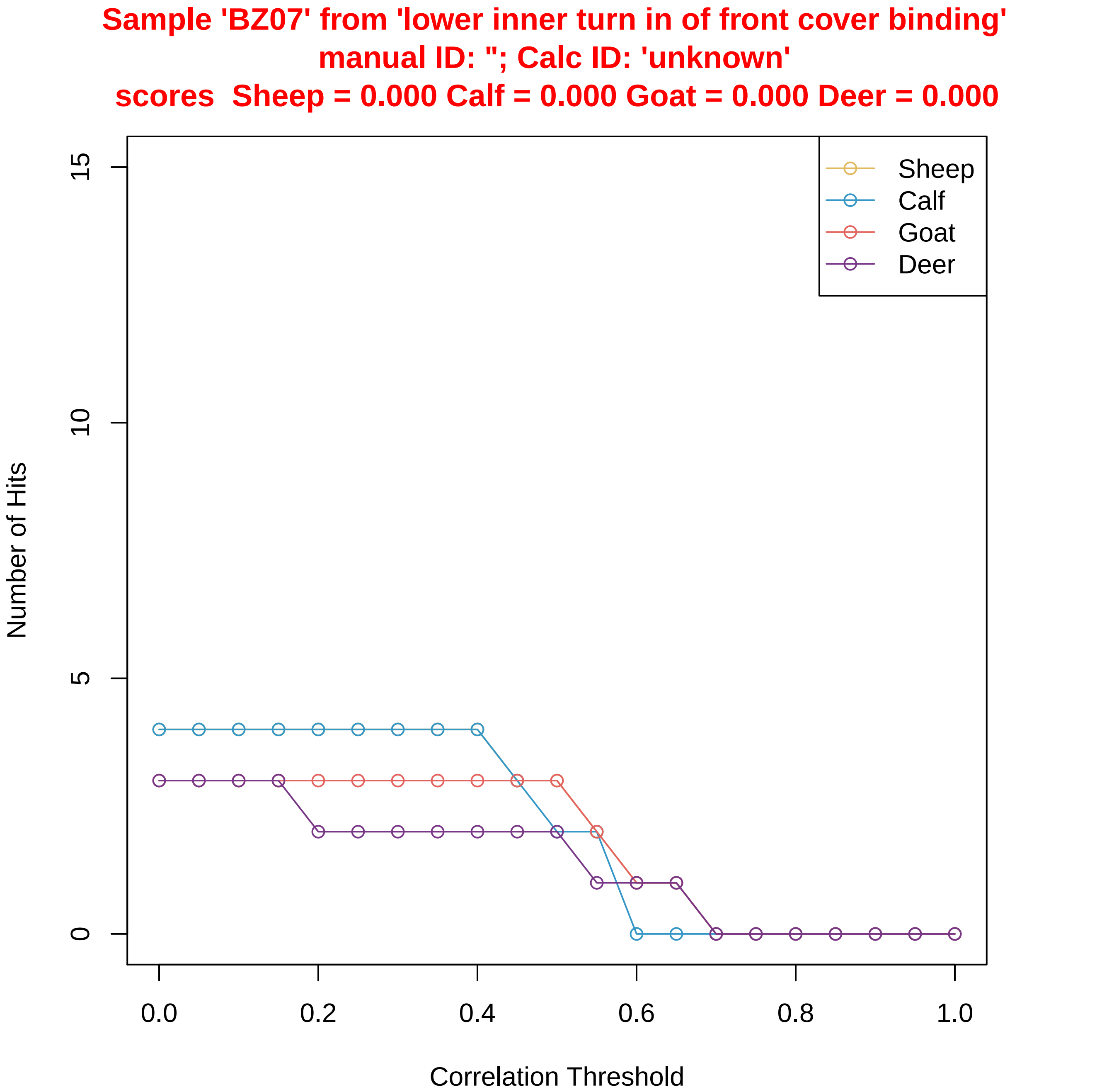

Sample 'BZ07' from 'lower inner turn in of front cover binding'
manual ID: ''; Calc ID: 'unknown'
scores Sheep = 0.000 Calf = 0.000 Goat = 0.000 Deer = 0.000
Sheep
Calf
Goat
Deer
Number of Hits
Correlation Threshold
0
5
10
15
0.0
0.2
0.4
0.6
0.8
1.0

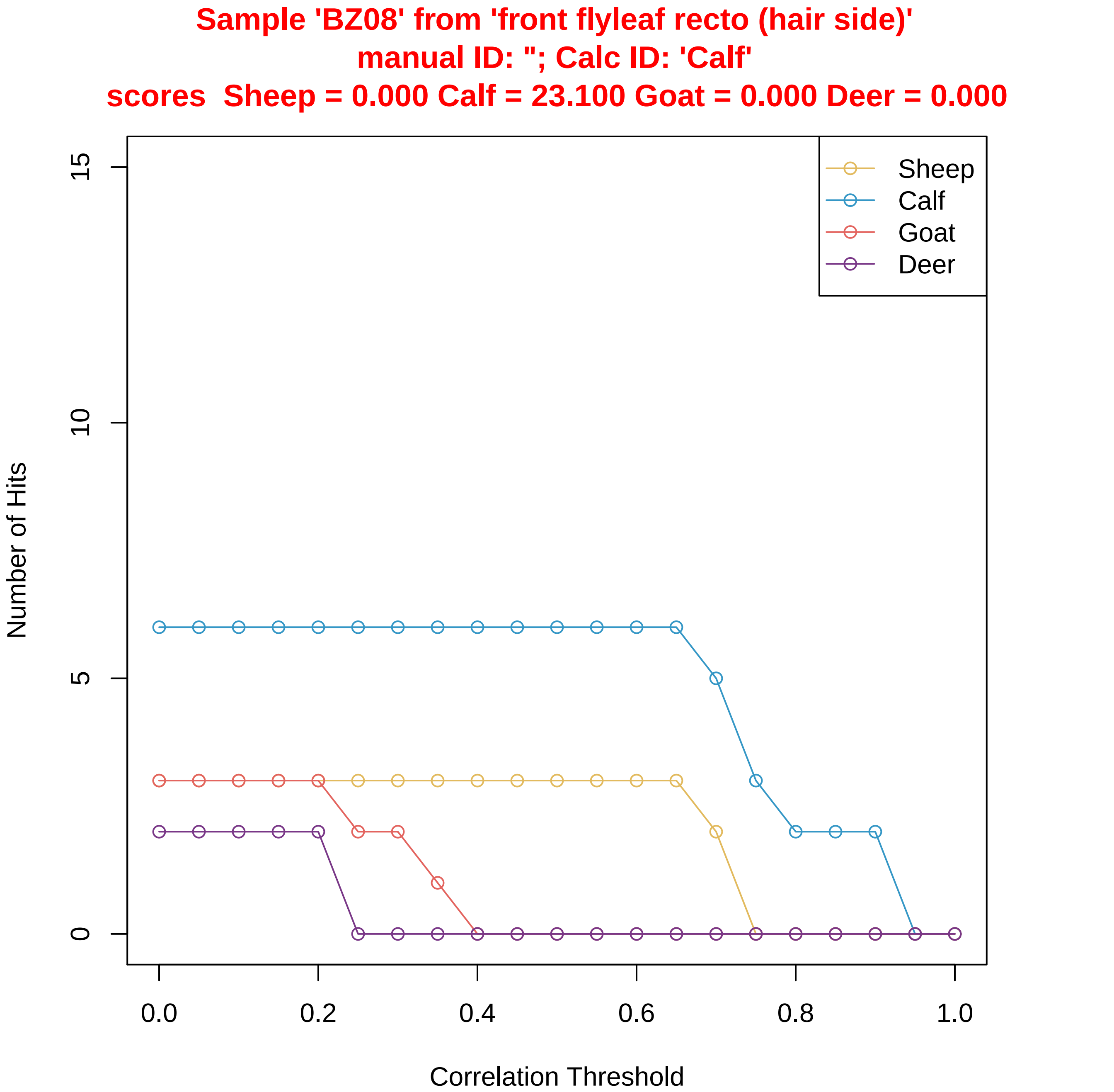
Sample 'BZ08' from 'front flyleaf recto (hair side)'
manual ID: ''; Calc ID: 'Calf'
scores Sheep = 0.000 Calf = 23.100 Goat = 0.000 Deer = 0.000
Sheep
Calf
Goat
Deer
Number of Hits
0
5
10
15
Correlation Threshold
0.0
0.2
0.4
0.6
0.8
1.0

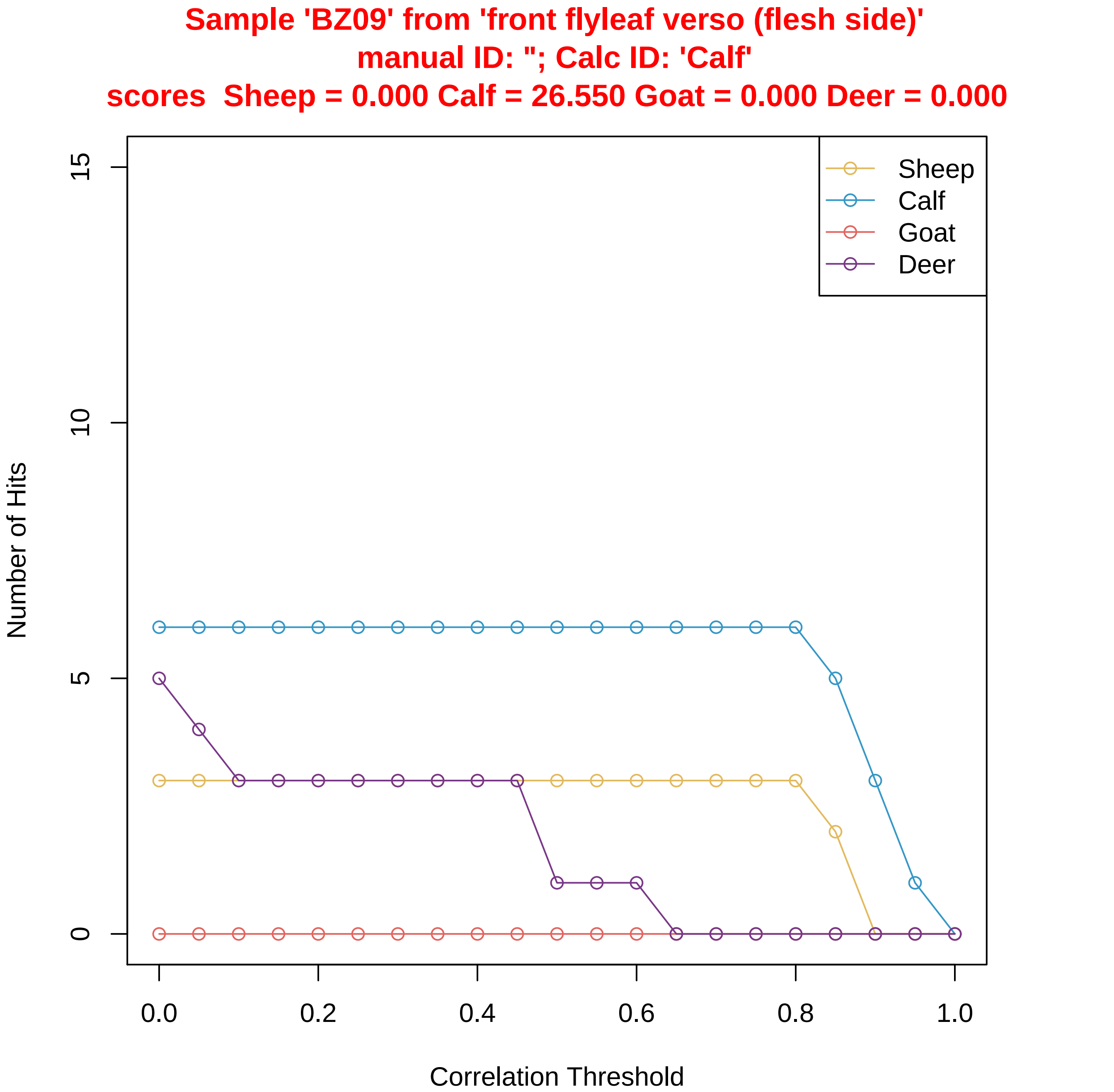

Sample 'BZ09' from 'front flyleaf verso (flesh side)'
manual ID: ''; Calc ID: 'Calf'
scores Sheep = 0.000 Calf = 26.550 Goat = 0.000 Deer = 0.000
Sheep
Calf
Goat
Deer
0
5
10
15
Number of Hits
0.0
0.2
0.4
0.6
0.8
1.0
Correlation Threshold

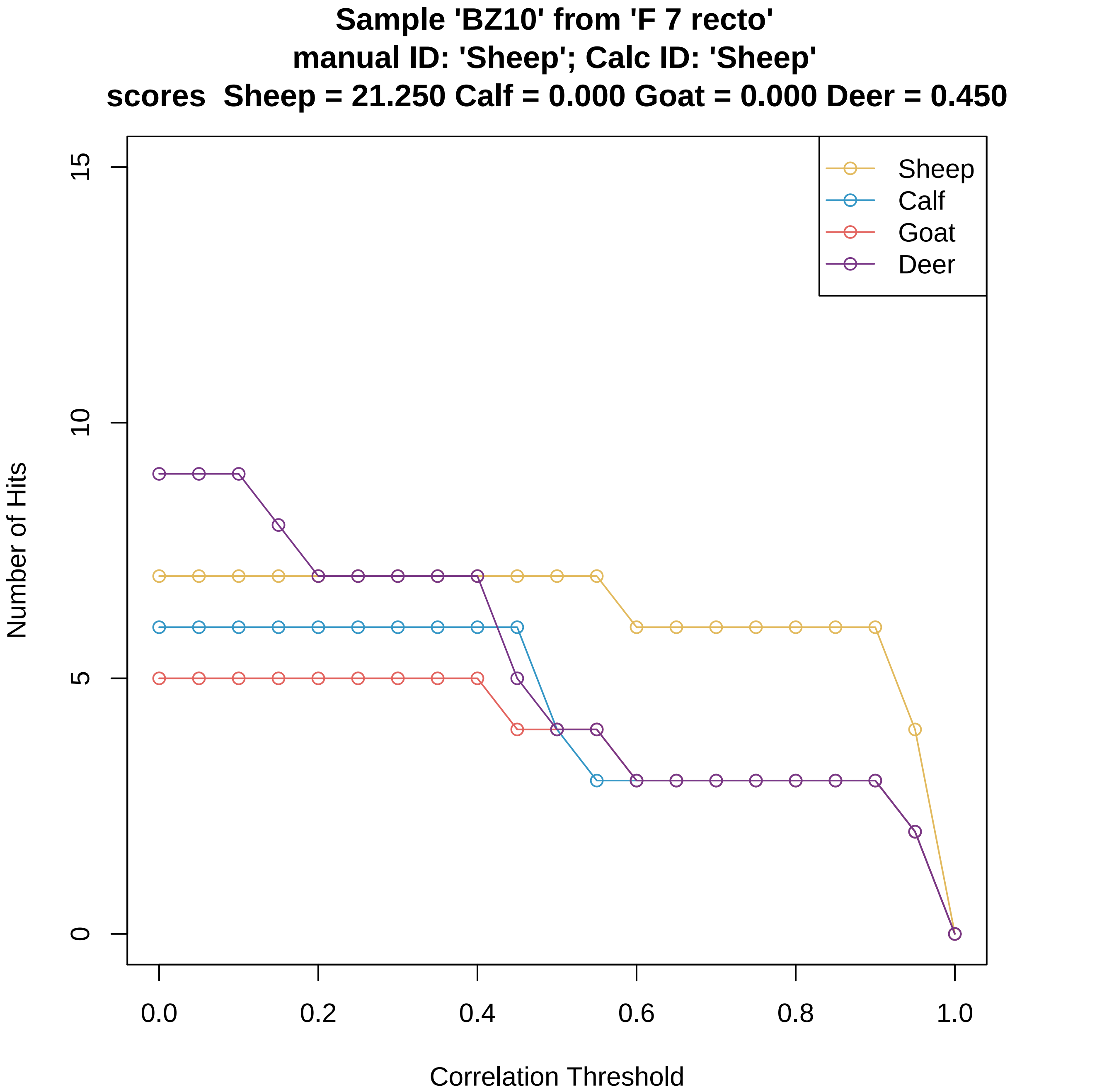
Sample 'BZ10' from 'F 7 recto'
manual ID: 'Sheep'; Calc ID: 'Sheep'
scores Sheep = 21.250 Calf = 0.000 Goat = 0.000 Deer = 0.450
Sheep
Calf
Goat
Deer
Number of Hits
0
5
10
15
0.0
0.2
0.4
0.6
0.8
1.0
Correlation Threshold

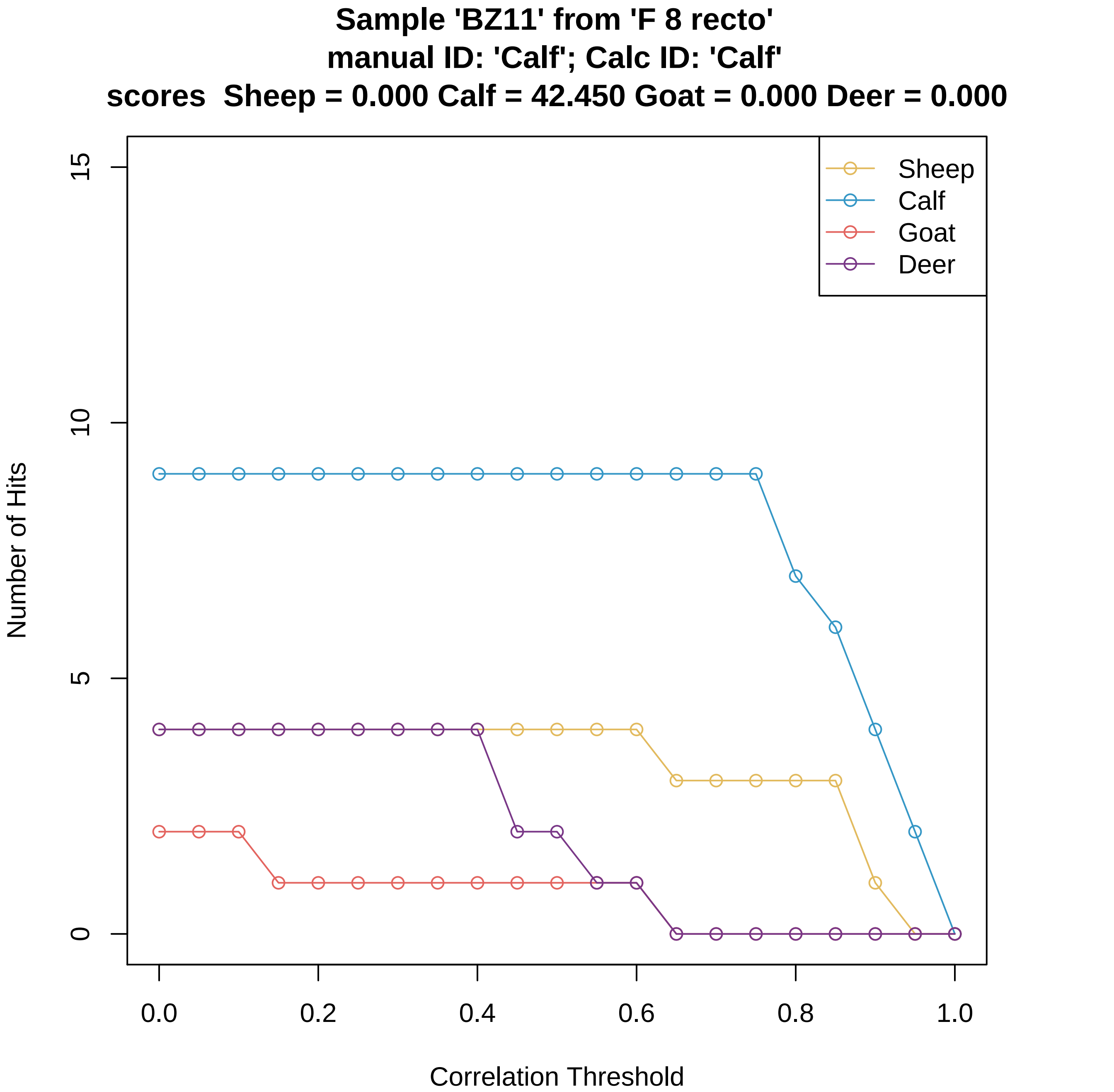
Sample 'BZ11' from 'F 8 recto'
manual ID: 'Calf'; Calc ID: 'Calf'
scores Sheep = 0.000 Calf = 42.450 Goat = 0.000 Deer = 0.000
Sheep
Calf
Goat
Deer
Number of Hits
0
5
10
15
0.0
0.2
0.4
0.6
0.8
1.0
Correlation Threshold

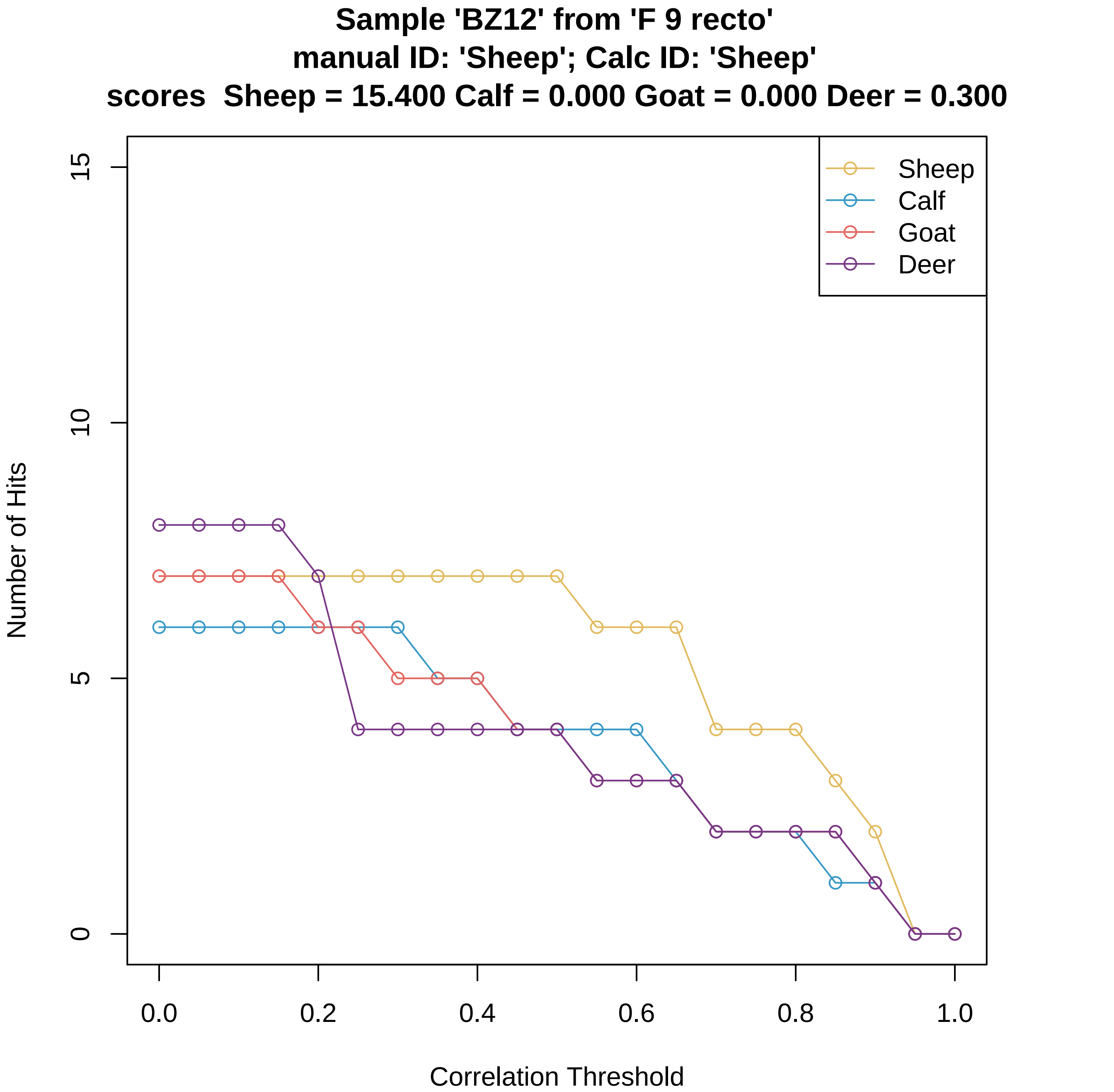
Sample 'BZ12' from 'F 9 recto'
manual ID: 'Sheep'; Calc ID: 'Sheep'
scores Sheep = 15.400 Calf = 0.000 Goat = 0.000 Deer = 0.300
Sheep
Calf
Goat
Deer
Number of Hits
Correlation Threshold
0
5
10
15
0.0
0.2
0.4
0.6
0.8
1.0

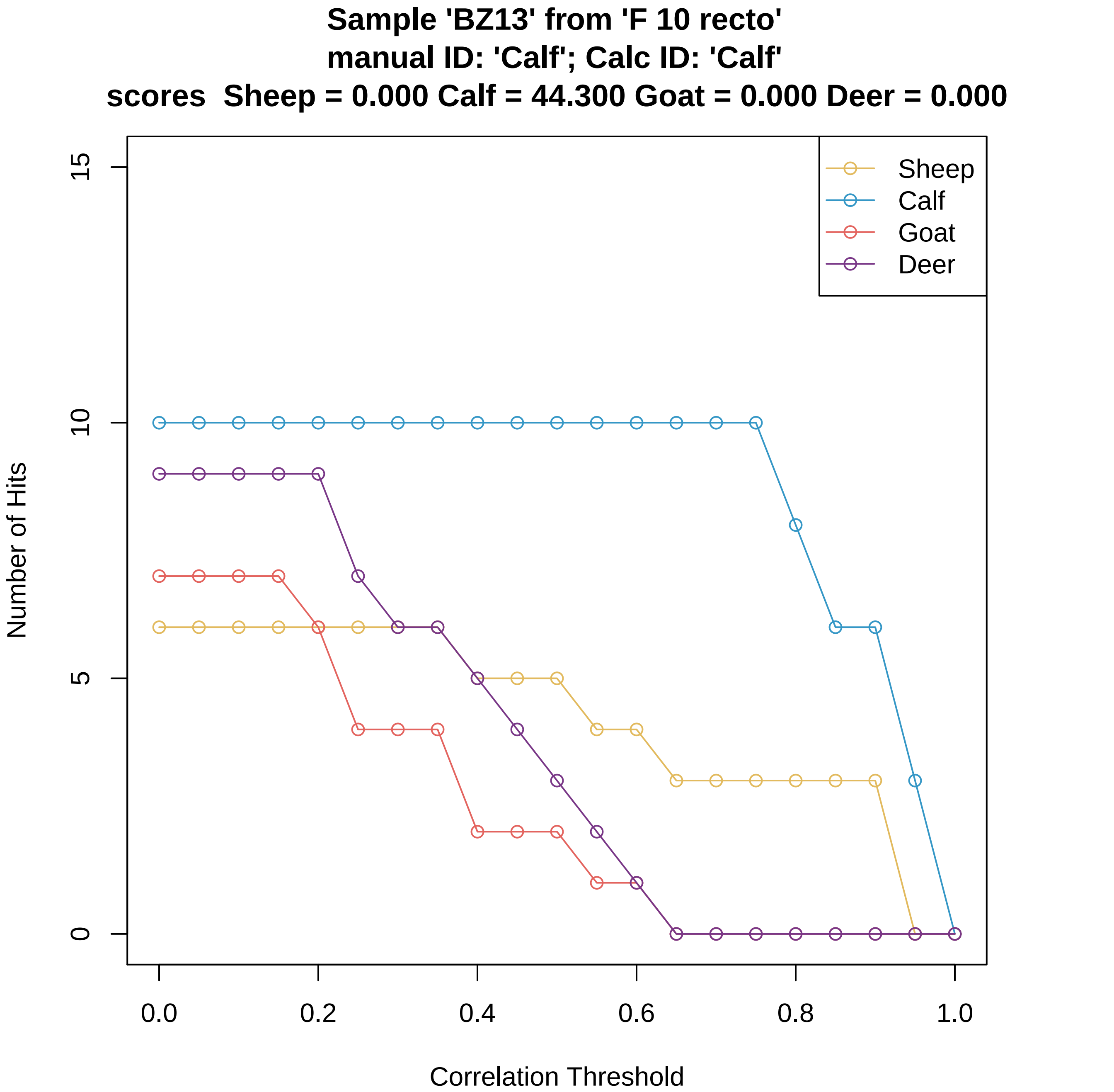
Sample 'BZ13' from 'F 10 recto'
manual ID: 'Calf'; Calc ID: 'Calf'
scores Sheep = 0.000 Calf = 44.300 Goat = 0.000 Deer = 0.000
Sheep
Calf
Goat
Deer
Number of Hits
0
5
10
15
0.0
0.2
0.4
0.6
0.8
1.0
Correlation Threshold

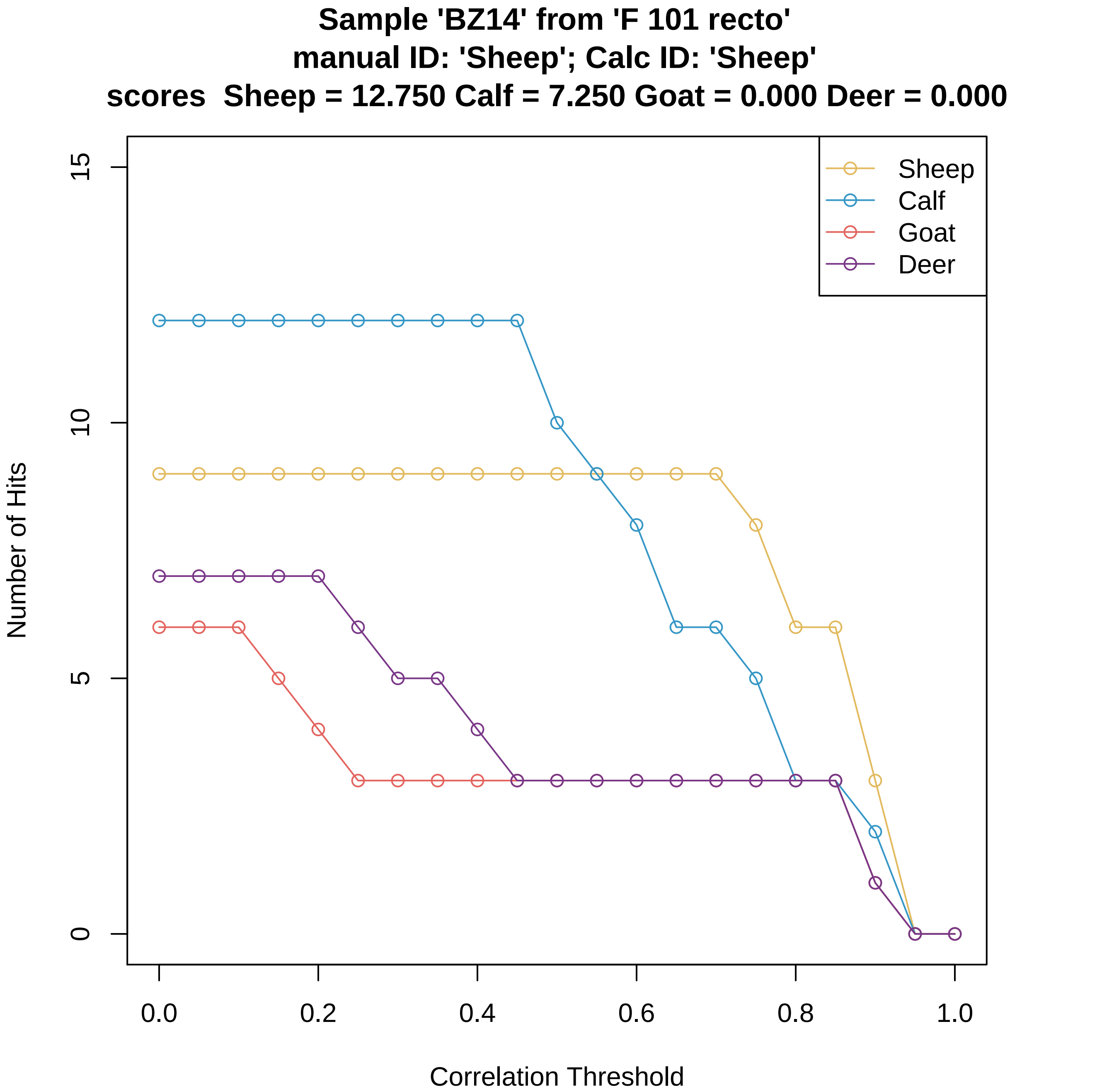
Sample 'BZ14' from 'F 101 recto'
manual ID: 'Sheep'; Calc ID: 'Sheep'
scores Sheep = 12.750 Calf = 7.250 Goat = 0.000 Deer = 0.000
Sheep
Calf
Goat
Deer
Number of Hits
0
5
10
15
0.0
0.2
0.4
0.6
0.8
1.0
Correlation Threshold

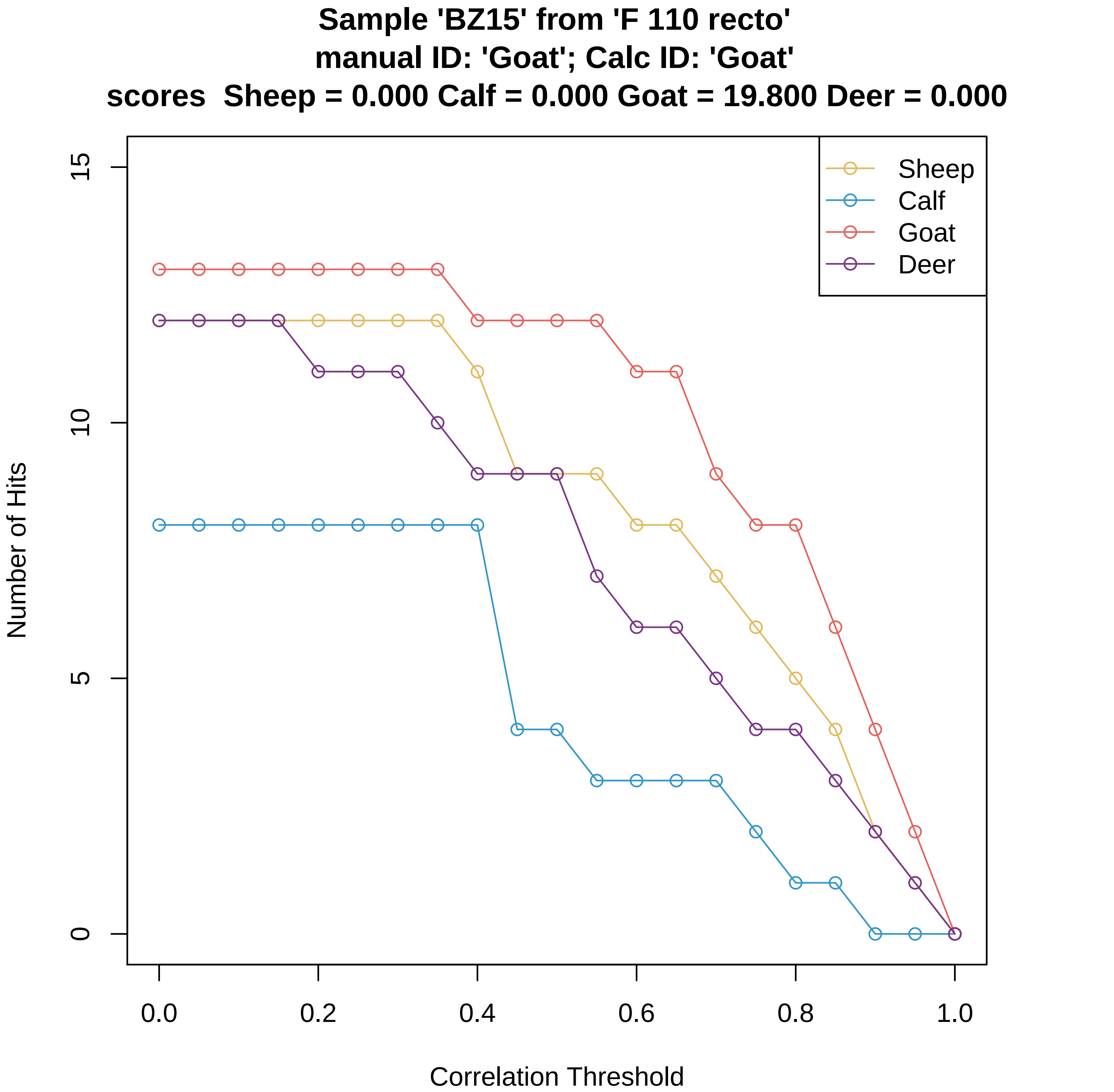
Sample 'BZ15' from 'F 110 recto'
manual ID: 'Goat'; Calc ID: 'Goat'
scores Sheep = 0.000 Calf = 0.000 Goat = 19.800 Deer = 0.000
Sheep
Calf
Goat
Deer
Number of Hits
0
5
10
15
0.0
0.2
0.4
0.6
0.8
1.0
Correlation Threshold

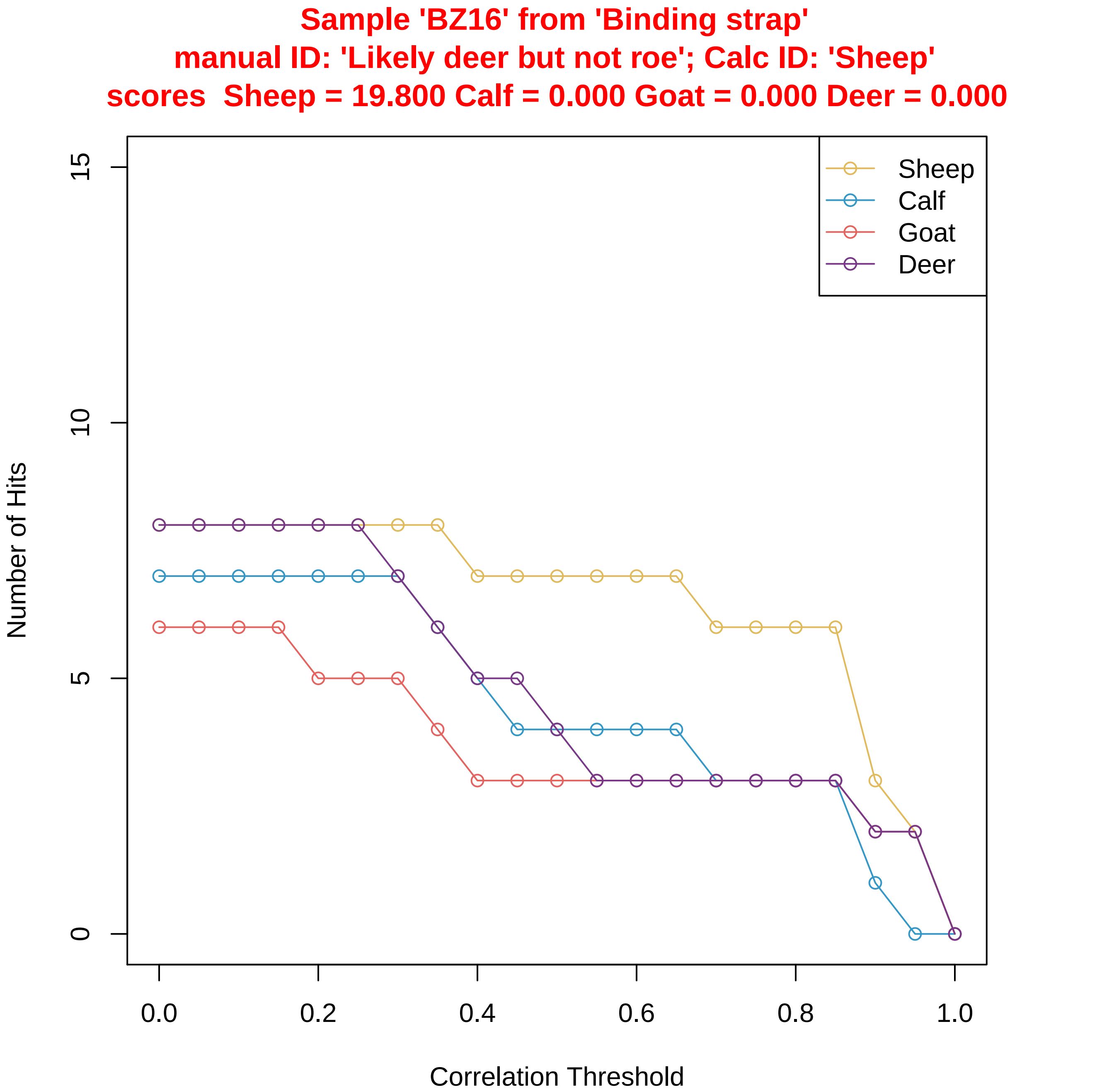
Sample 'BZ16' from 'Binding strap'
manual ID: 'Likely deer but not roe'; Calc ID: 'Sheep'
scores Sheep = 19.800 Calf = 0.000 Goat = 0.000 Deer = 0.000
Sheep
Calf
Goat
Deer
Number of Hits
Correlation Threshold
0
5
10
15
0.0
0.2
0.4
0.6
0.8
1.0

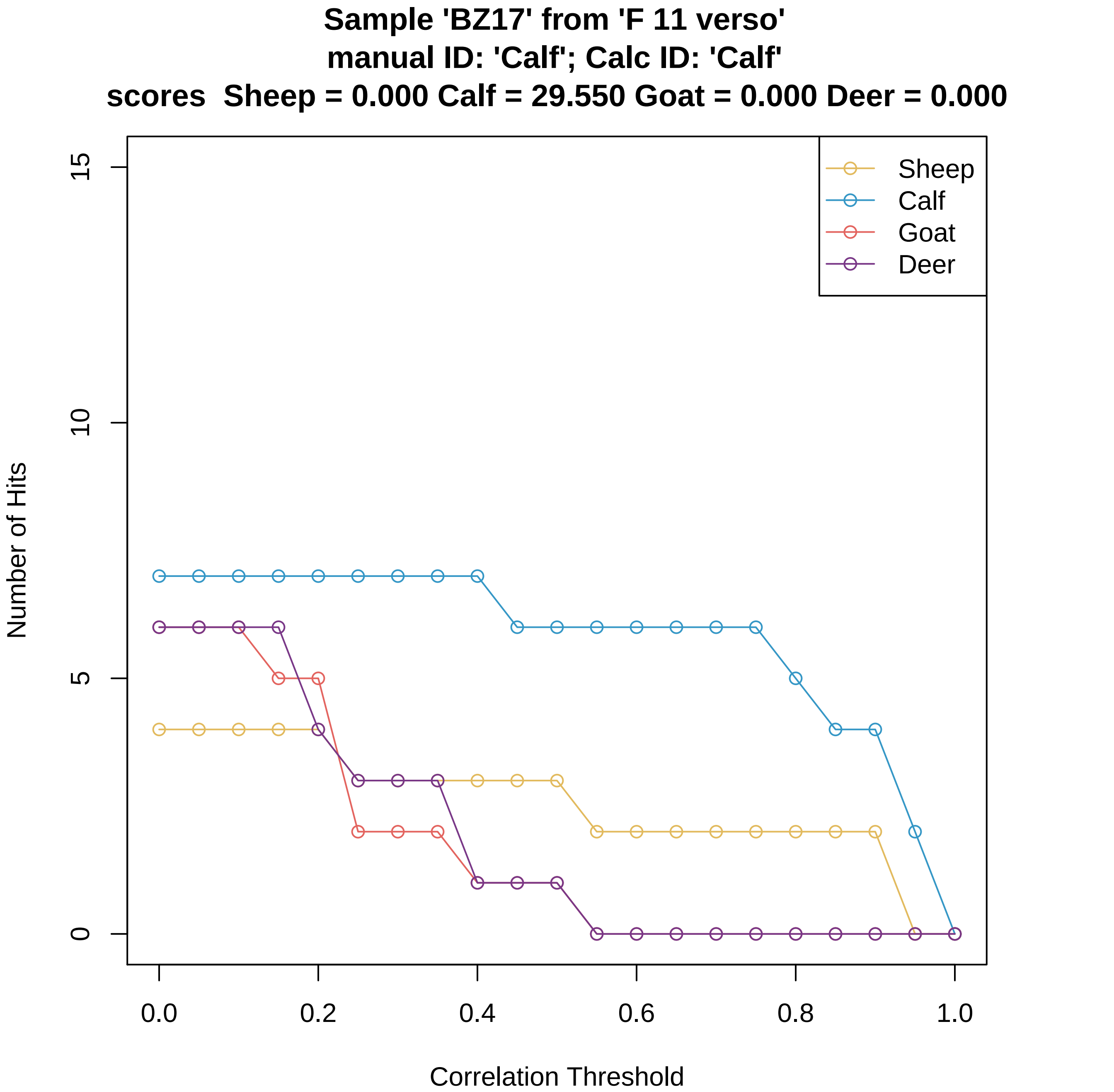

Sample 'BZ17' from 'F 11 verso'
manual ID: 'Calf'; Calc ID: 'Calf'
scores Sheep = 0.000 Calf = 29.550 Goat = 0.000 Deer = 0.000
Sheep
Calf
Goat
Deer
Number of Hits
0
5
10
15
0.0
0.2
0.4
0.6
0.8
1.0
Correlation Threshold

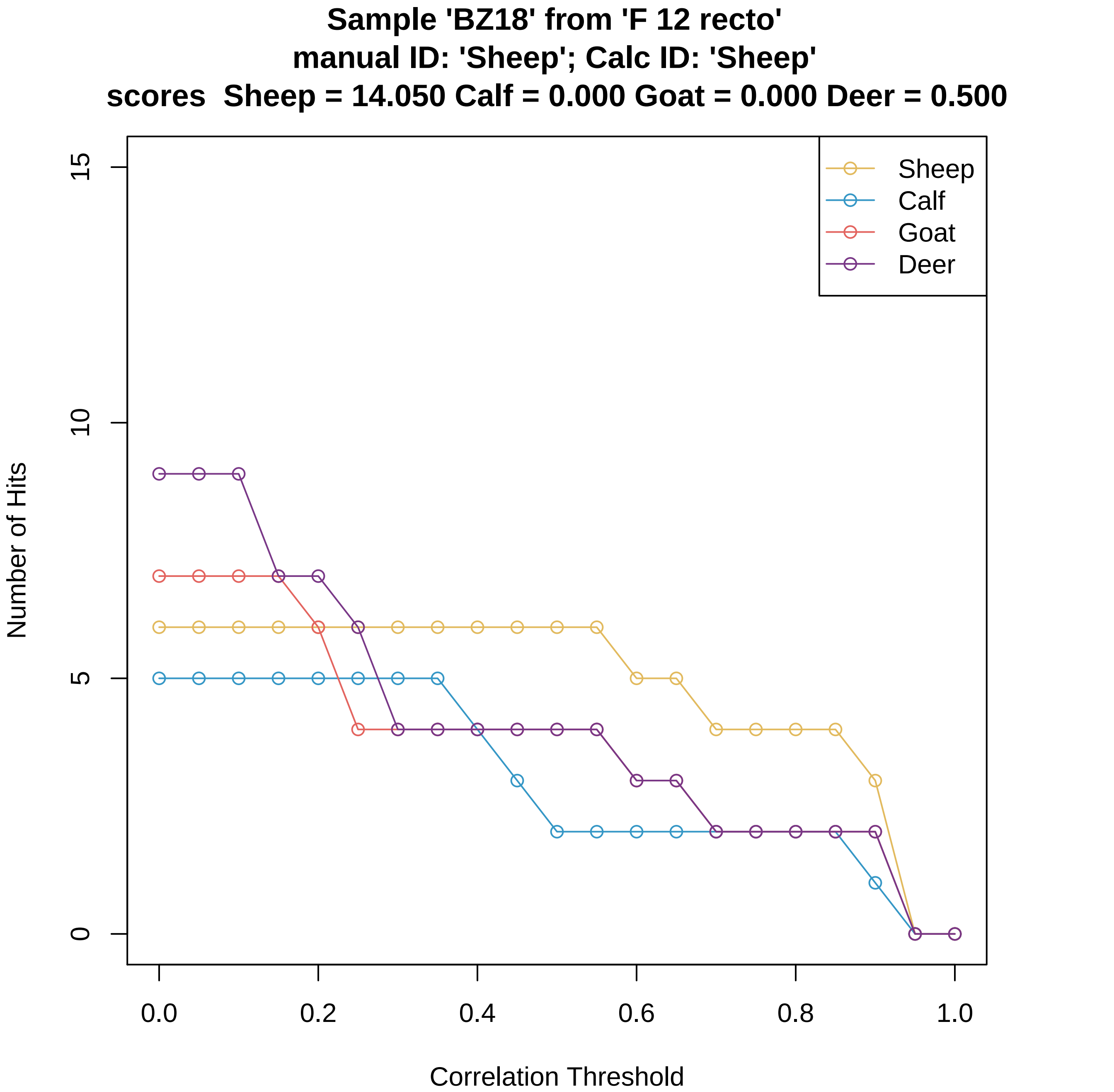

Sample 'BZ18' from 'F 12 recto'
manual ID: 'Sheep'; Calc ID: 'Sheep'
scores Sheep = 14.050 Calf = 0.000 Goat = 0.000 Deer = 0.500
Sheep
Calf
Goat
Deer
Number of Hits
0
5
10
15
Correlation Threshold
0.0
0.2
0.4
0.6
0.8
1.0

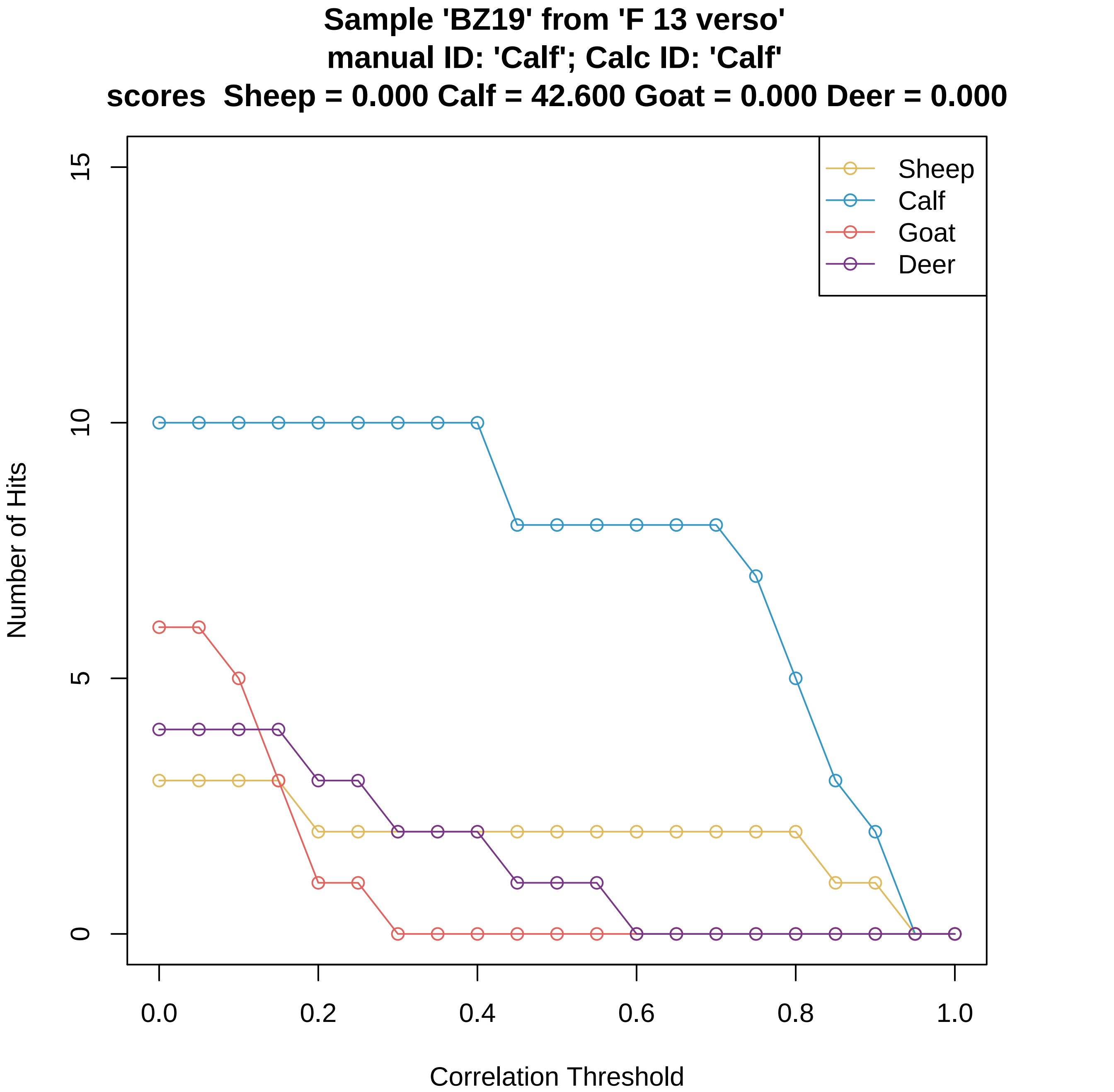

Sample 'BZ19' from 'F 13 verso'
manual ID: 'Calf'; Calc ID: 'Calf'
scores Sheep = 0.000 Calf = 42.600 Goat = 0.000 Deer = 0.000
Sheep
Calf
Goat
Deer
Number of Hits
0
5
10
15
0.0
0.2
0.4
0.6
0.8
1.0
Correlation Threshold

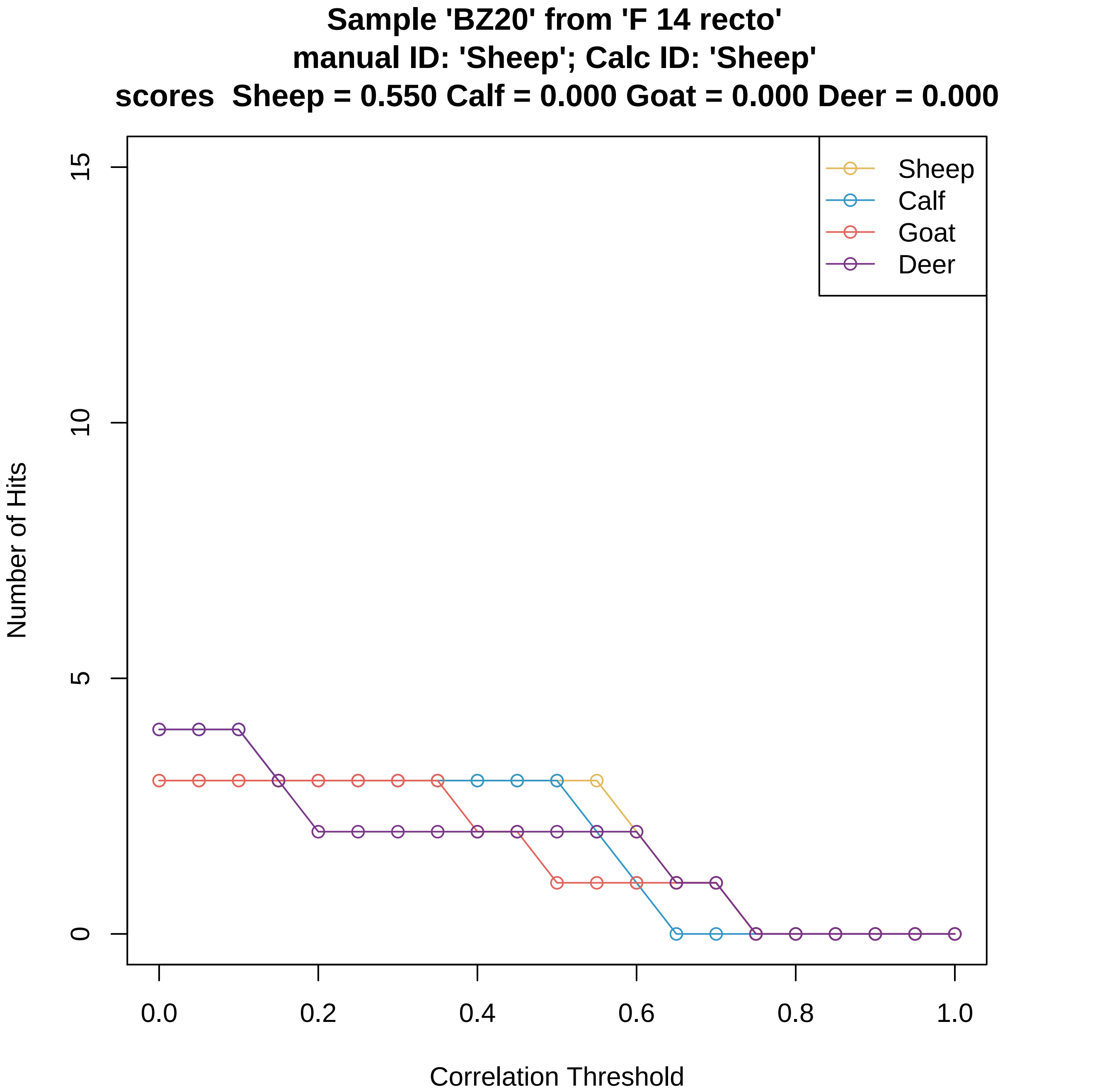

Sample 'BZ20' from 'F 14 recto'
manual ID: 'Sheep'; Calc ID: 'Sheep'
scores Sheep = 0.550 Calf = 0.000 Goat = 0.000 Deer = 0.000
Sheep
Calf
Goat
Deer
Number of Hits
0
5
10
15
0.0
0.2
0.4
0.6
0.8
1.0
Correlation Threshold

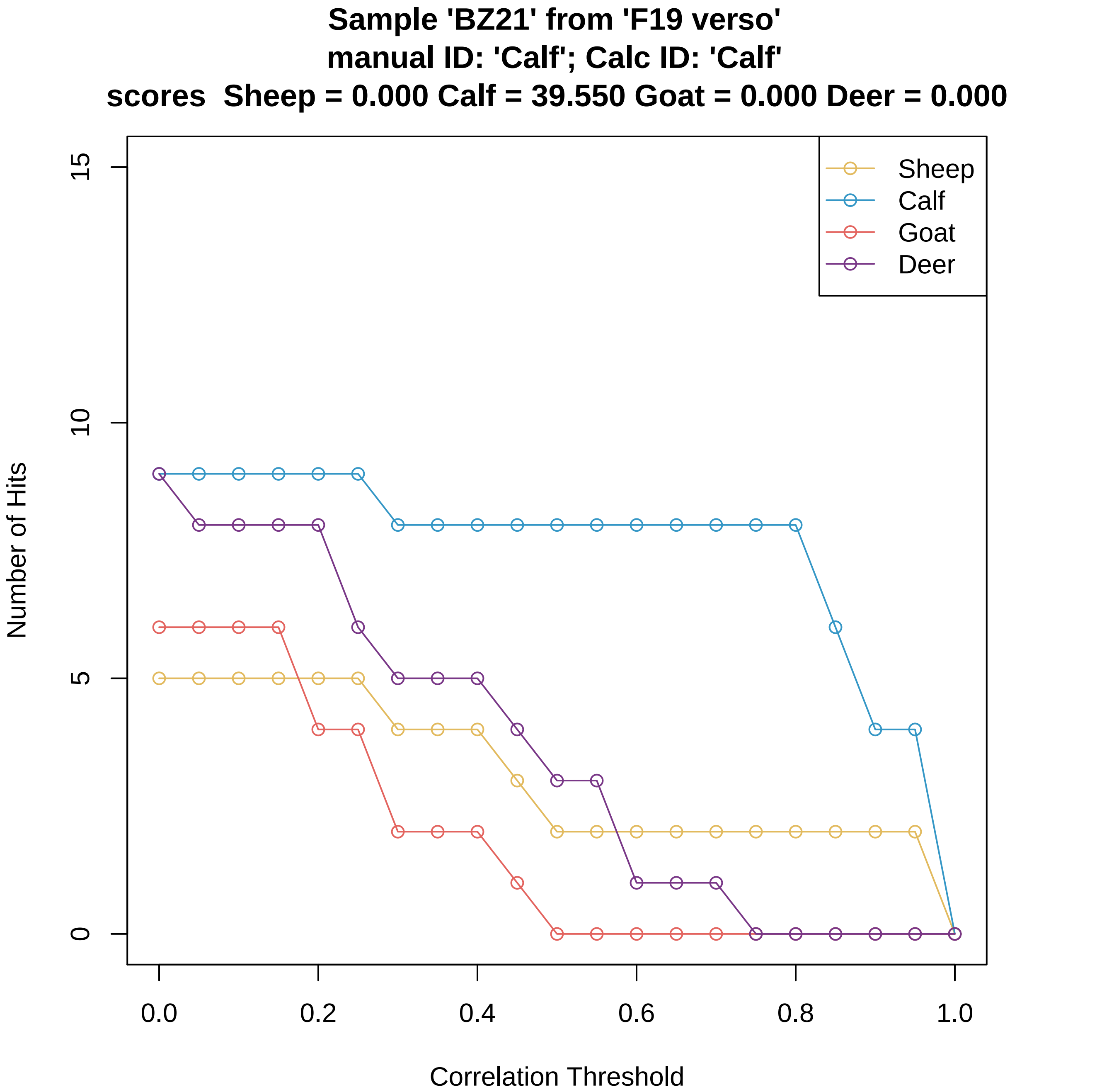

Sample 'BZ21' from 'F19 verso'
manual ID: 'Calf'; Calc ID: 'Calf'
scores Sheep = 0.000 Calf = 39.550 Goat = 0.000 Deer = 0.000
Sheep
Calf
Goat
Deer
0
5
10
15
Number of Hits
0.0
0.2
0.4
0.6
0.8
1.0
Correlation Threshold

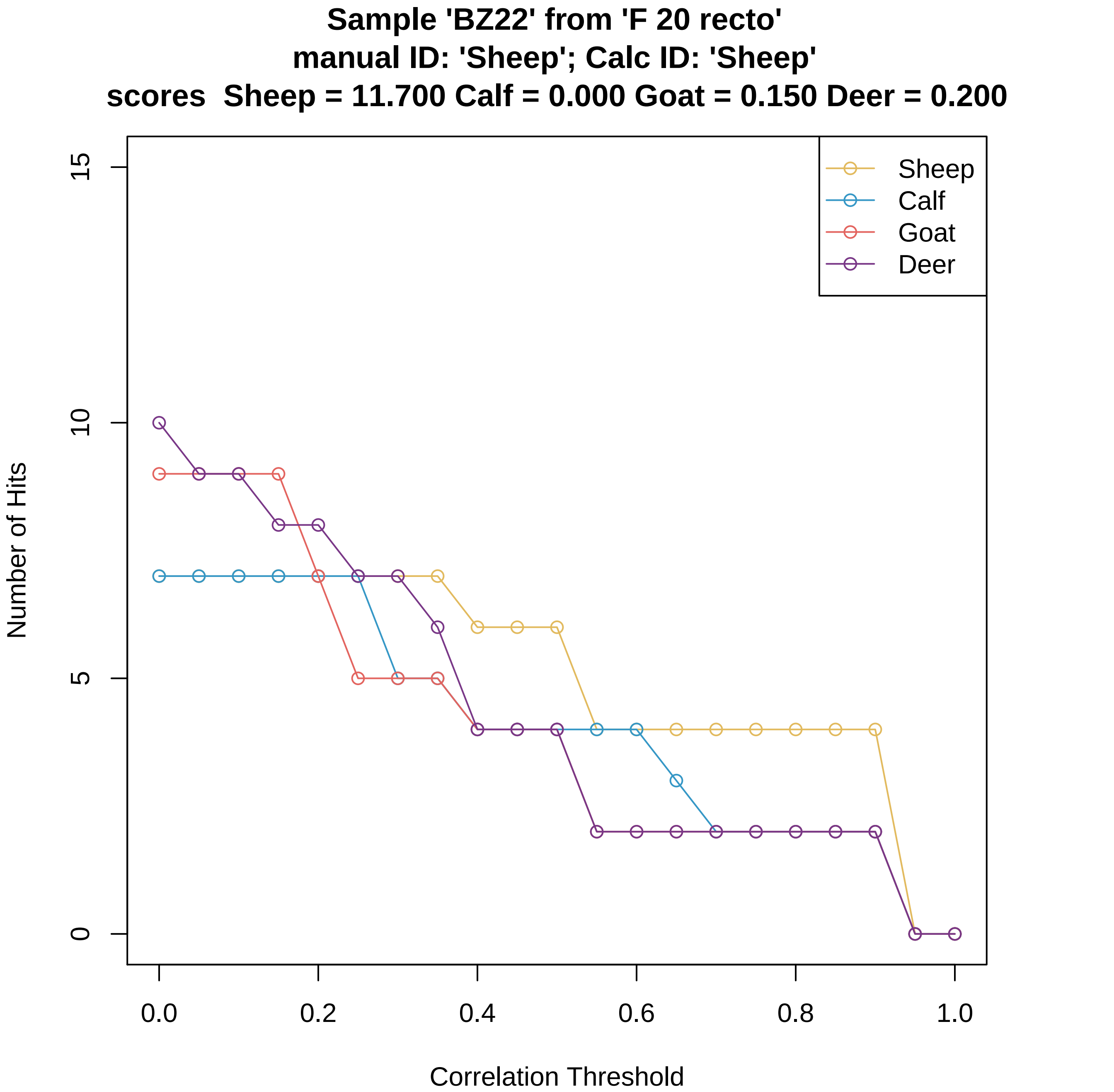
Sample 'BZ22' from 'F 20 recto'
manual ID: 'Sheep'; Calc ID: 'Sheep'
scores Sheep = 11.700 Calf = 0.000 Goat = 0.150 Deer = 0.200
Sheep
Calf
Goat
Deer
Number of Hits
0
5
10
15
Correlation Threshold
0.0
0.2
0.4
0.6
0.8
1.0

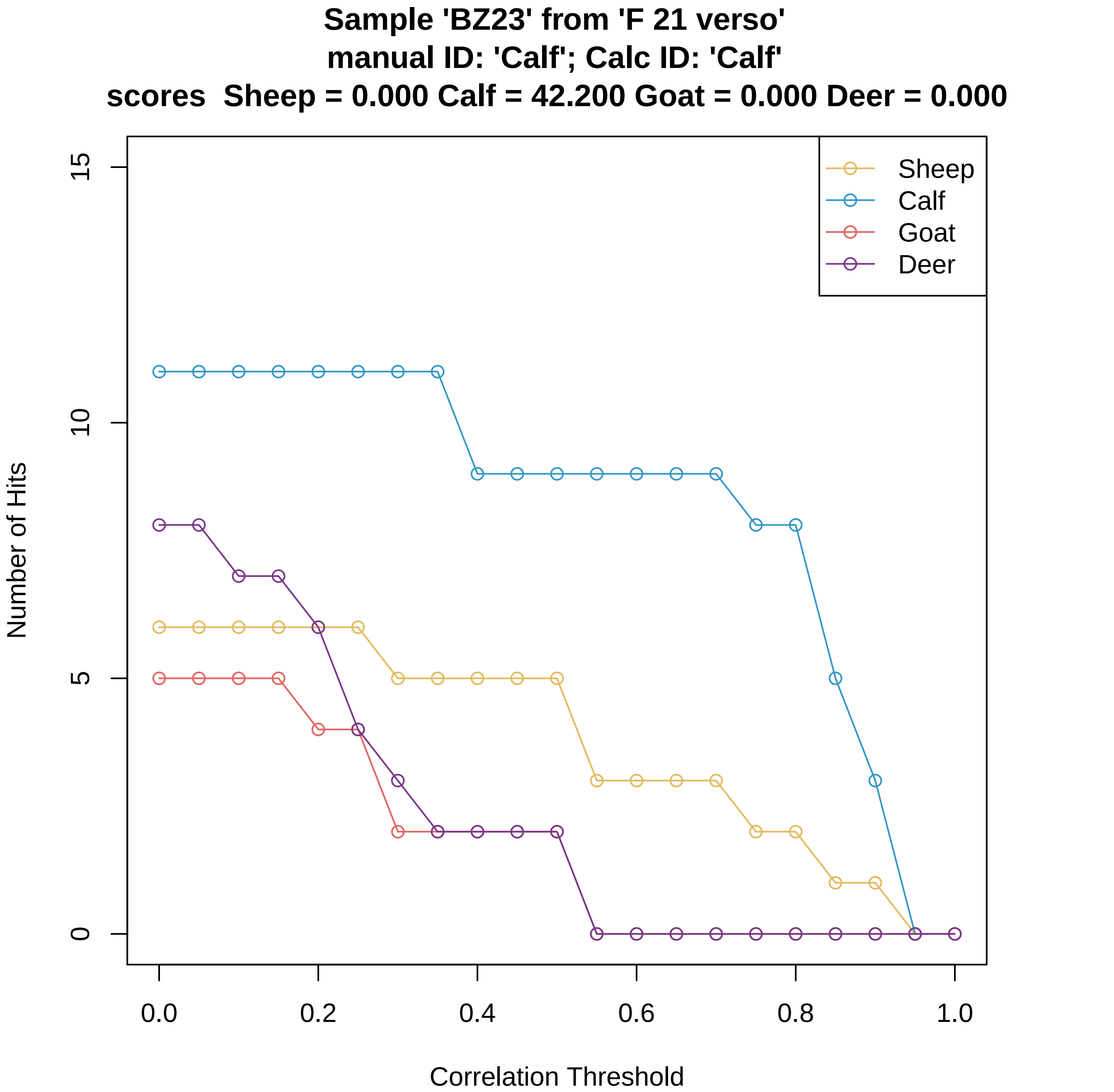

Sample 'BZ23' from 'F 21 verso'
manual ID: 'Calf'; Calc ID: 'Calf'
scores Sheep = 0.000 Calf = 42.200 Goat = 0.000 Deer = 0.000
Sheep
Calf
Goat
Deer
Number of Hits
Correlation Threshold
0
5
10
15
0.0
0.2
0.4
0.6
0.8
1.0

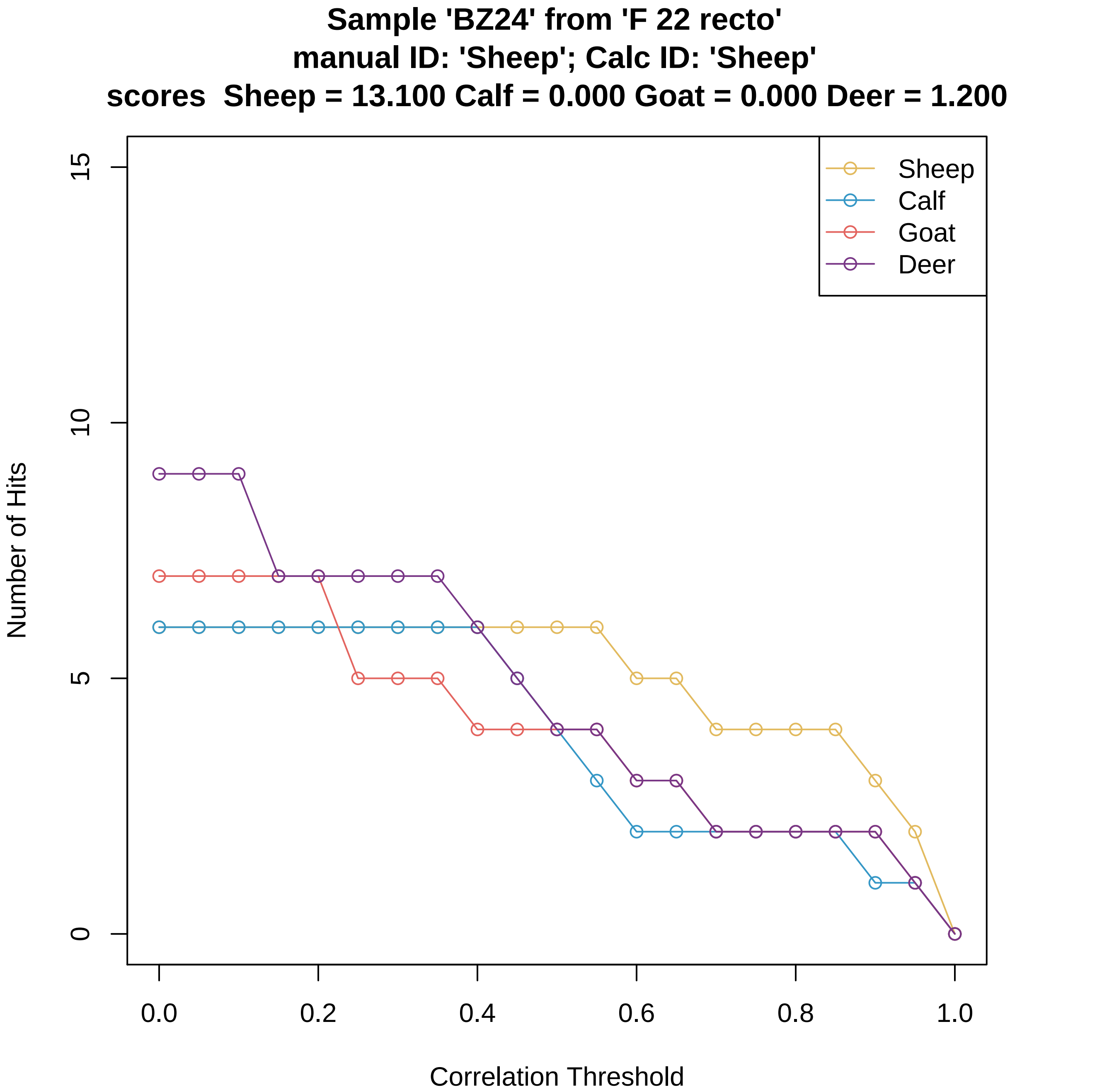

Sample 'BZ24' from 'F 22 recto'
manual ID: 'Sheep'; Calc ID: 'Sheep'
scores Sheep = 13.100 Calf = 0.000 Goat = 0.000 Deer = 1.200
Sheep
Calf
Goat
Deer
Number of Hits
0
5
10
15
0.0
0.2
0.4
0.6
0.8
1.0
Correlation Threshold

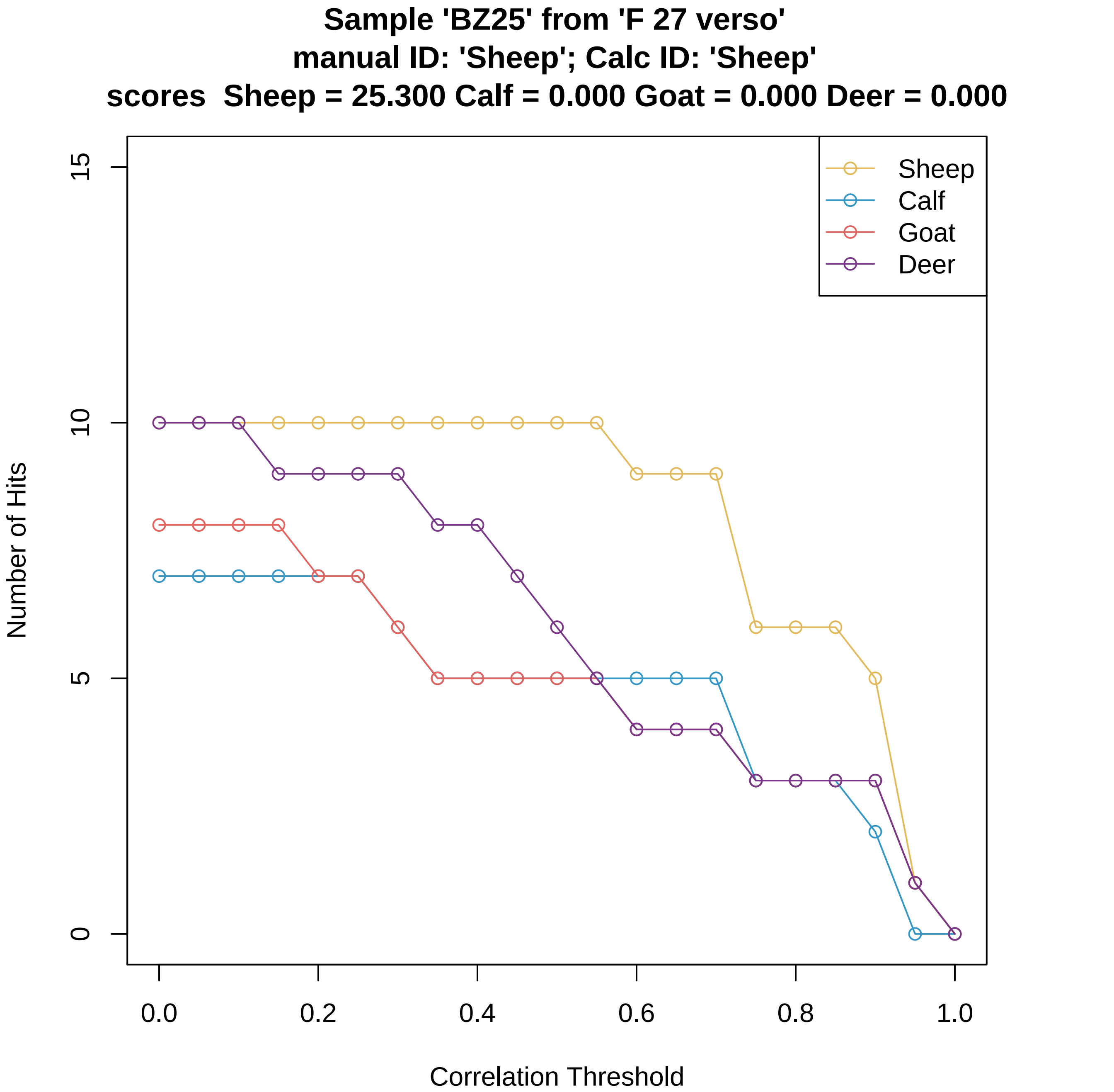
Sample 'BZ25' from 'F 27 verso'
manual ID: 'Sheep'; Calc ID: 'Sheep'
scores Sheep = 25.300 Calf = 0.000 Goat = 0.000 Deer = 0.000
Sheep
Calf
Goat
Deer
Number of Hits
Correlation Threshold
0
5
10
15
0.0
0.2
0.4
0.6
0.8
1.0

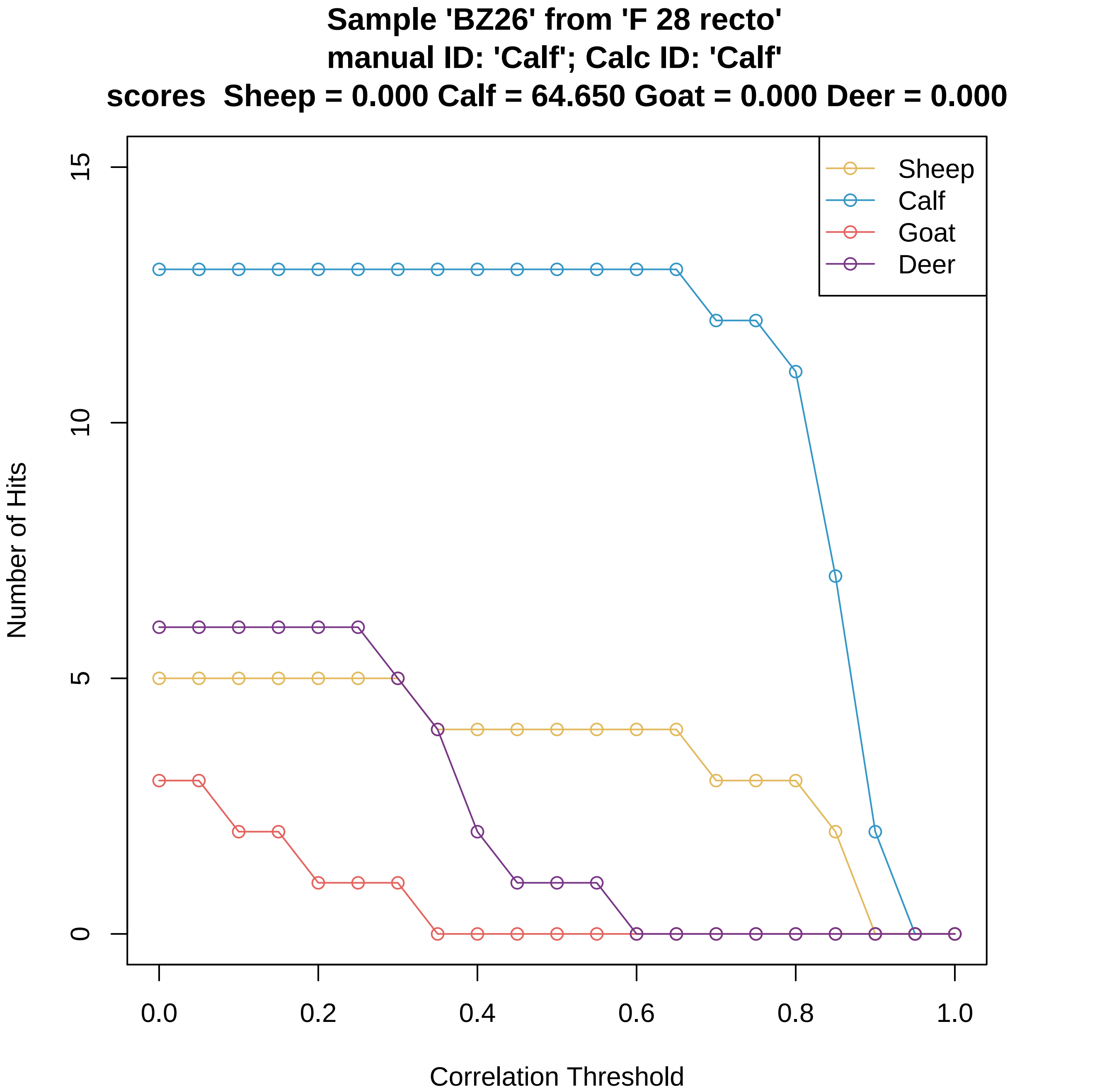

Sample 'BZ26' from 'F 28 recto'
manual ID: 'Calf'; Calc ID: 'Calf'
scores Sheep = 0.000 Calf = 64.650 Goat = 0.000 Deer = 0.000
Sheep
Calf
Goat
Deer
Number of Hits
0
5
10
15
0.0
0.2
0.4
0.6
0.8
1.0
Correlation Threshold

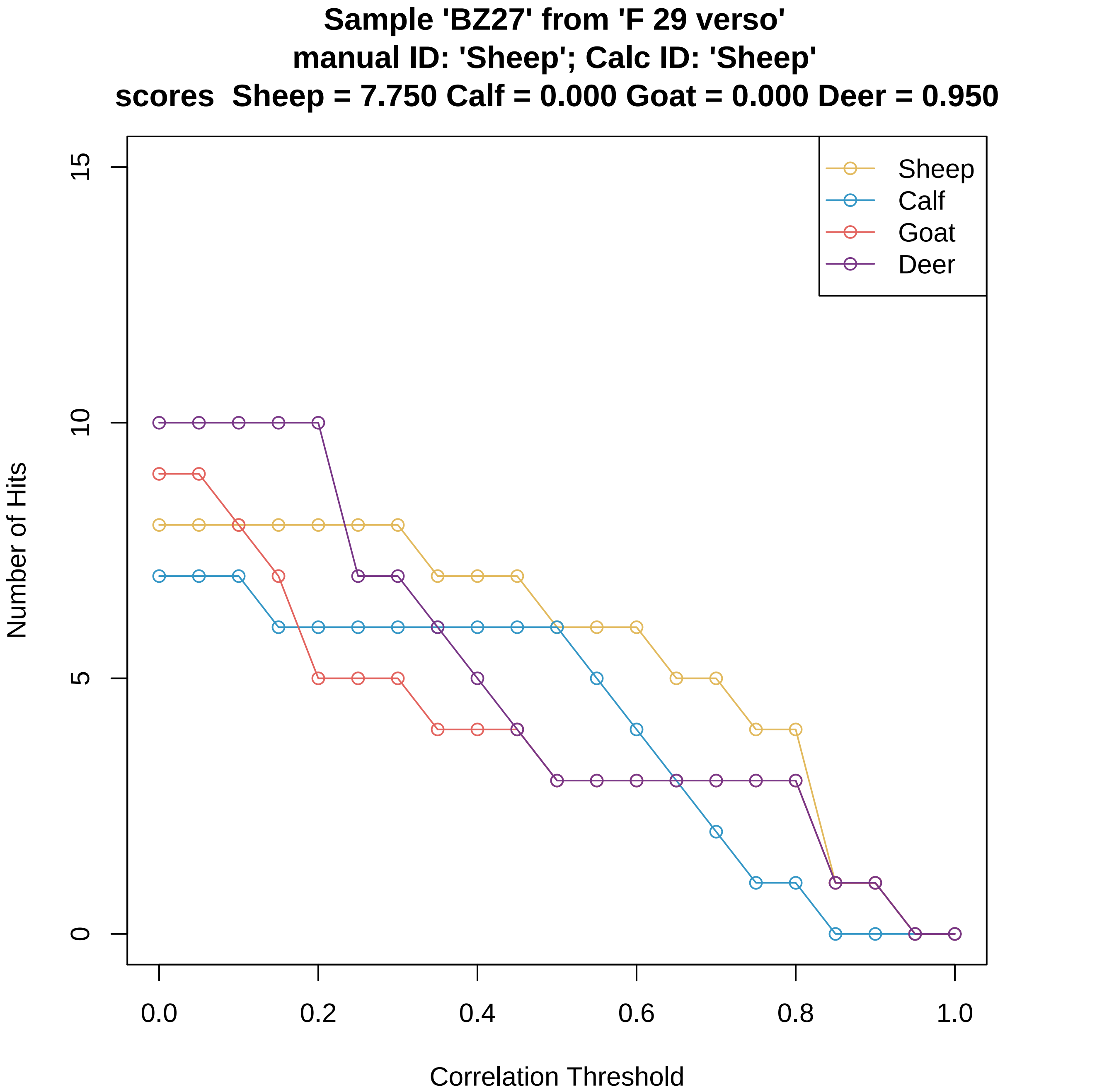

Sample 'BZ27' from 'F 29 verso'
manual ID: 'Sheep'; Calc ID: 'Sheep'
scores Sheep = 7.750 Calf = 0.000 Goat = 0.000 Deer = 0.950
Sheep
Calf
Goat
Deer
Number of Hits
Correlation Threshold
0
5
10
15
0.0
0.2
0.4
0.6
0.8
1.0

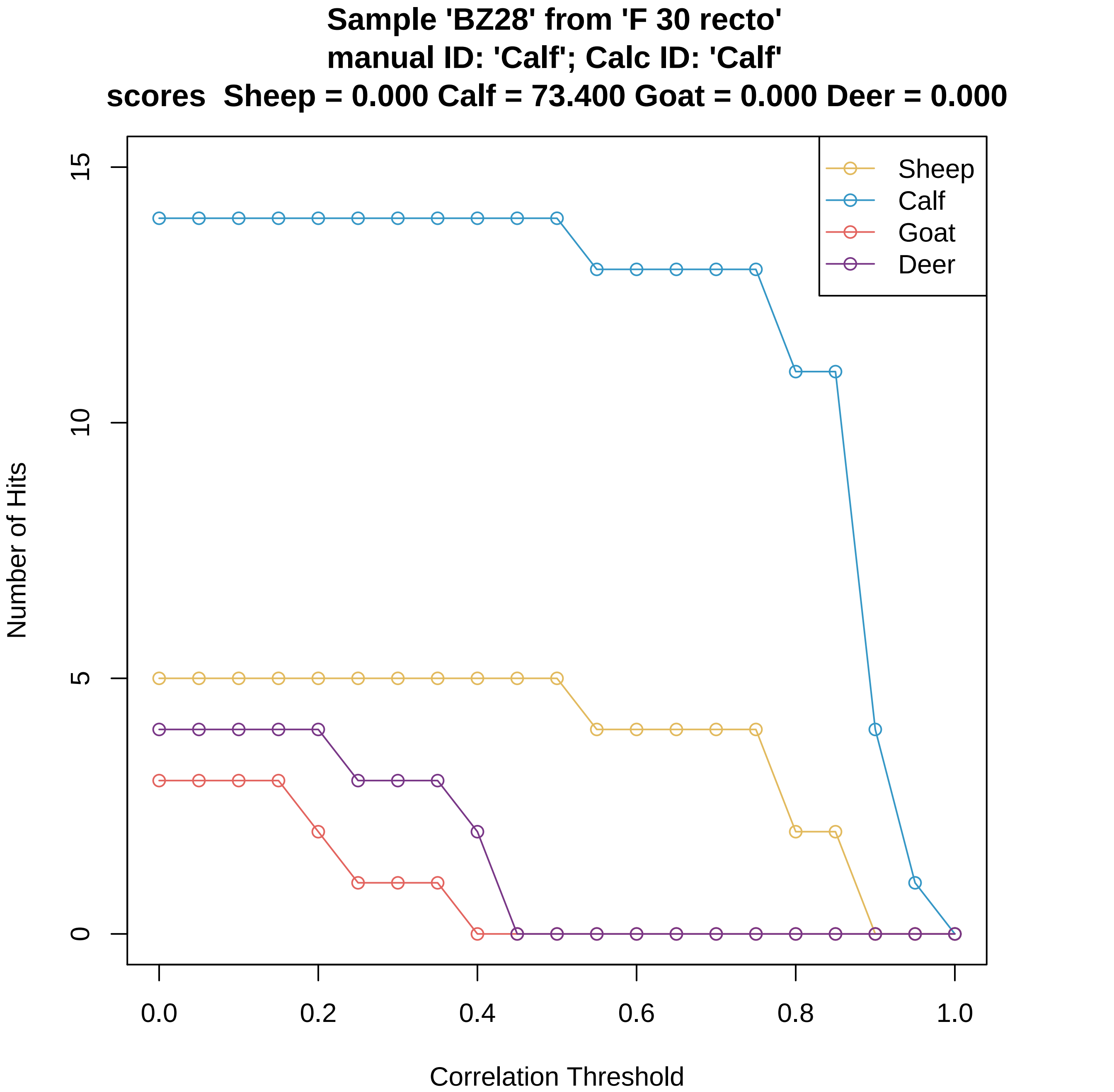

Sample 'BZ28' from 'F 30 recto'
manual ID: 'Calf'; Calc ID: 'Calf'
scores Sheep = 0.000 Calf = 73.400 Goat = 0.000 Deer = 0.000
Sheep
Calf
Goat
Deer
Number of Hits
0
5
10
15
0.0
0.2
0.4
0.6
0.8
1.0
Correlation Threshold

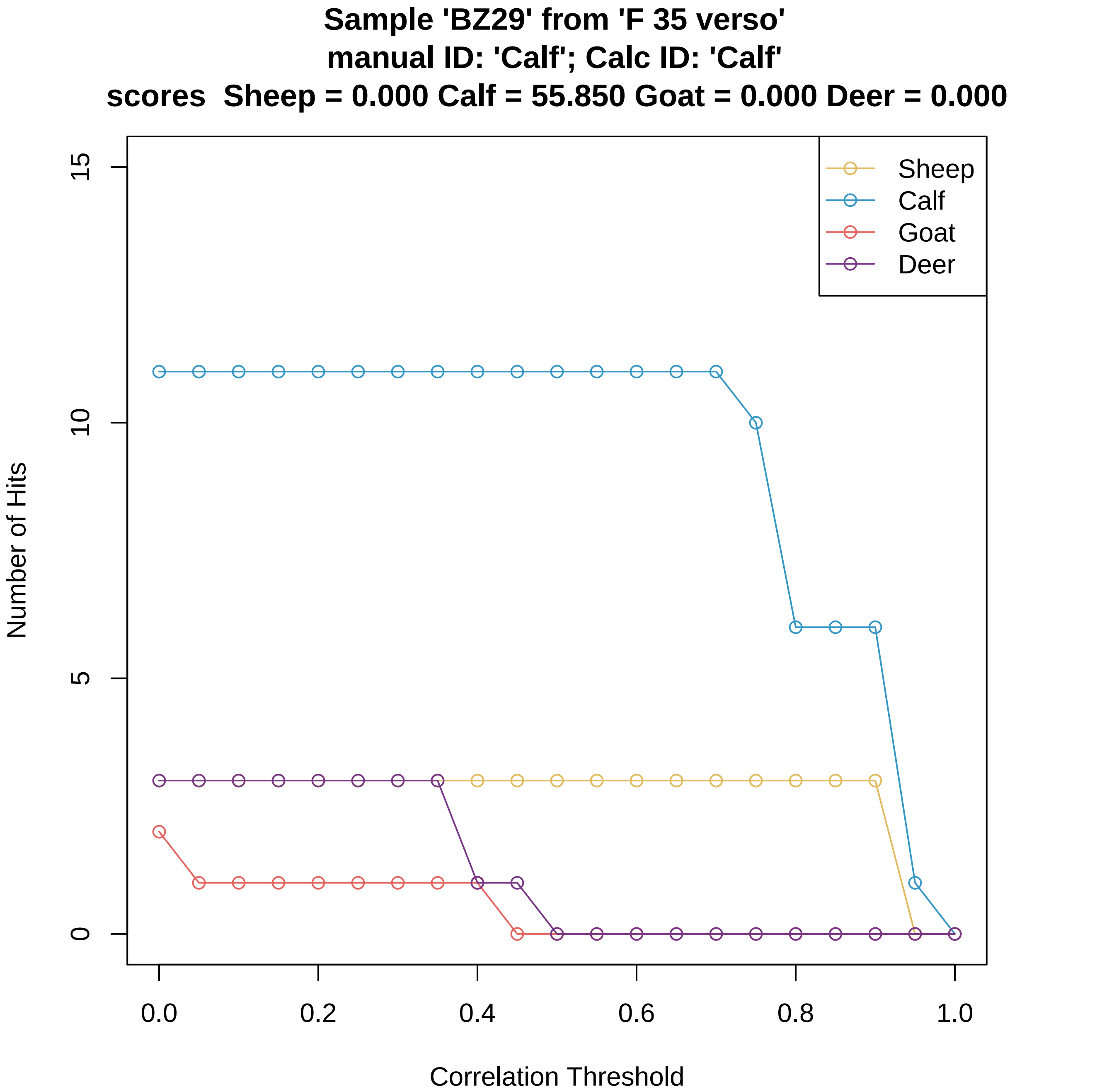

Sample 'BZ29' from 'F 35 verso'
manual ID: 'Calf'; Calc ID: 'Calf'
scores Sheep = 0.000 Calf = 55.850 Goat = 0.000 Deer = 0.000
Sheep
Calf
Goat
Deer
Number of Hits
0
5
10
15
0.0
0.2
0.4
0.6
0.8
1.0
Correlation Threshold

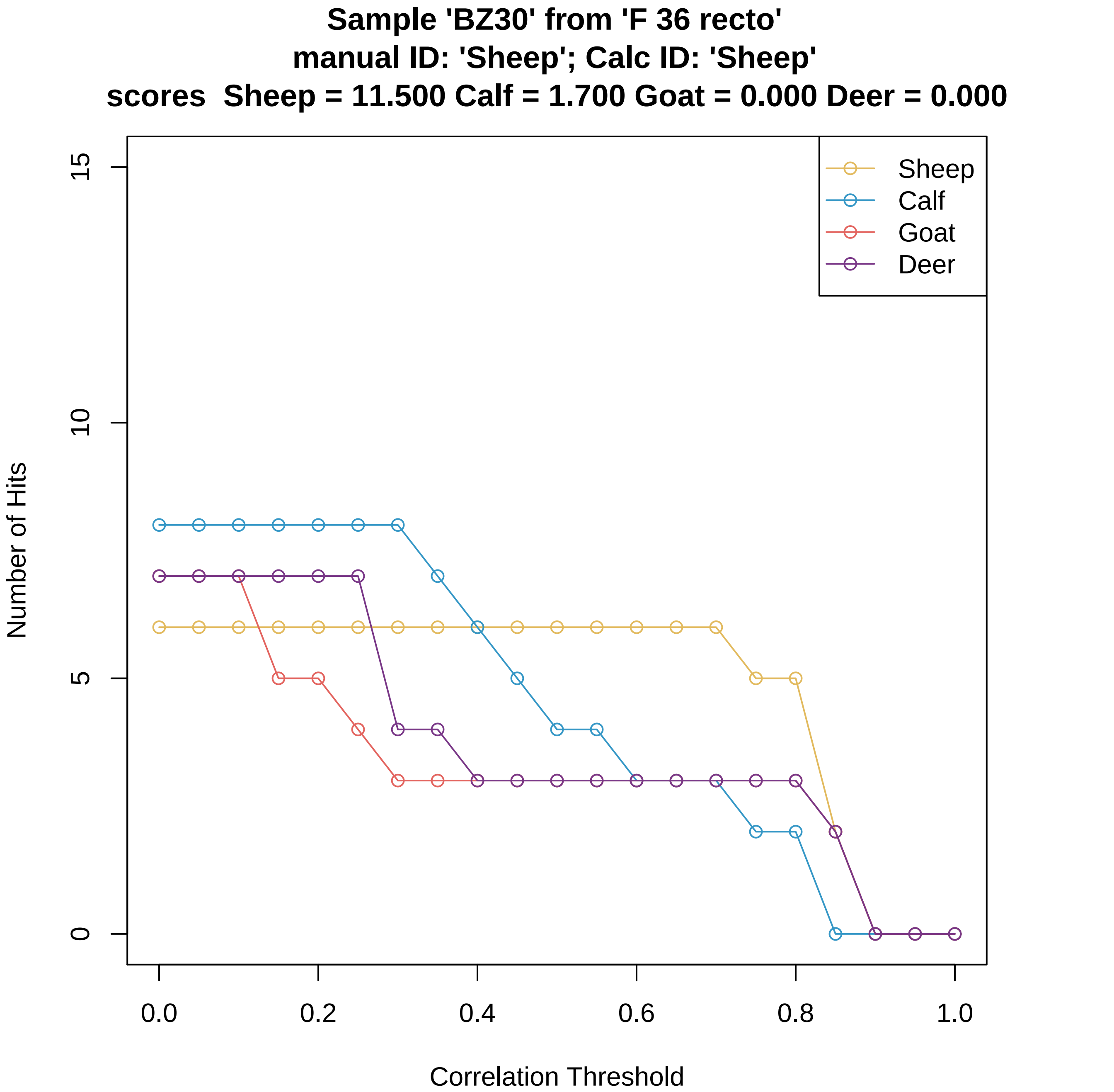

Sample 'BZ30' from 'F 36 recto'
manual ID: 'Sheep'; Calc ID: 'Sheep'
scores Sheep = 11.500 Calf = 1.700 Goat = 0.000 Deer = 0.000
Sheep
Calf
Goat
Deer
Number of Hits
Correlation Threshold
0
5
10
15
0.0
0.2
0.4
0.6
0.8
1.0

Sample 'BZ31' from 'F 37 verso'
manual ID: 'Calf'; Calc ID: 'Calf'
scores Sheep = 0.000 Calf = 41.350 Goat = 0.000 Deer = 0.000

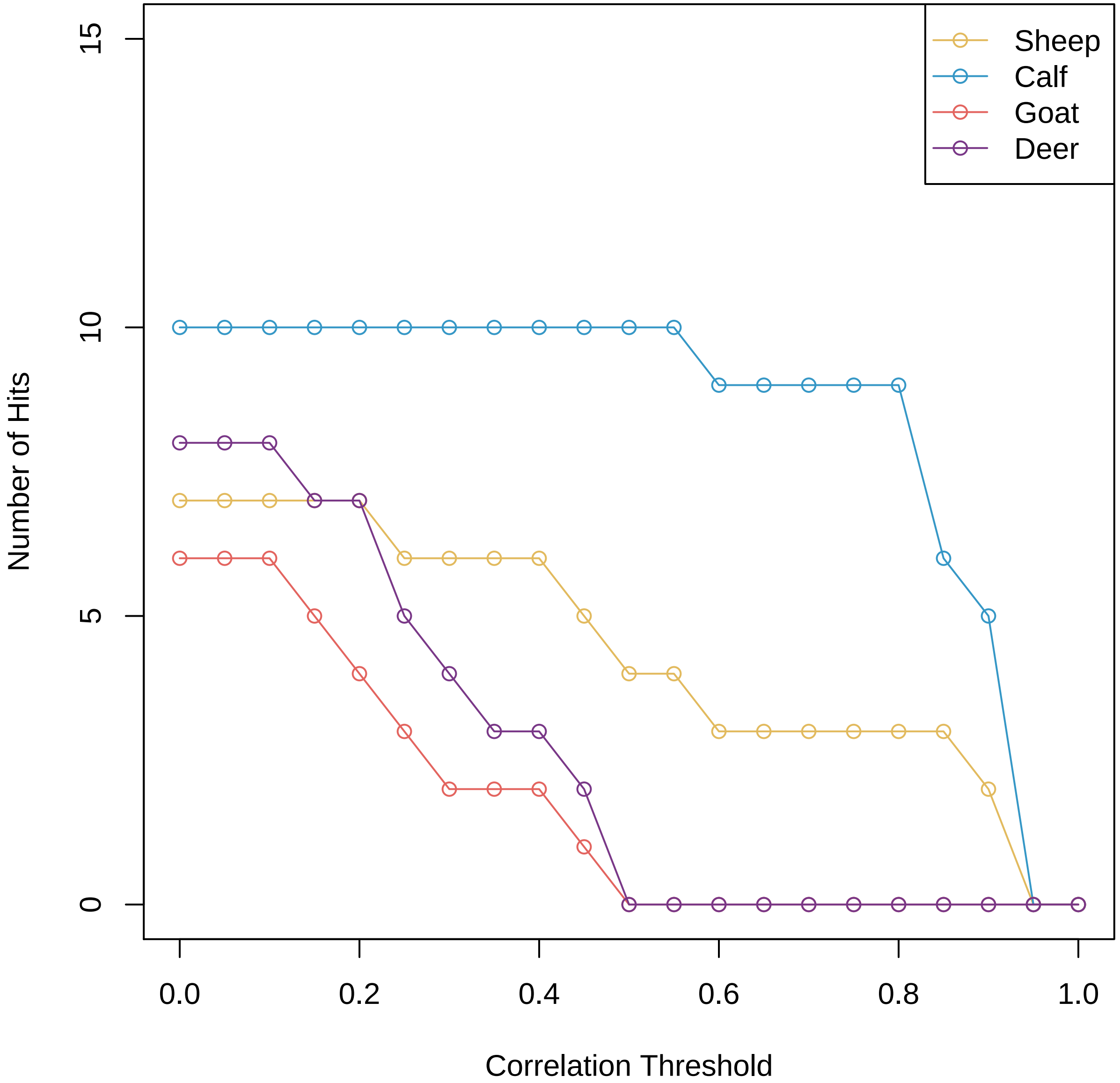

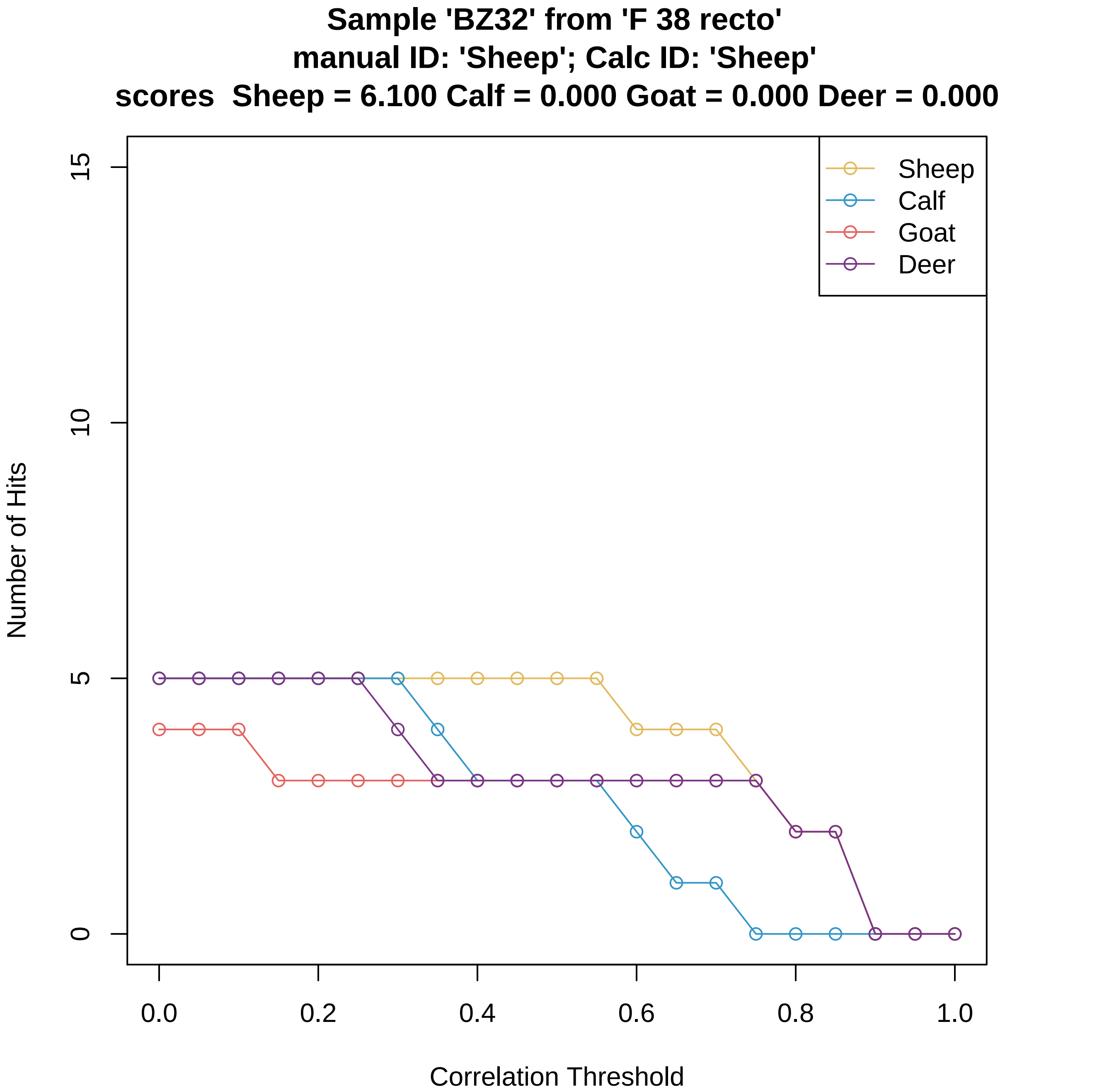

Sample 'BZ32' from 'F 38 recto'
manual ID: 'Sheep'; Calc ID: 'Sheep'
scores Sheep = 6.100 Calf = 0.000 Goat = 0.000 Deer = 0.000
Sheep
Calf
Goat
Deer
Number of Hits
0
5
10
15
0.0
0.2
0.4
0.6
0.8
1.0
Correlation Threshold

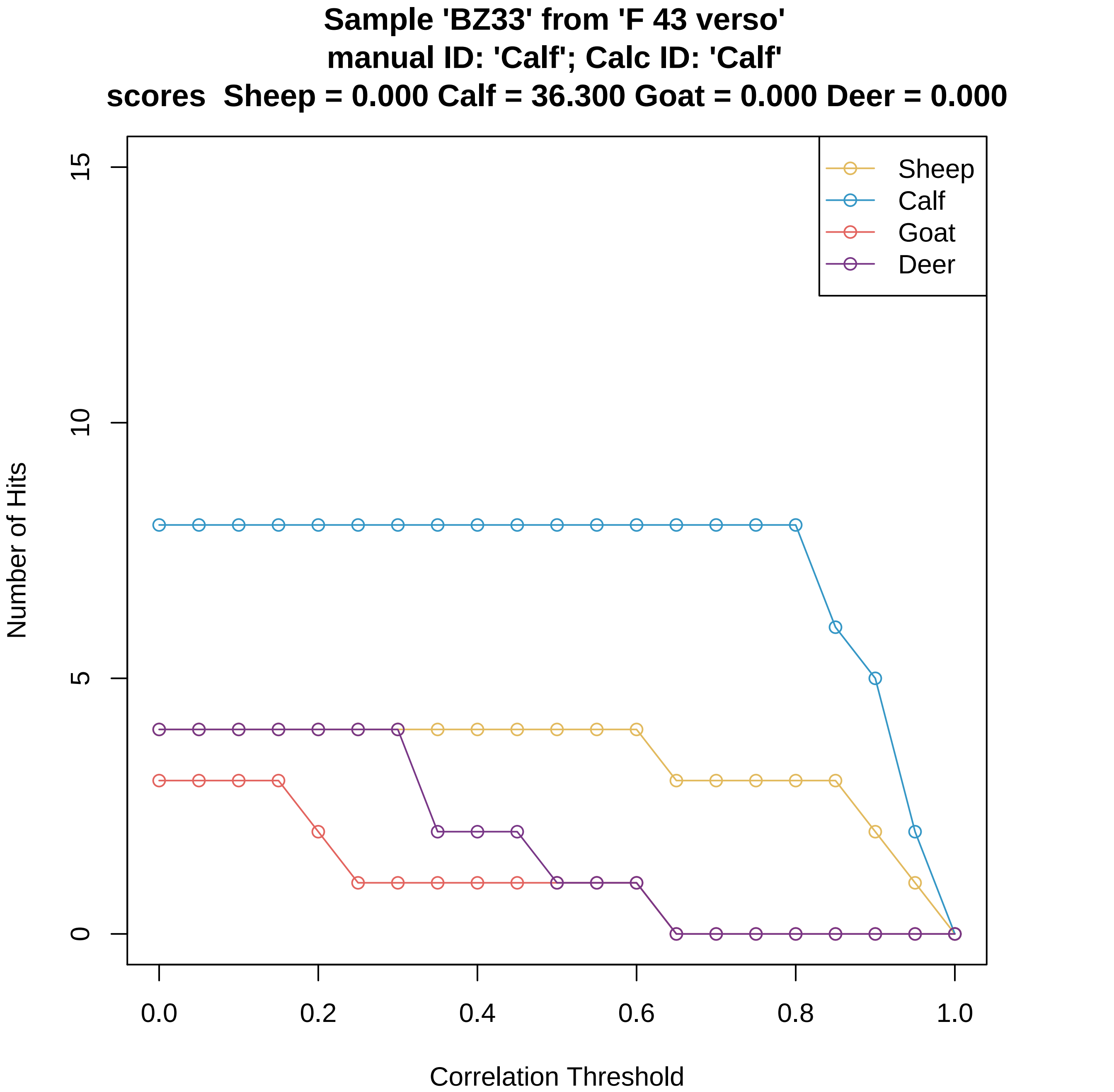
Sample 'BZ33' from 'F 43 verso'
manual ID: 'Calf'; Calc ID: 'Calf'
scores Sheep = 0.000 Calf = 36.300 Goat = 0.000 Deer = 0.000
Number of Hits
Correlation Threshold
0
5
10
15
0.0
0.2
0.4
0.6
0.8
1.0
Sheep
Calf
Goat
Deer

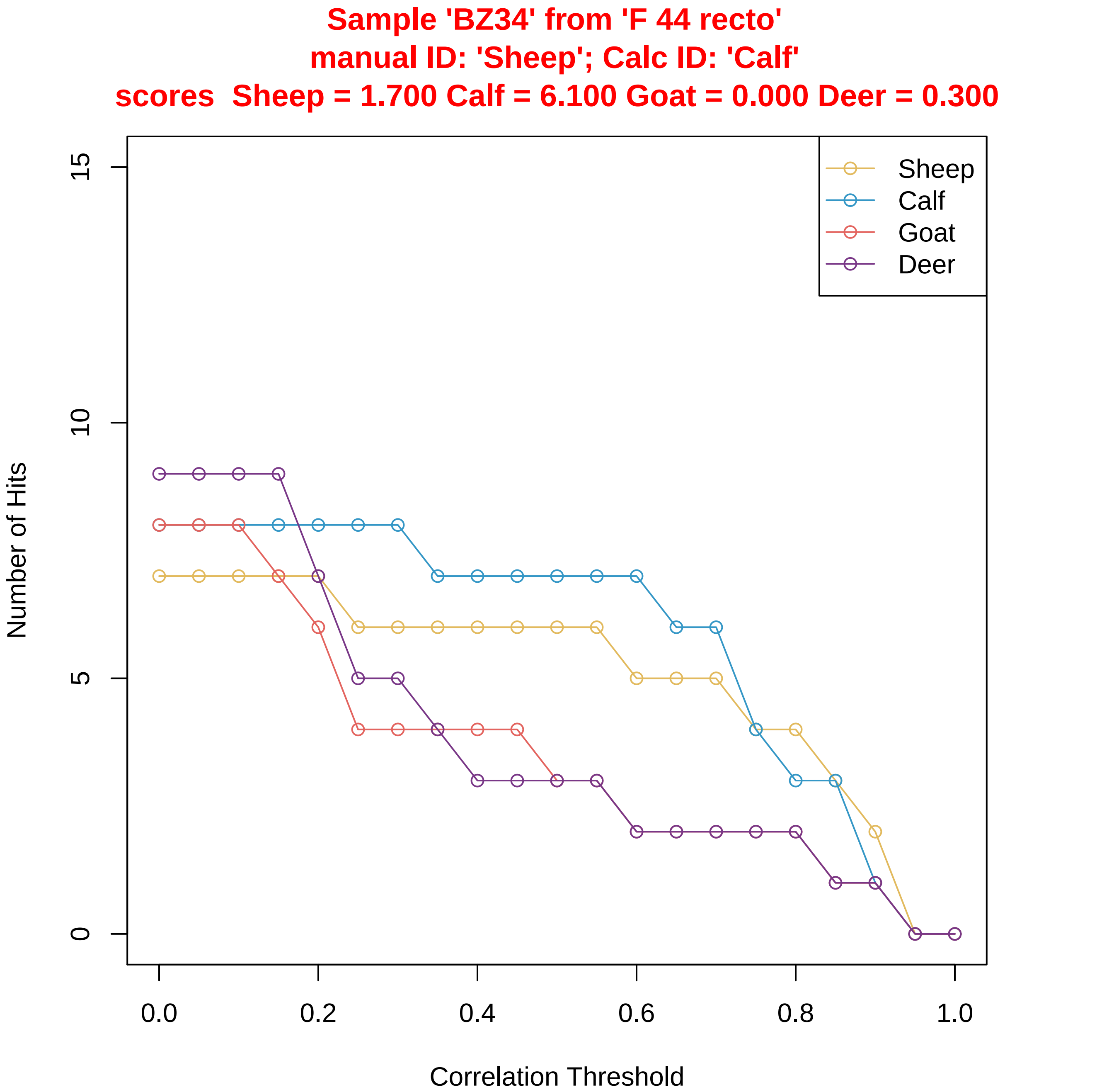

Sample 'BZ34' from 'F 44 recto'
manual ID: 'Sheep'; Calc ID: 'Calf'
scores Sheep = 1.700 Calf = 6.100 Goat = 0.000 Deer = 0.300
Sheep
Calf
Goat
Deer
Number of Hits
0
5
10
15
0.0
0.2
0.4
0.6
0.8
1.0
Correlation Threshold

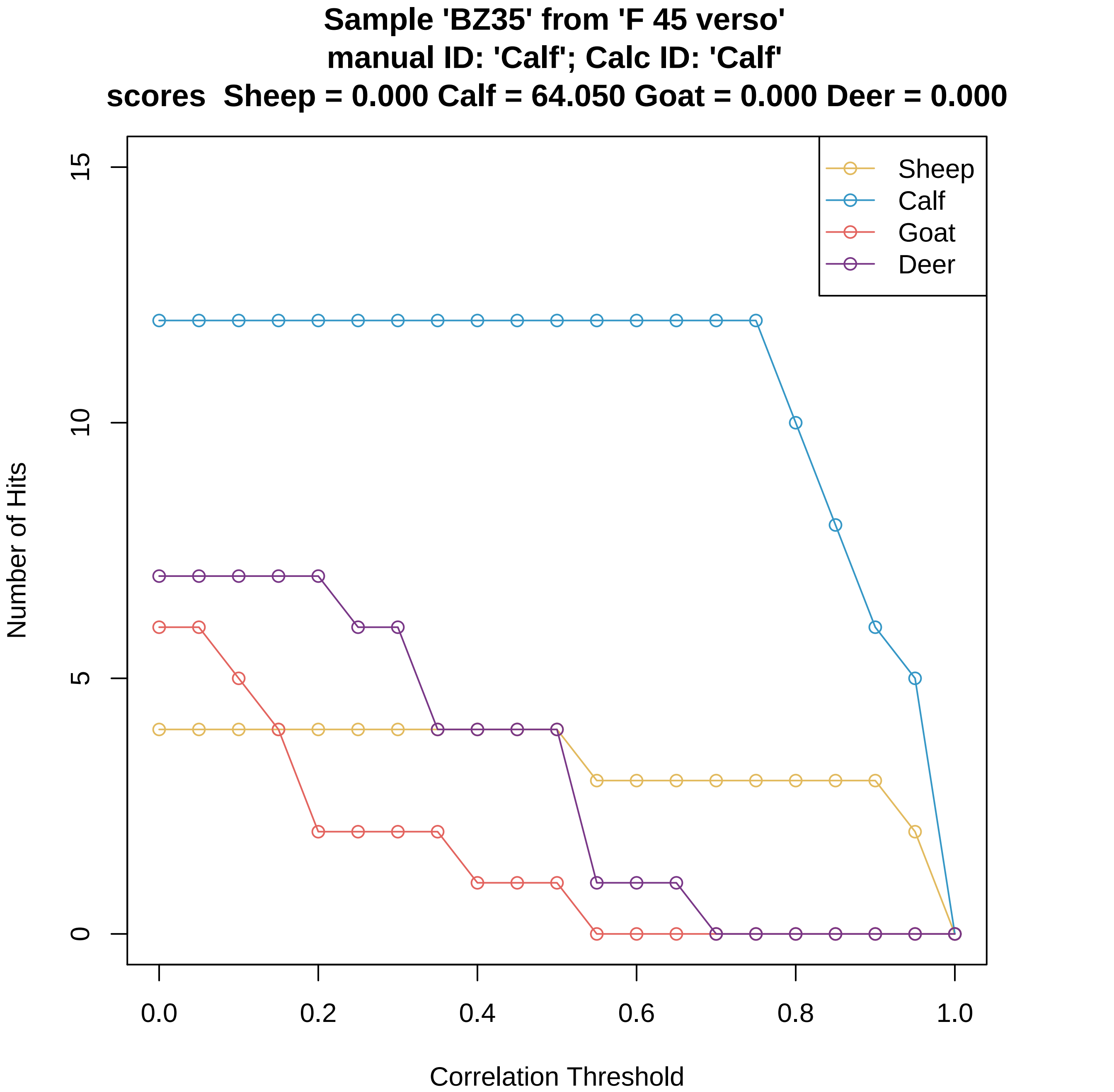

Sample 'BZ35' from 'F 45 verso'
manual ID: 'Calf'; Calc ID: 'Calf'
scores Sheep = 0.000 Calf = 64.050 Goat = 0.000 Deer = 0.000
Sheep
Calf
Goat
Deer
0
5
10
15
0.0
0.2
0.4
0.6
0.8
1.0
Number of Hits
Correlation Threshold

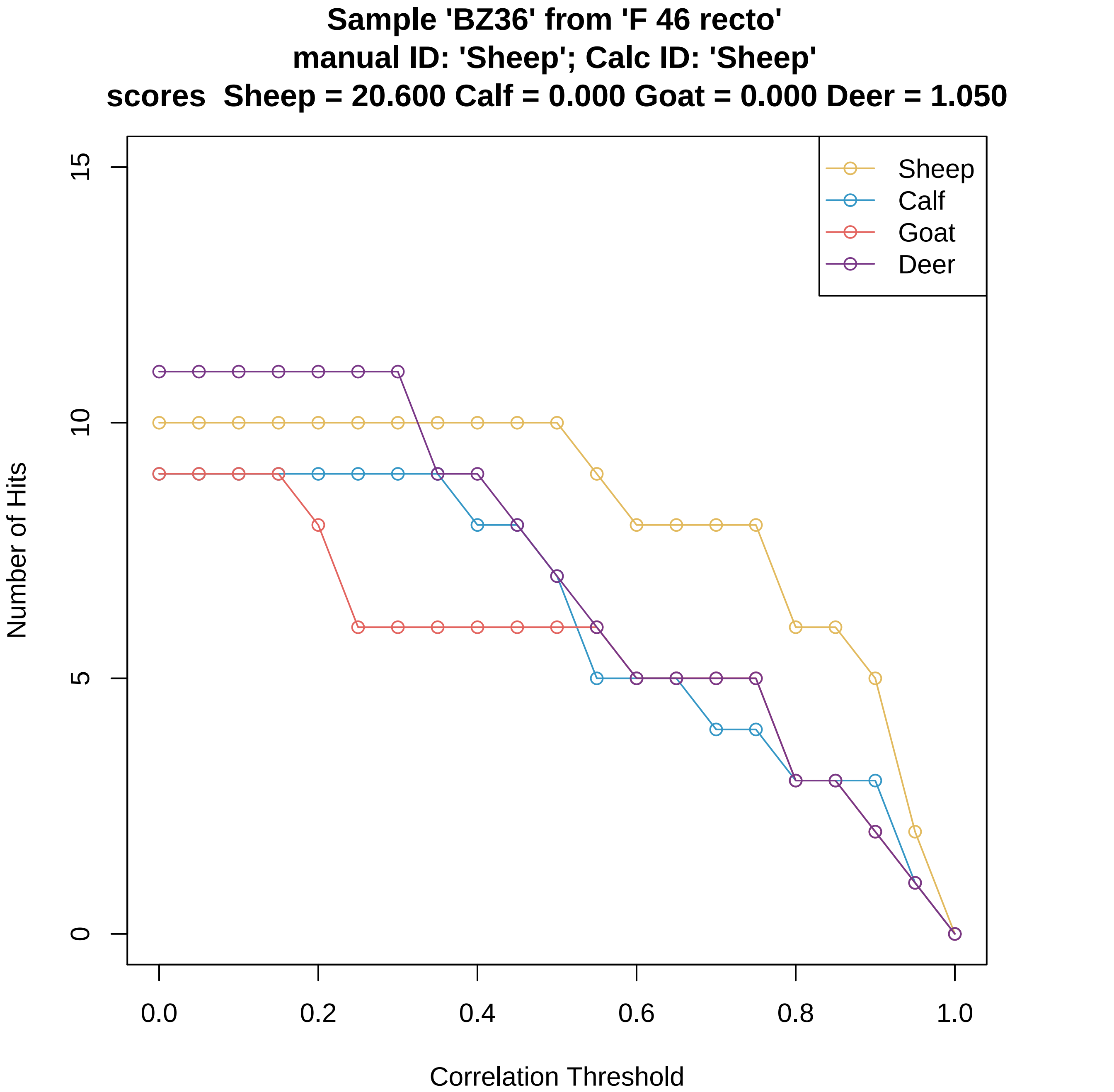

Sample 'BZ36' from 'F 46 recto'
manual ID: 'Sheep'; Calc ID: 'Sheep'
scores Sheep = 20.600 Calf = 0.000 Goat = 0.000 Deer = 1.050
Sheep
Calf
Goat
Deer
Number of Hits
Correlation Threshold
0
5
10
15
0.0
0.2
0.4
0.6
0.8
1.0

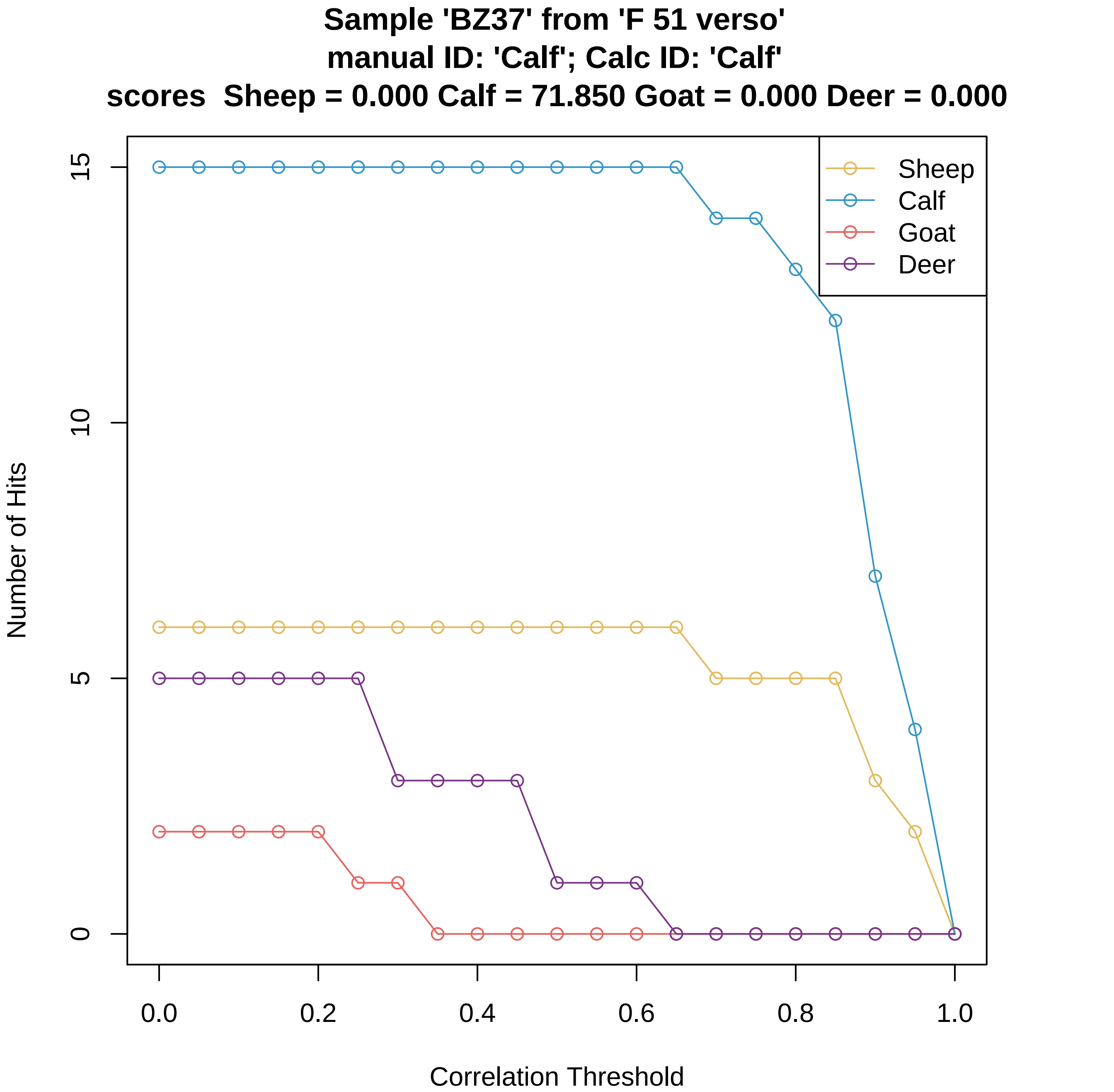
Sample 'BZ37' from 'F 51 verso'
manual ID: 'Calf'; Calc ID: 'Calf'
scores Sheep = 0.000 Calf = 71.850 Goat = 0.000 Deer = 0.000
Sheep
Calf
Goat
Deer
Number of Hits
0
5
10
15
0.0
0.2
0.4
0.6
0.8
1.0
Correlation Threshold

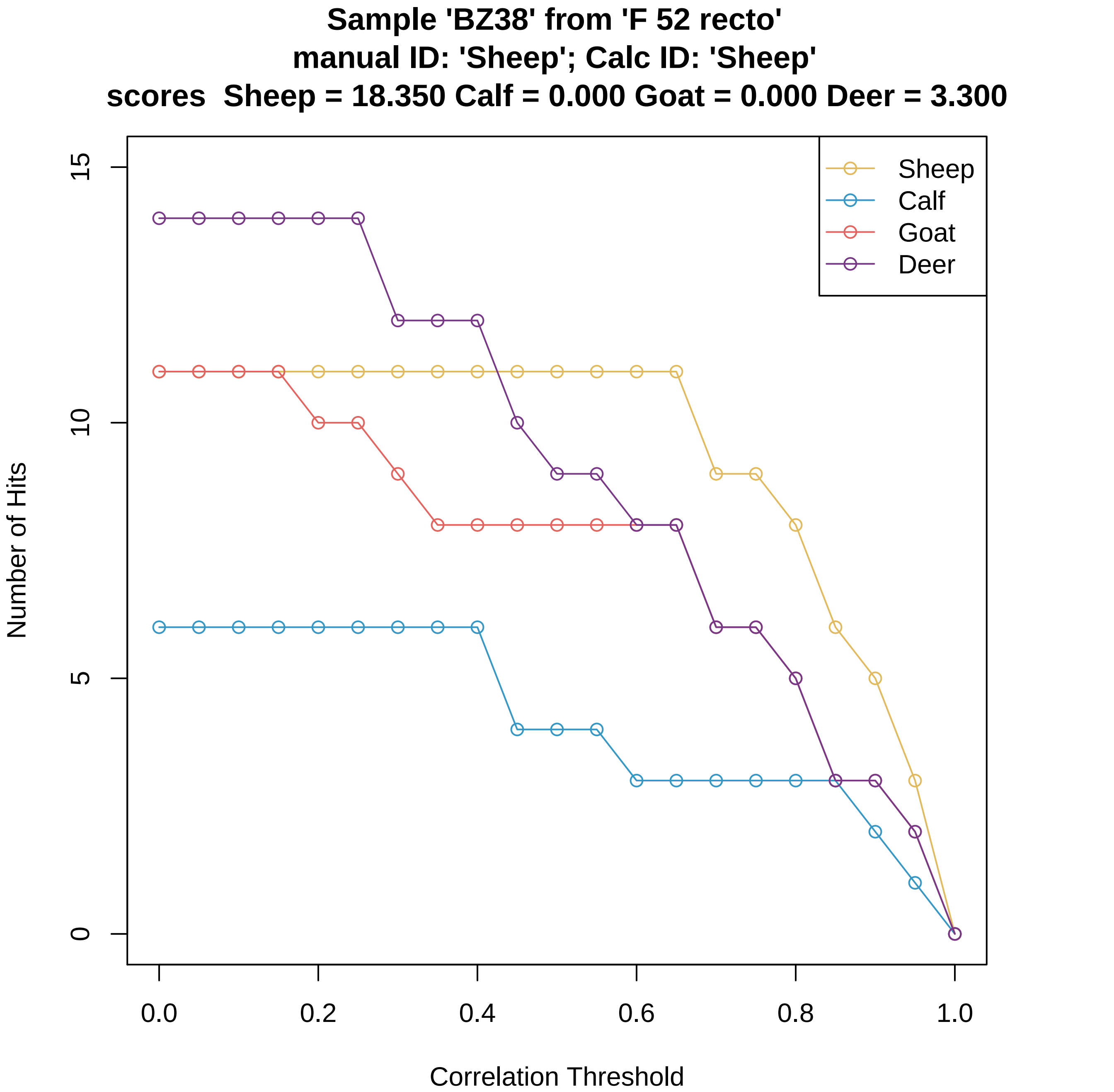

Sample 'BZ38' from 'F 52 recto'
manual ID: 'Sheep'; Calc ID: 'Sheep'
scores Sheep = 18.350 Calf = 0.000 Goat = 0.000 Deer = 3.300
Sheep
Calf
Goat
Deer
Number of Hits
0
5
10
15
0.0
0.2
0.4
0.6
0.8
1.0
Correlation Threshold

Sample 'BZ39' from 'F 53 verso'
manual ID: 'Calf'; Calc ID: 'Calf'
scores Sheep = 0.000 Calf = 69.500 Goat = 0.000 Deer = 0.000

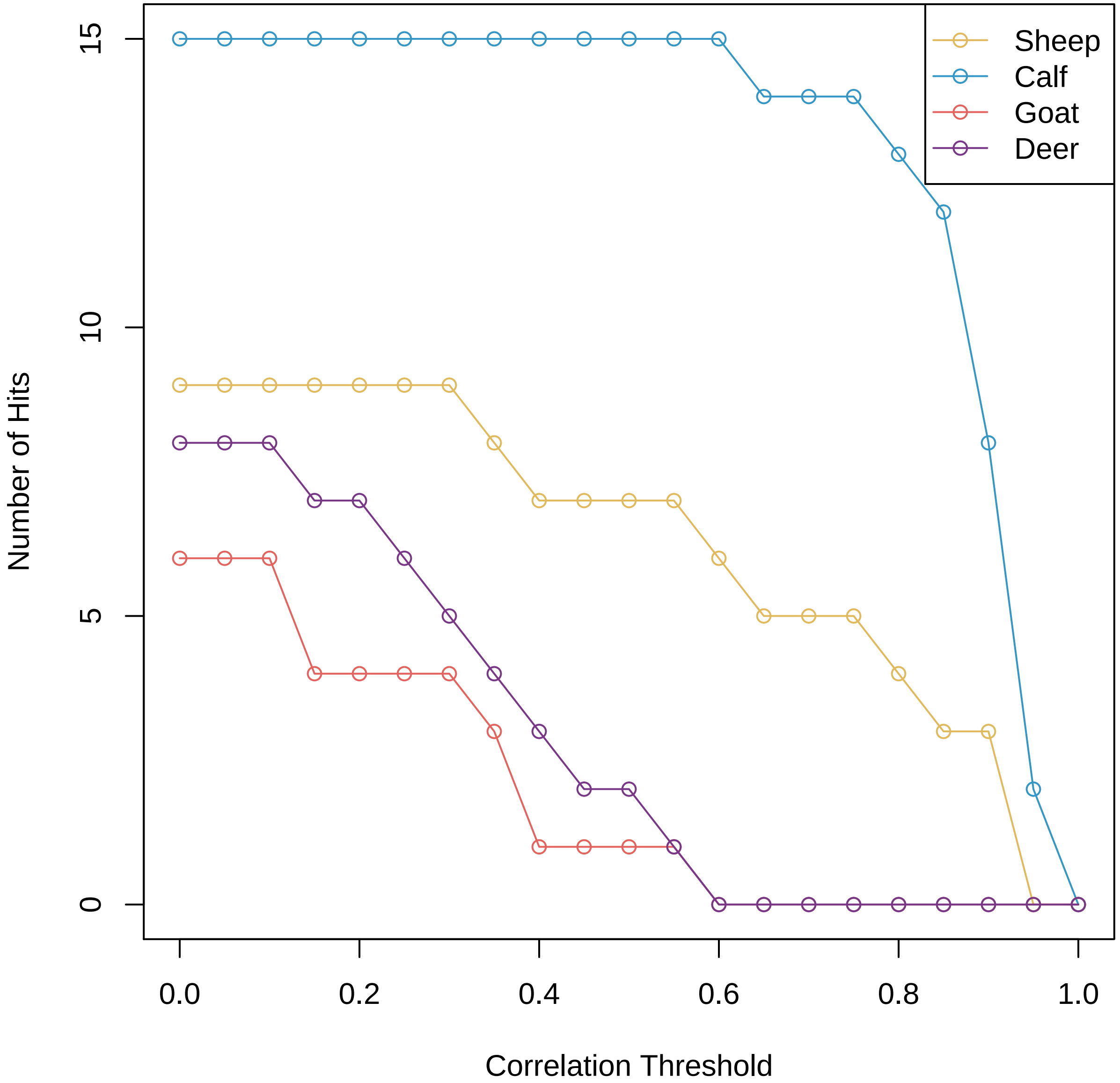

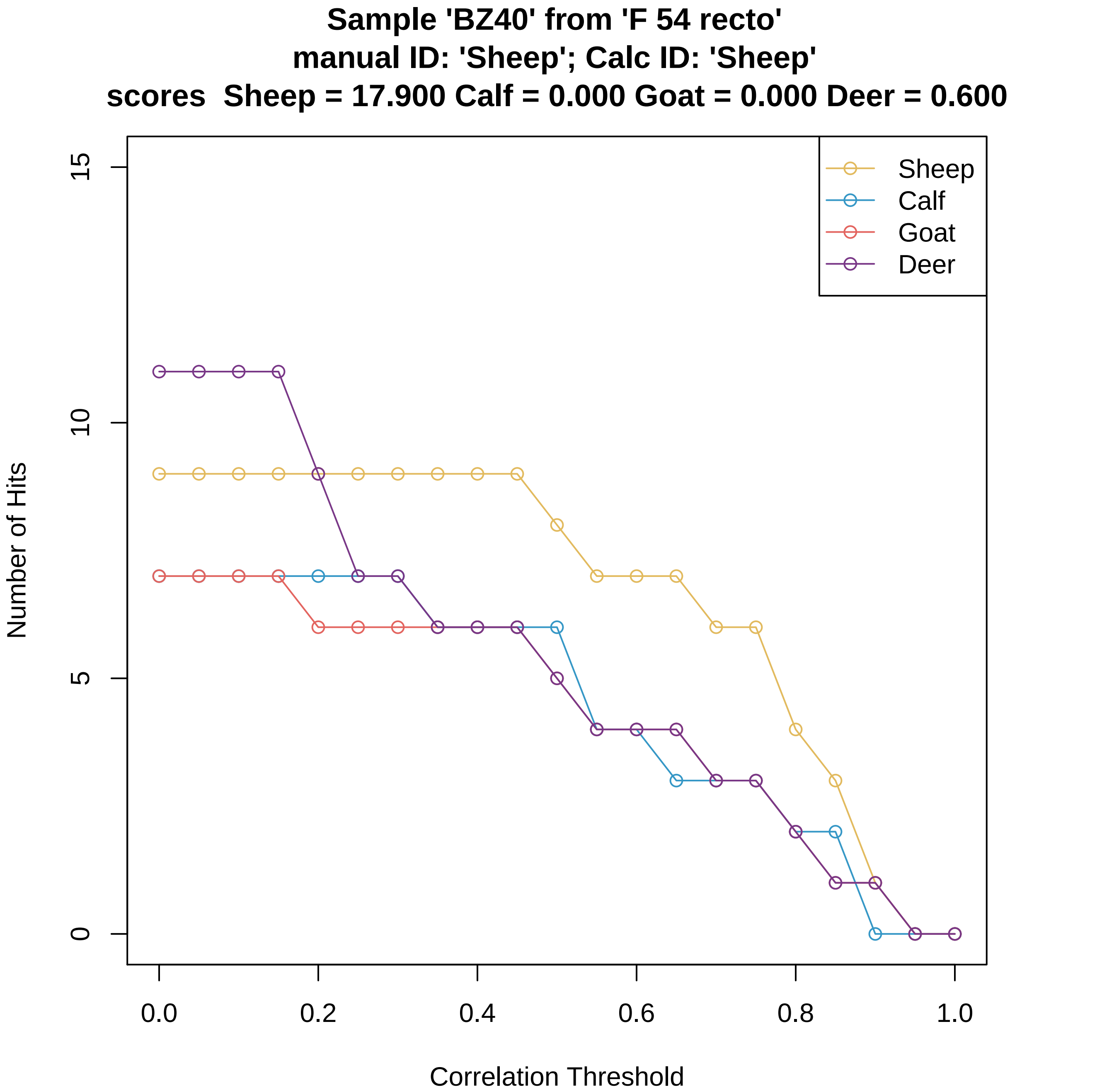
Sample 'BZ40' from 'F 54 recto'
manual ID: 'Sheep'; Calc ID: 'Sheep'
scores Sheep = 17.900 Calf = 0.000 Goat = 0.000 Deer = 0.600
Sheep
Calf
Goat
Deer
Number of Hits
0
5
10
15
0.0
0.2
0.4
0.6
0.8
1.0
Correlation Threshold

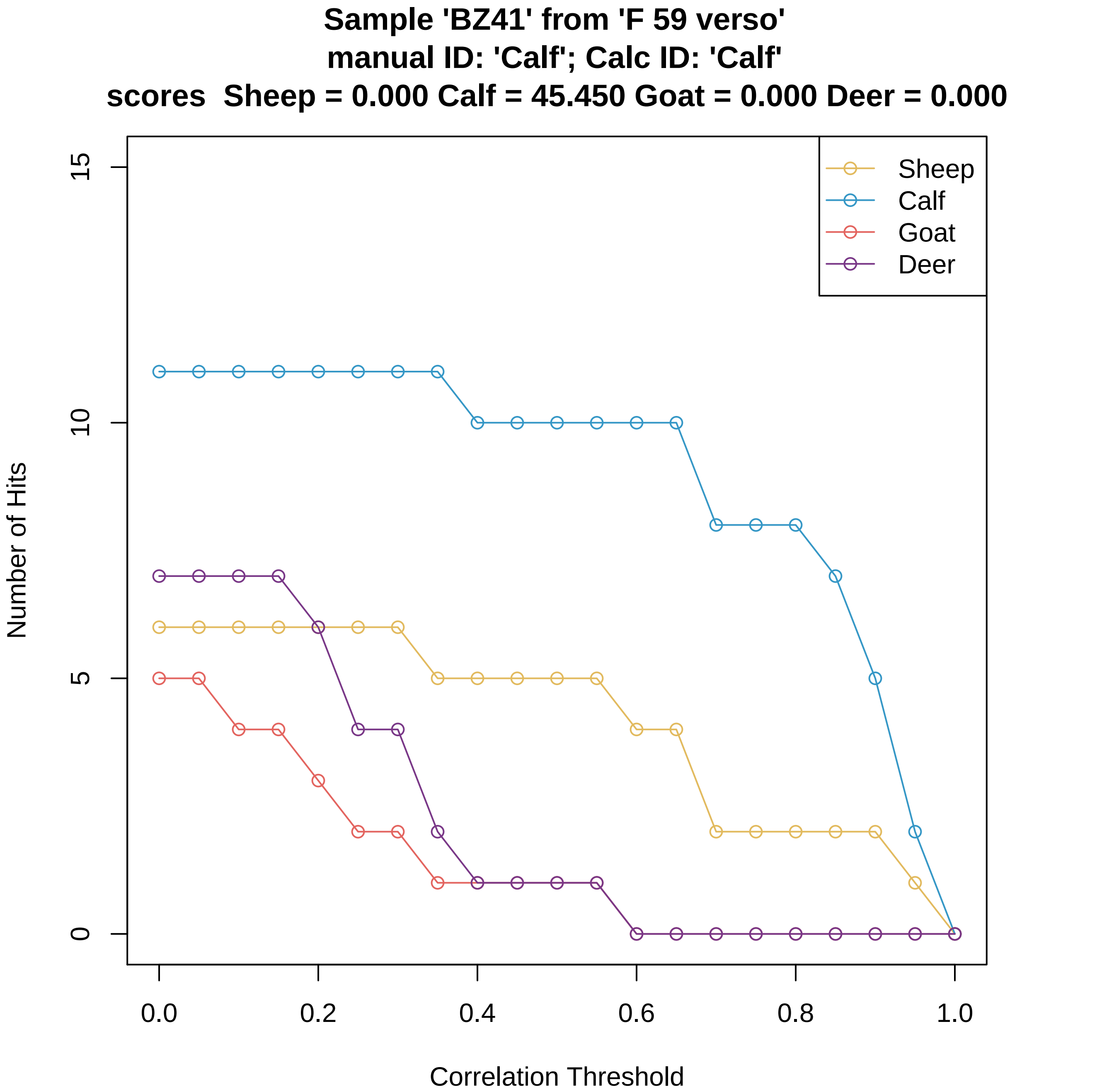

Sample 'BZ41' from 'F 59 verso'
manual ID: 'Calf'; Calc ID: 'Calf'
scores Sheep = 0.000 Calf = 45.450 Goat = 0.000 Deer = 0.000
Sheep
Calf
Goat
Deer
Number of Hits
0
5
10
15
0.0
0.2
0.4
0.6
0.8
1.0
Correlation Threshold

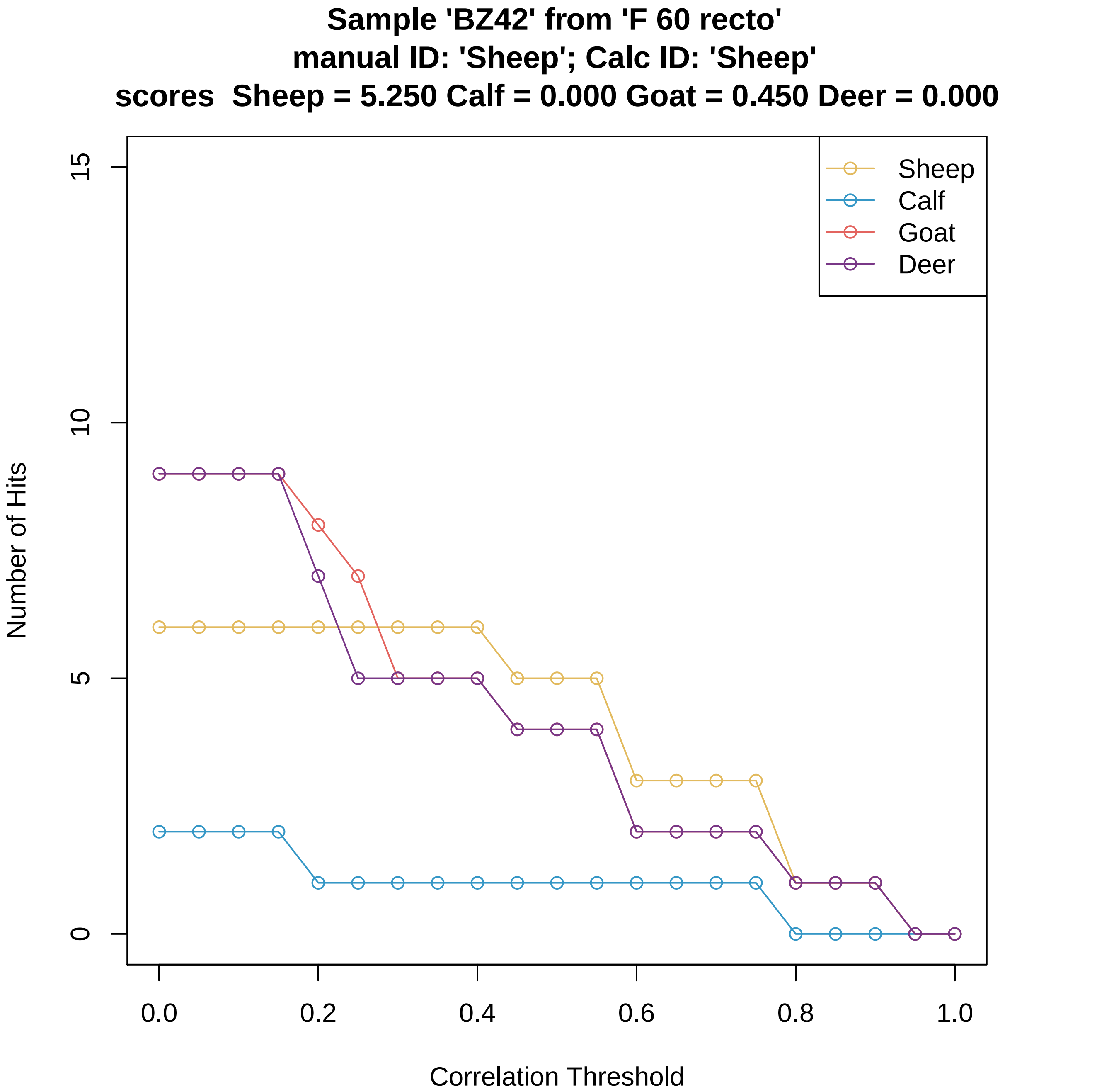
Sample 'BZ42' from 'F 60 recto'
manual ID: 'Sheep'; Calc ID: 'Sheep'
scores Sheep = 5.250 Calf = 0.000 Goat = 0.450 Deer = 0.000
Sheep
Calf
Goat
Deer
Number of Hits
0
5
10
15
0.0
0.2
0.4
0.6
0.8
1.0
Correlation Threshold

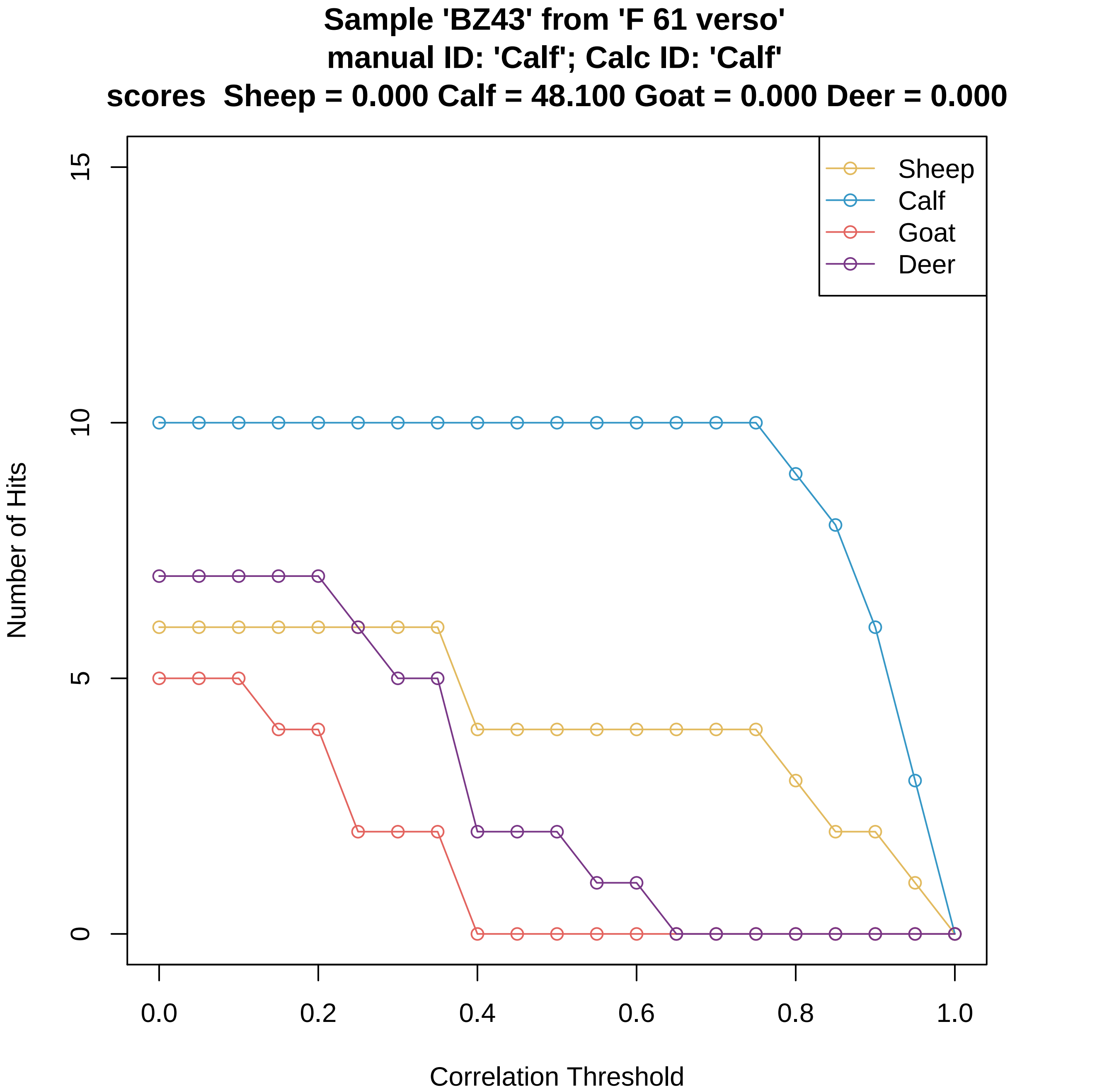

Sample 'BZ43' from 'F 61 verso'
manual ID: 'Calf'; Calc ID: 'Calf'
scores Sheep = 0.000 Calf = 48.100 Goat = 0.000 Deer = 0.000
Sheep
Calf
Goat
Deer
Number of Hits
0
5
10
15
0.0
0.2
0.4
0.6
0.8
1.0
Correlation Threshold

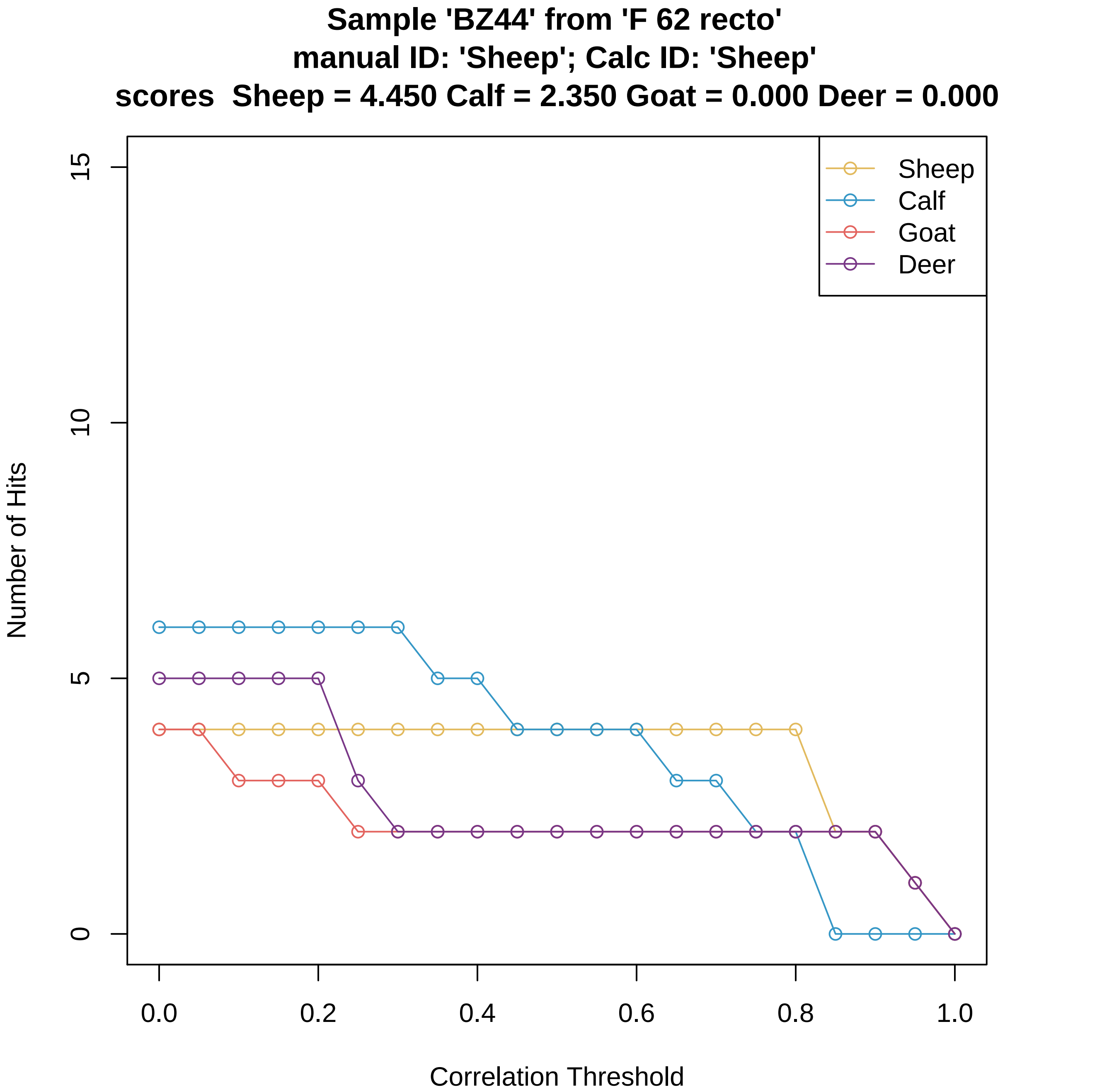

Sample 'BZ44' from 'F 62 recto'
manual ID: 'Sheep'; Calc ID: 'Sheep'
scores Sheep = 4.450 Calf = 2.350 Goat = 0.000 Deer = 0.000
Sheep
Calf
Goat
Deer
Number of Hits
Correlation Threshold
0
5
10
15
0.0
0.2
0.4
0.6
0.8
1.0

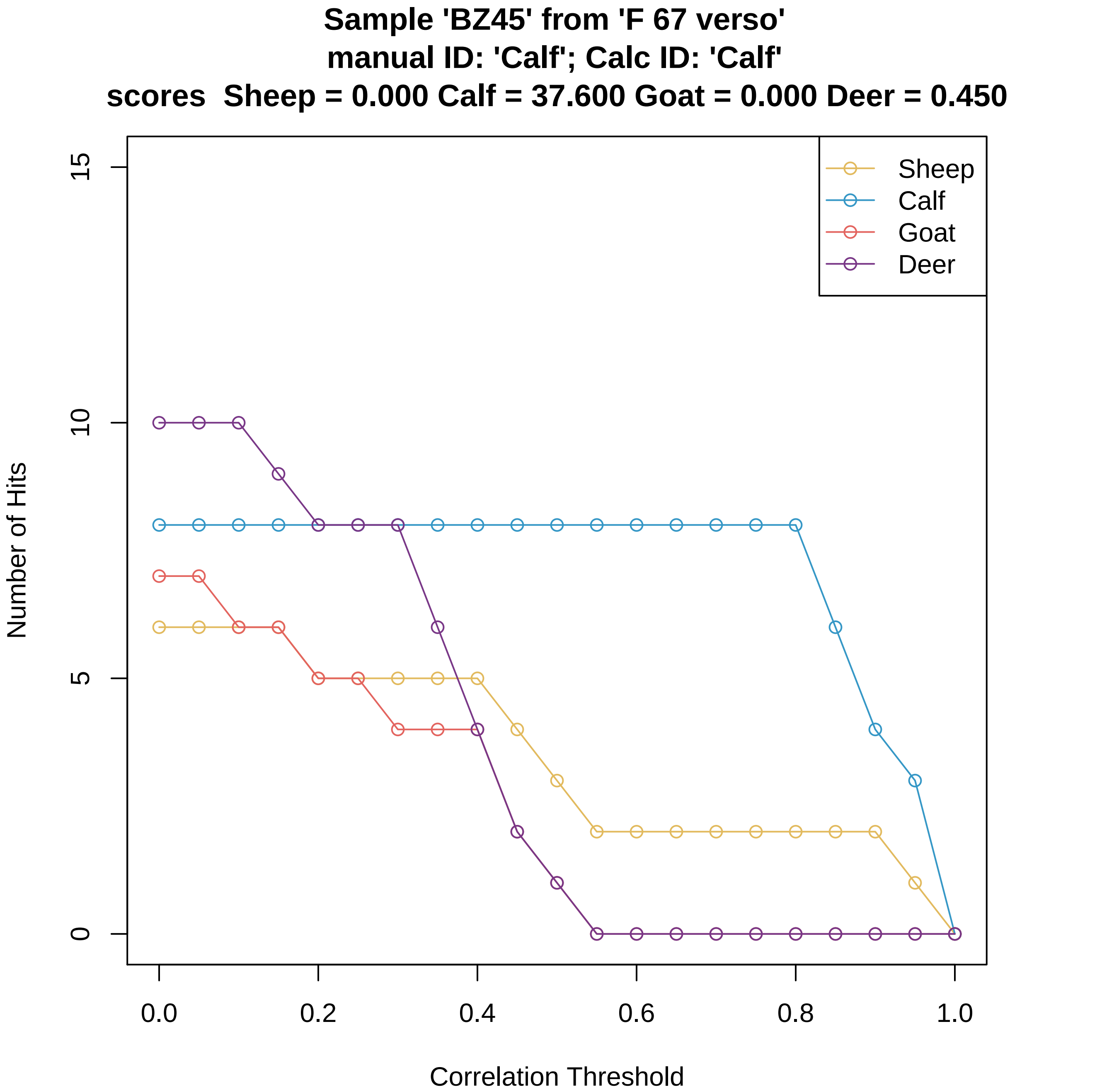
Sample 'BZ45' from 'F 67 verso'
manual ID: 'Calf'; Calc ID: 'Calf'
scores Sheep = 0.000 Calf = 37.600 Goat = 0.000 Deer = 0.450
Sheep
Calf
Goat
Deer
Number of Hits
0
5
10
15
0.0
0.2
0.4
0.6
0.8
1.0
Correlation Threshold

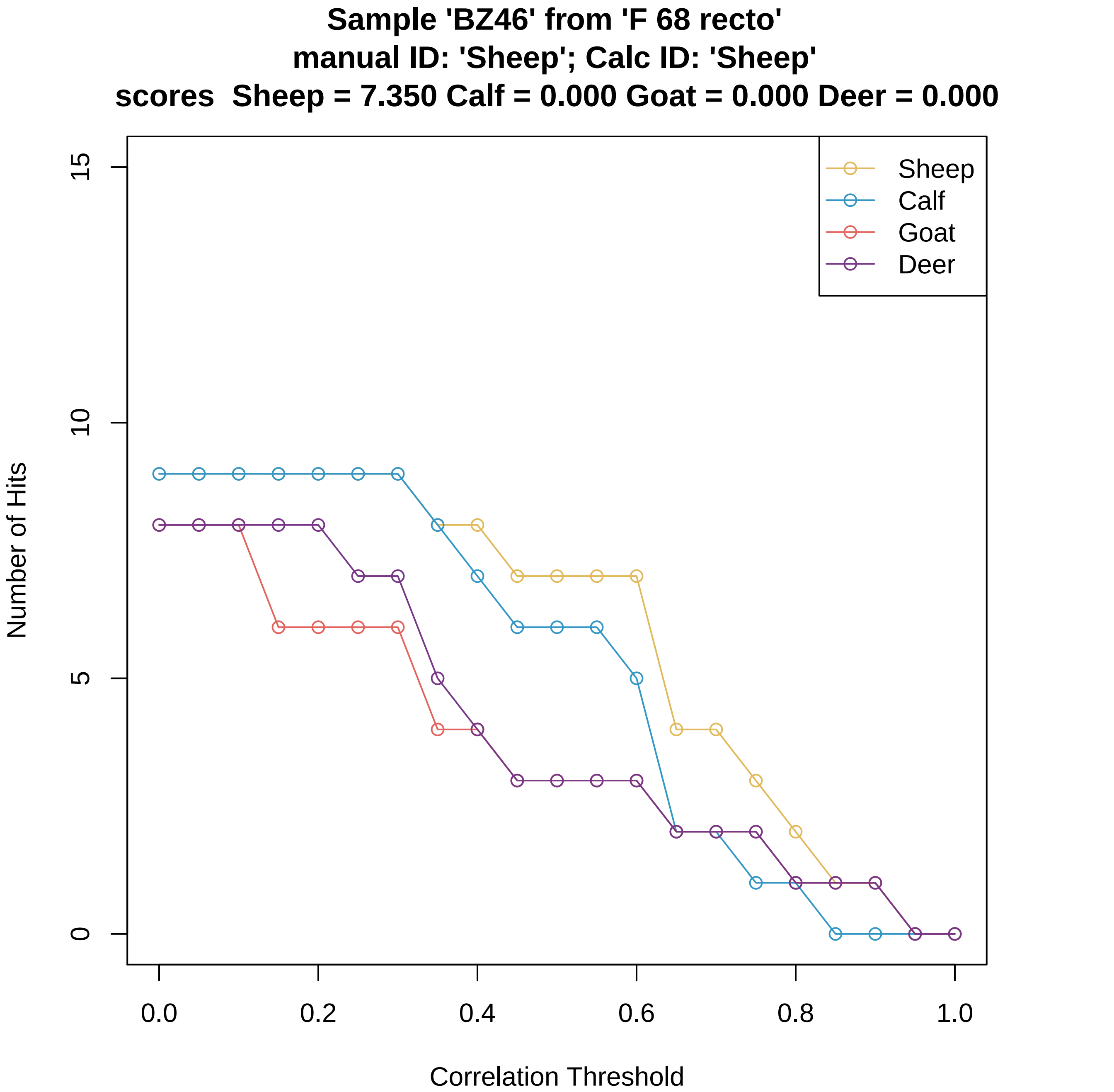

Sample 'BZ46' from 'F 68 recto'
manual ID: 'Sheep'; Calc ID: 'Sheep'
scores Sheep = 7.350 Calf = 0.000 Goat = 0.000 Deer = 0.000
Sheep
Calf
Goat
Deer
Number of Hits
0
5
10
15
0.0
0.2
0.4
0.6
0.8
1.0
Correlation Threshold

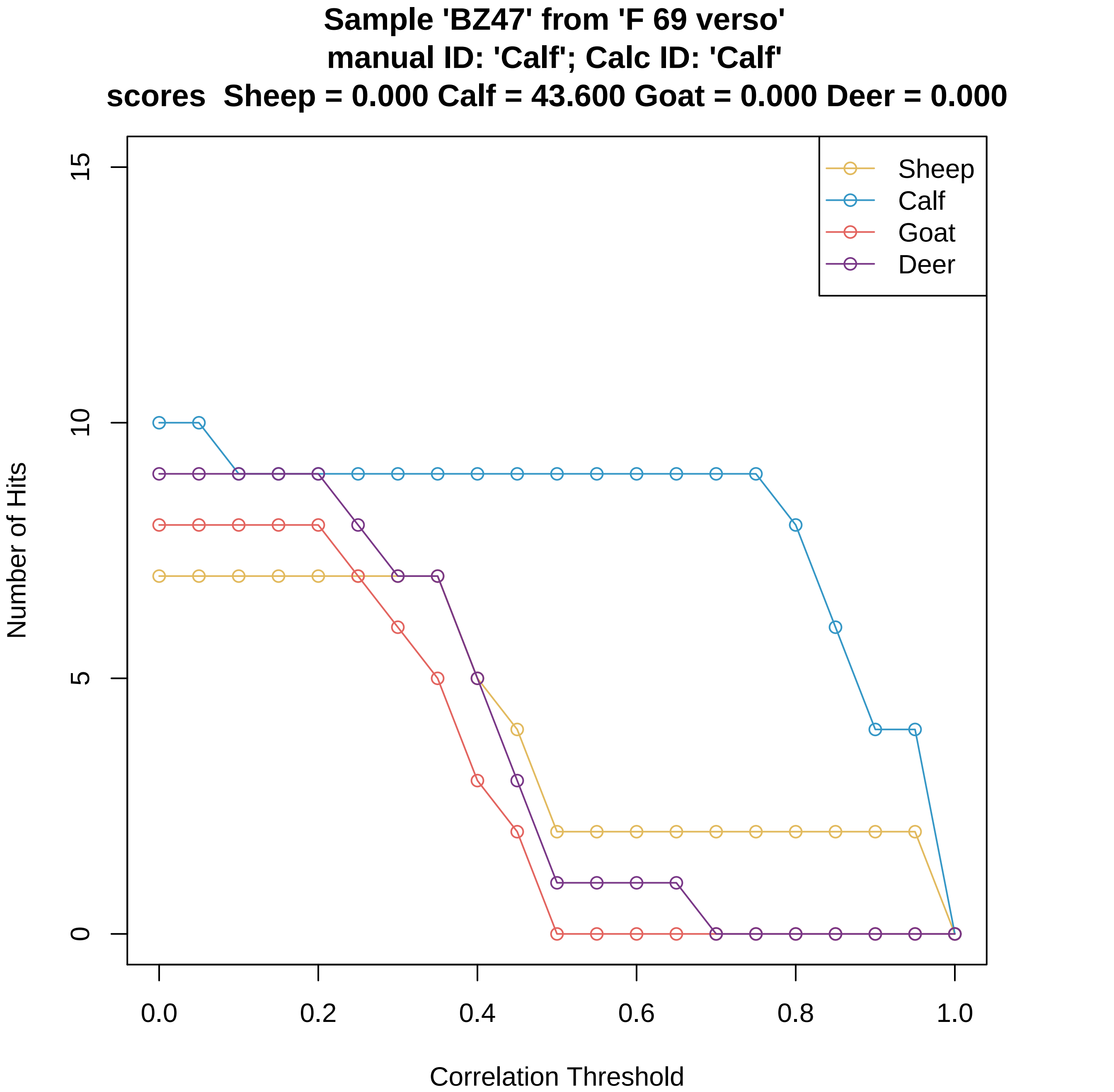
Sample 'BZ47' from 'F 69 verso'
manual ID: 'Calf'; Calc ID: 'Calf'
scores Sheep = 0.000 Calf = 43.600 Goat = 0.000 Deer = 0.000
Sheep
Calf
Goat
Deer
Number of Hits
0
5
10
15
0.0
0.2
0.4
0.6
0.8
1.0
Correlation Threshold

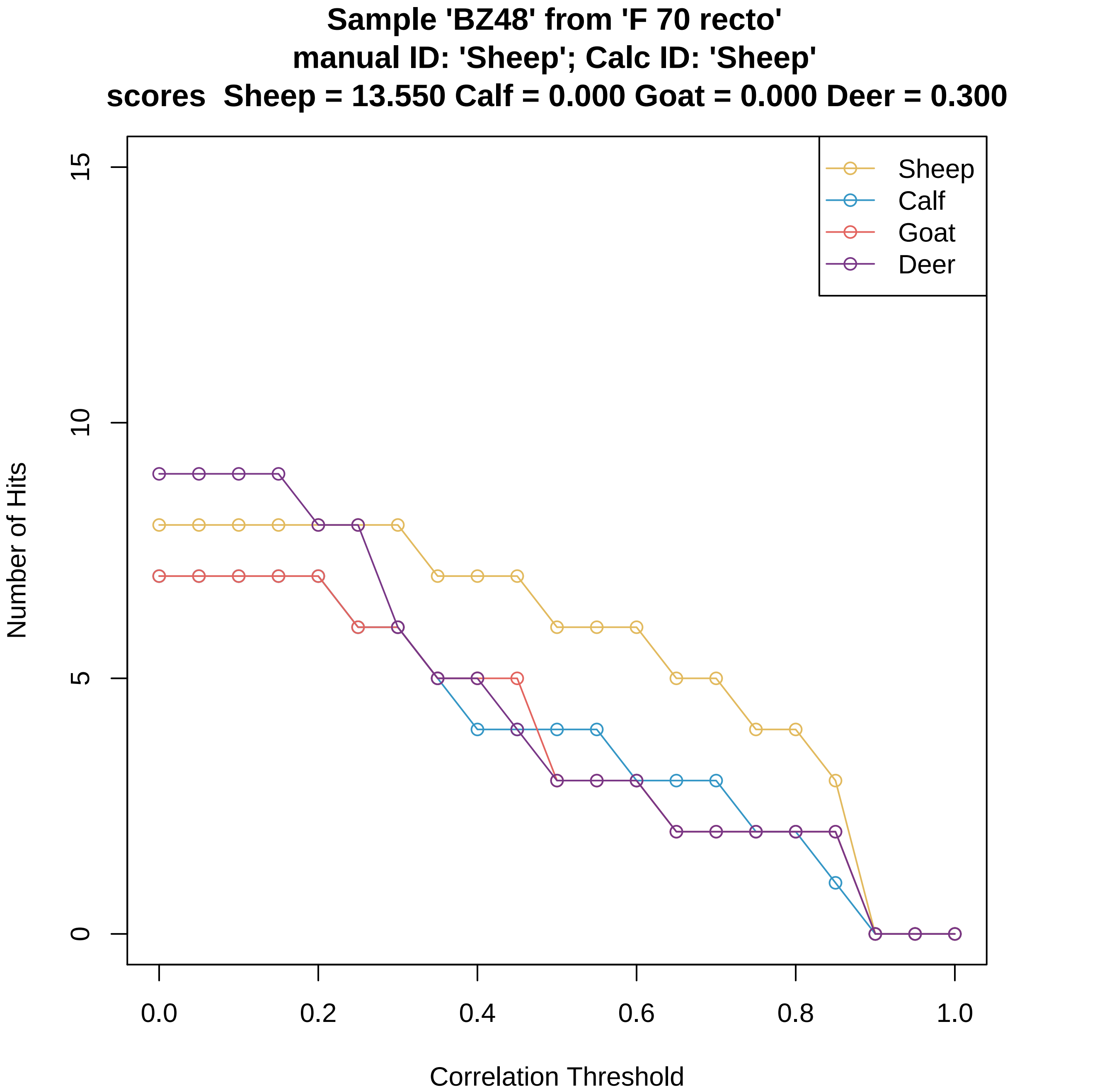
Sample 'BZ48' from 'F 70 recto'
manual ID: 'Sheep'; Calc ID: 'Sheep'
scores Sheep = 13.550 Calf = 0.000 Goat = 0.000 Deer = 0.300
Sheep
Calf
Goat
Deer
Number of Hits
0
5
10
15
0.0
0.2
0.4
0.6
0.8
1.0
Correlation Threshold

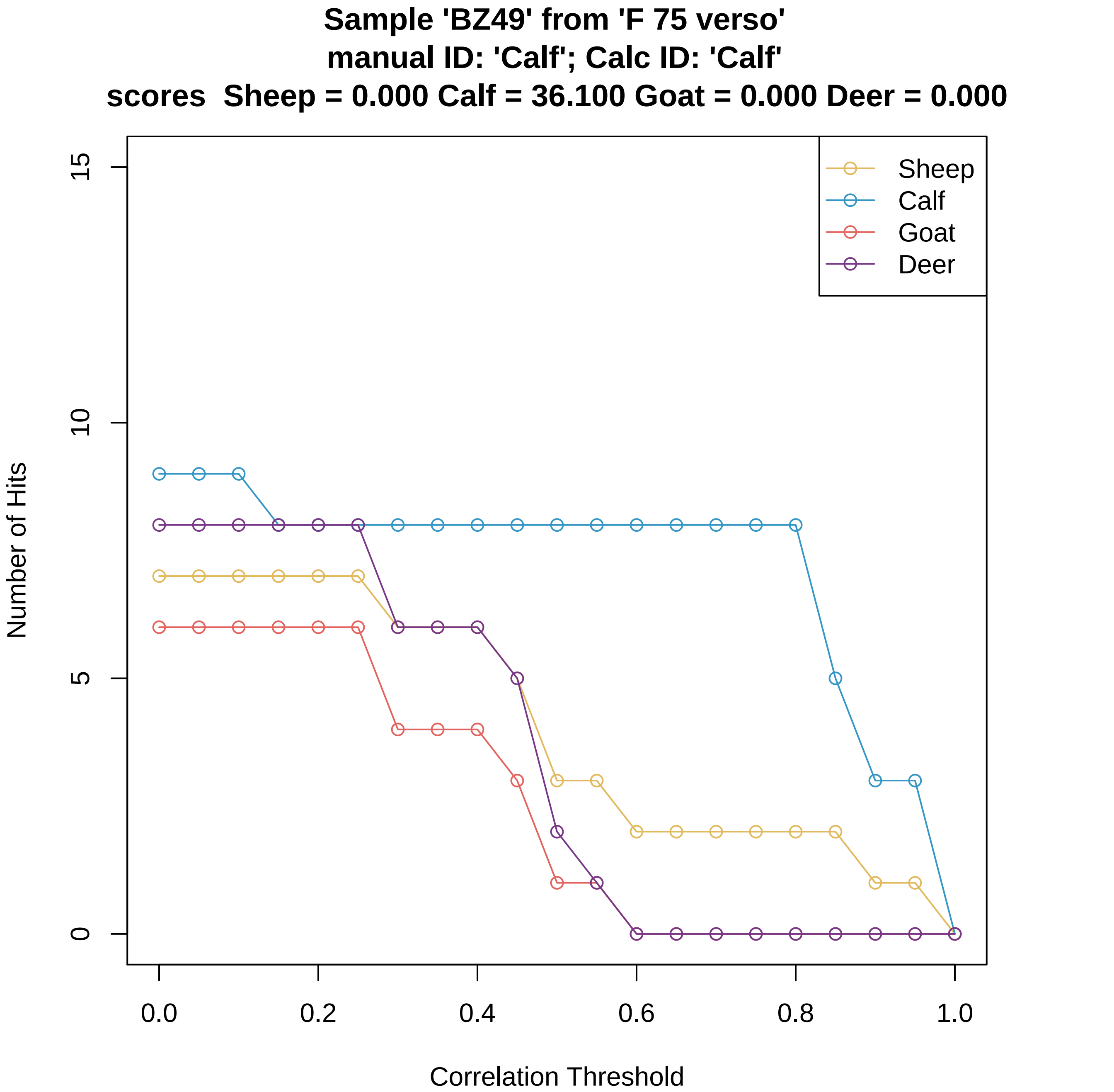

Sample 'BZ49' from 'F 75 verso'
manual ID: 'Calf'; Calc ID: 'Calf'
scores Sheep = 0.000 Calf = 36.100 Goat = 0.000 Deer = 0.000
Sheep
Calf
Goat
Deer
Number of Hits
Correlation Threshold
0
5
10
15
0.0
0.2
0.4
0.6
0.8
1.0

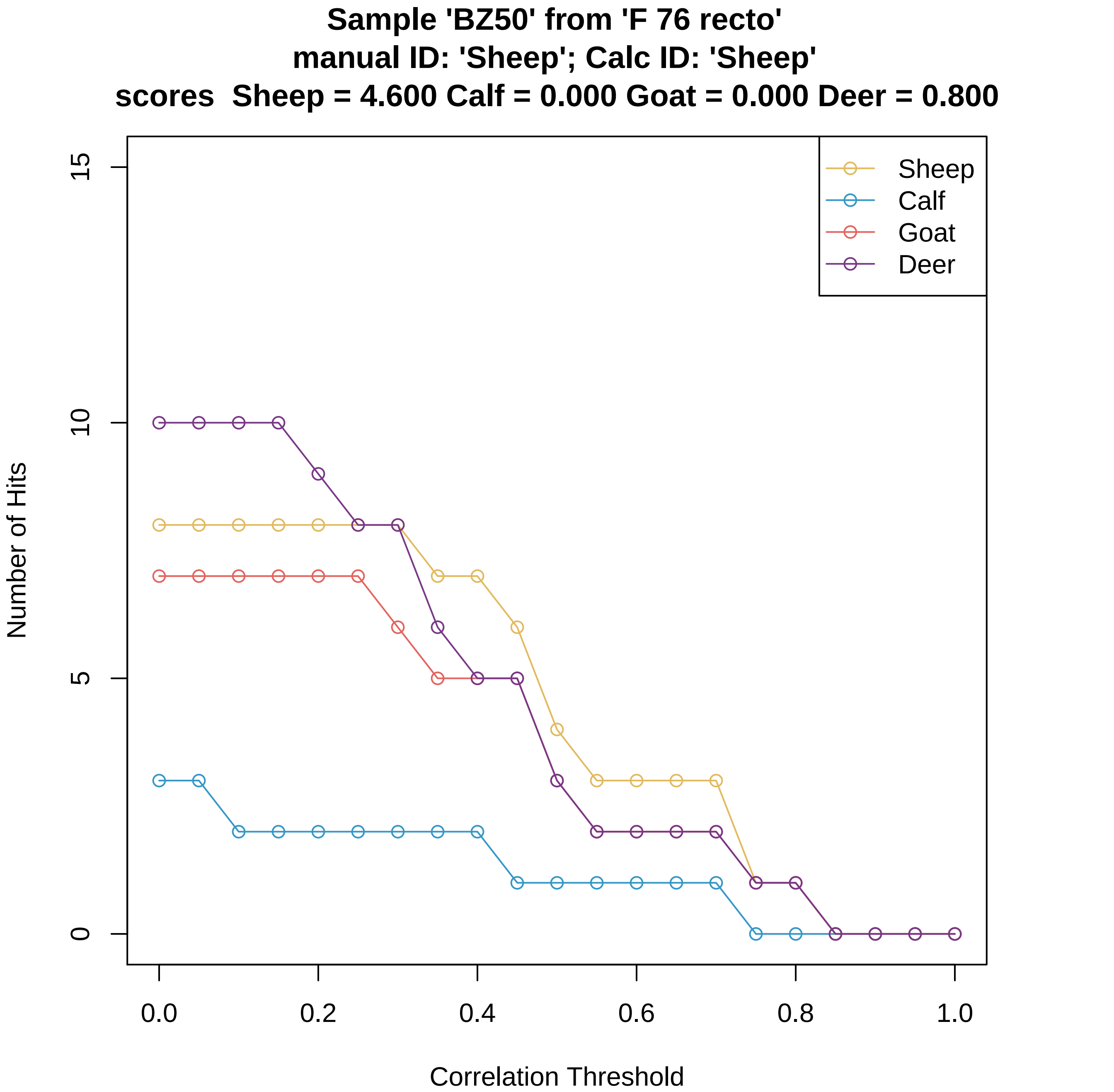
Sample 'BZ50' from 'F 76 recto'
manual ID: 'Sheep'; Calc ID: 'Sheep'
scores Sheep = 4.600 Calf = 0.000 Goat = 0.000 Deer = 0.800
Sheep
Calf
Goat
Deer
Number of Hits
0
5
10
15
0.0
0.2
0.4
0.6
0.8
1.0
Correlation Threshold

Sample 'BZ51' from 'F 77 verso'
manual ID: 'Calf'; Calc ID: 'Calf'
scores Sheep = 0.000 Calf = 40.650 Goat = 0.000 Deer = 0.000

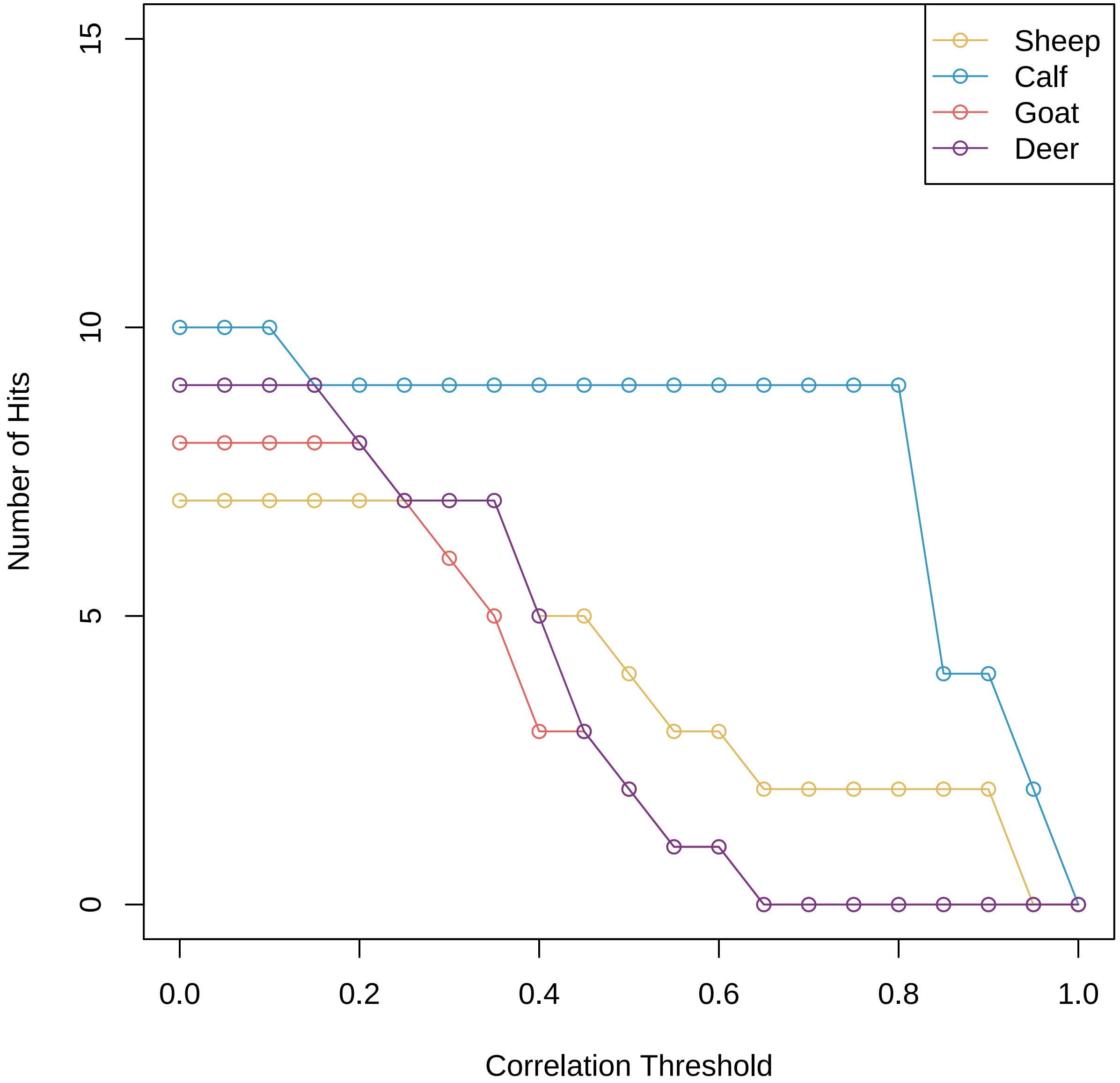

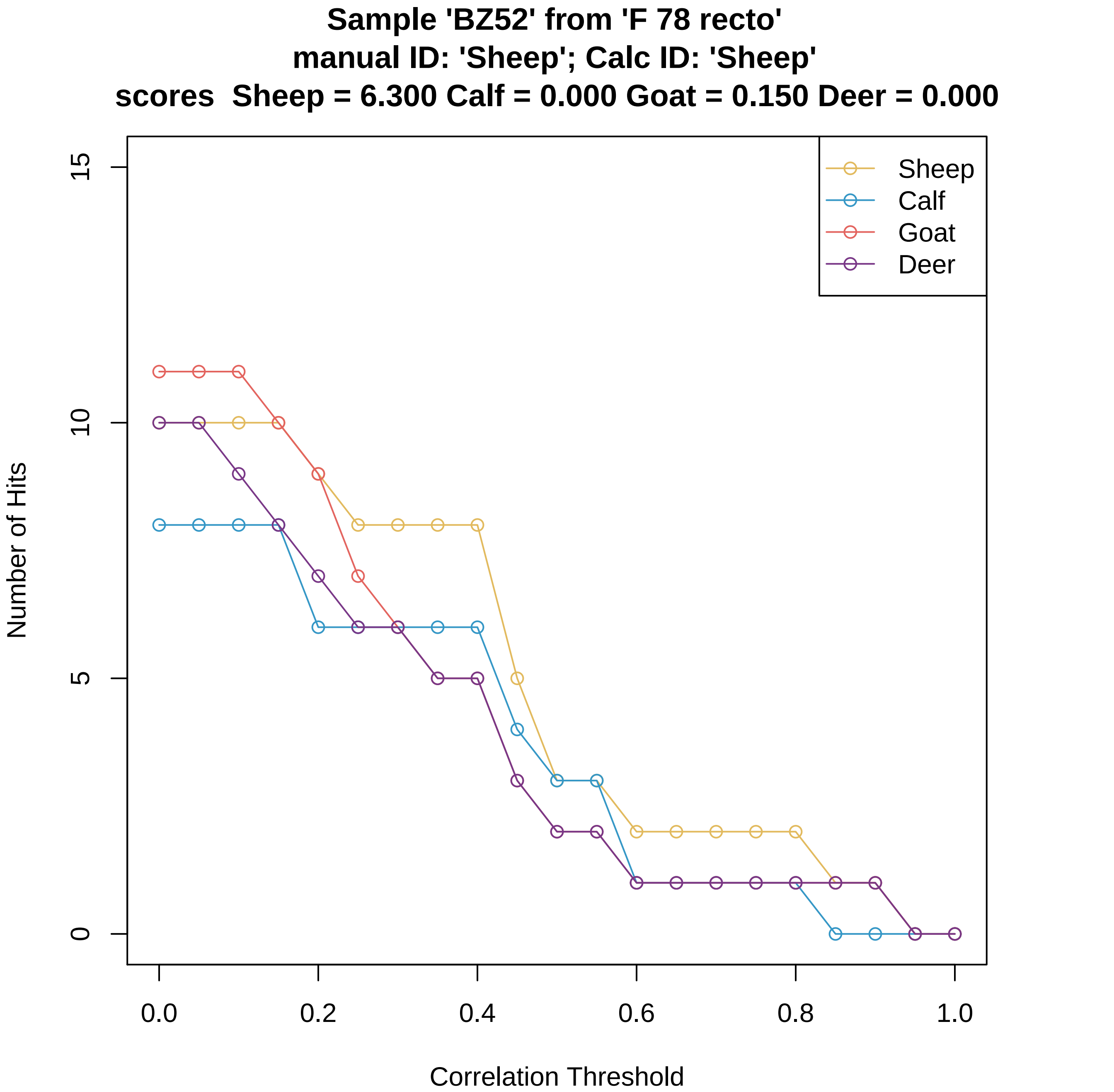

Sample 'BZ52' from 'F 78 recto'
manual ID: 'Sheep'; Calc ID: 'Sheep'
scores Sheep = 6.300 Calf = 0.000 Goat = 0.150 Deer = 0.000
Sheep
Calf
Goat
Deer
Number of Hits
0
5
10
15
0.0
0.2
0.4
0.6
0.8
1.0
Correlation Threshold

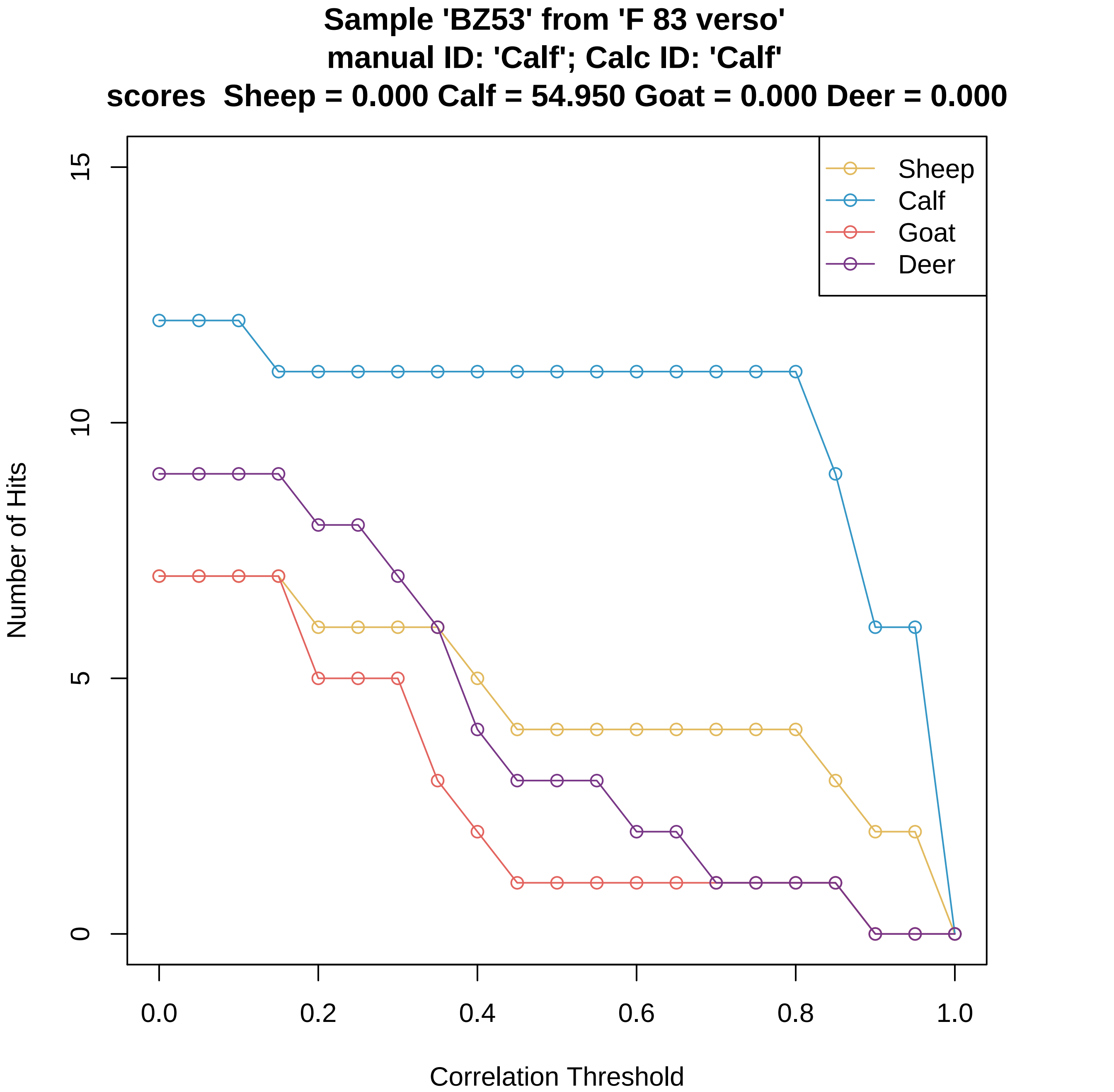
Sample 'BZ53' from 'F 83 verso'
manual ID: 'Calf'; Calc ID: 'Calf'
scores Sheep = 0.000 Calf = 54.950 Goat = 0.000 Deer = 0.000
Sheep
Calf
Goat
Deer
Number of Hits
Correlation Threshold
0
5
10
15
0.0
0.2
0.4
0.6
0.8
1.0

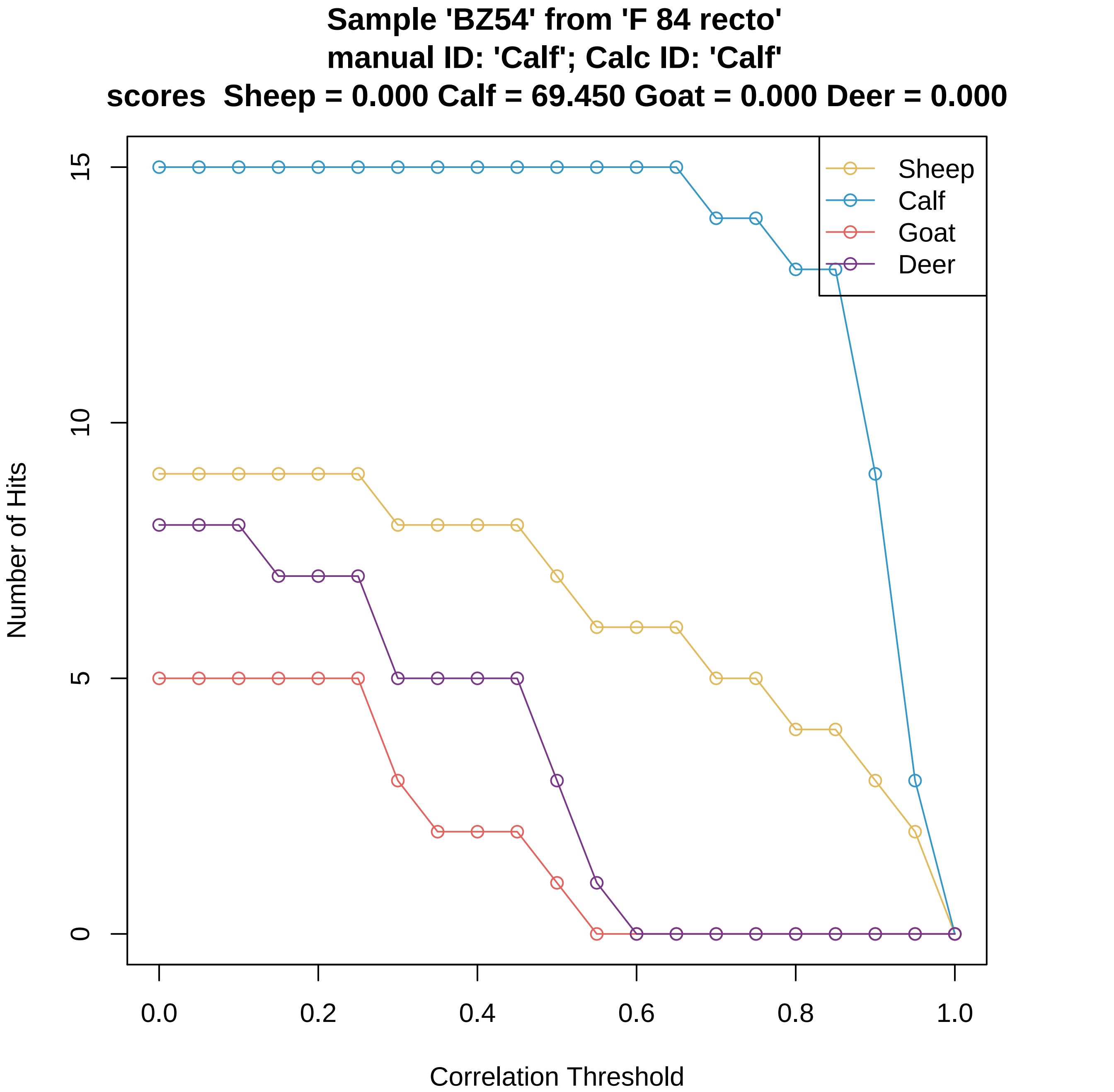

Sample 'BZ54' from 'F 84 recto'
manual ID: 'Calf'; Calc ID: 'Calf'
scores Sheep = 0.000 Calf = 69.450 Goat = 0.000 Deer = 0.000
Sheep
Calf
Goat
Deer
Number of Hits
0
5
10
15
0.0
0.2
0.4
0.6
0.8
1.0
Correlation Threshold

Sample 'BZ55' from 'F 85 verso'
manual ID: 'Calf'; Calc ID: 'Calf'
scores Sheep = 0.000 Calf = 73.500 Goat = 0.000 Deer = 0.000

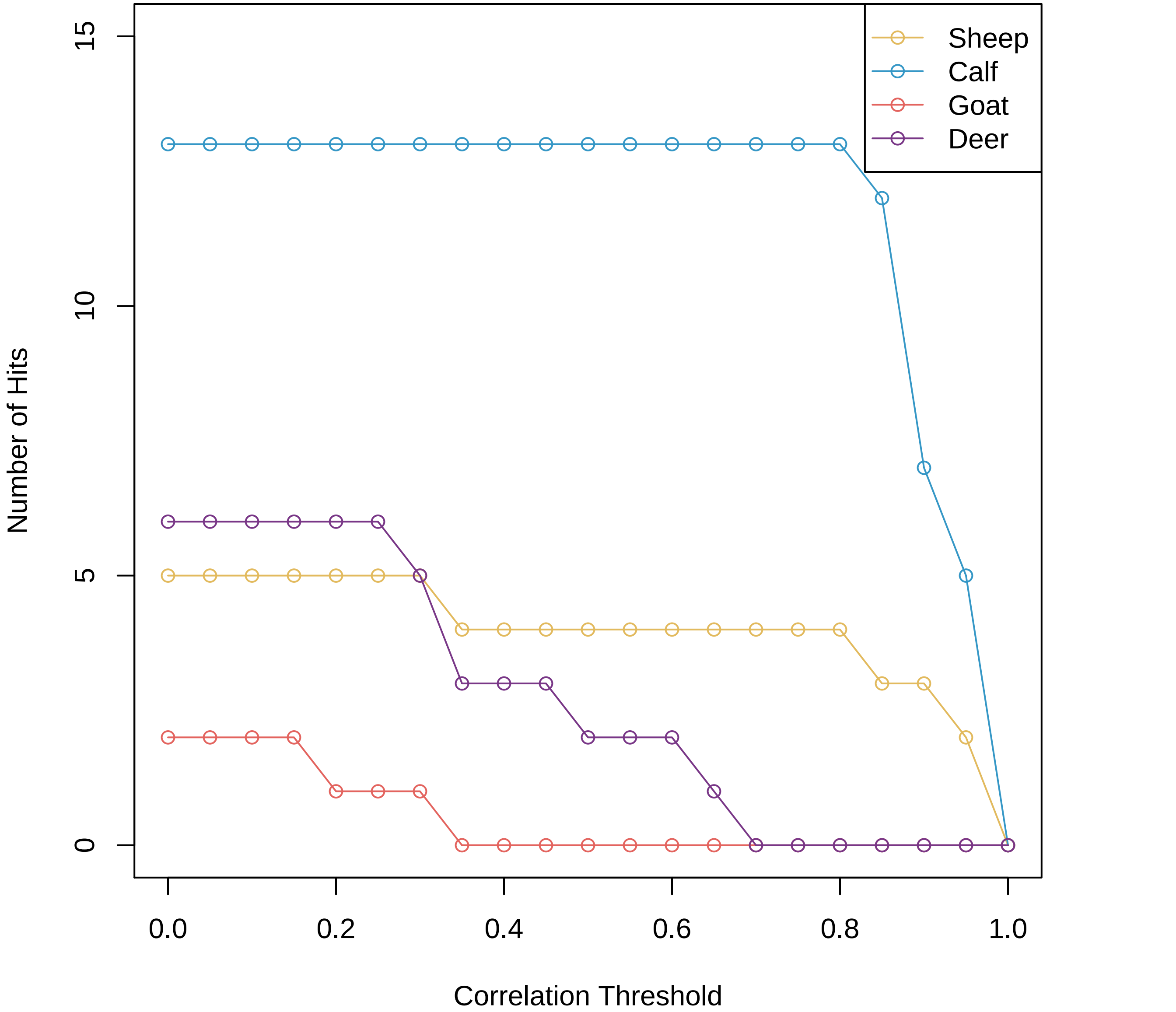

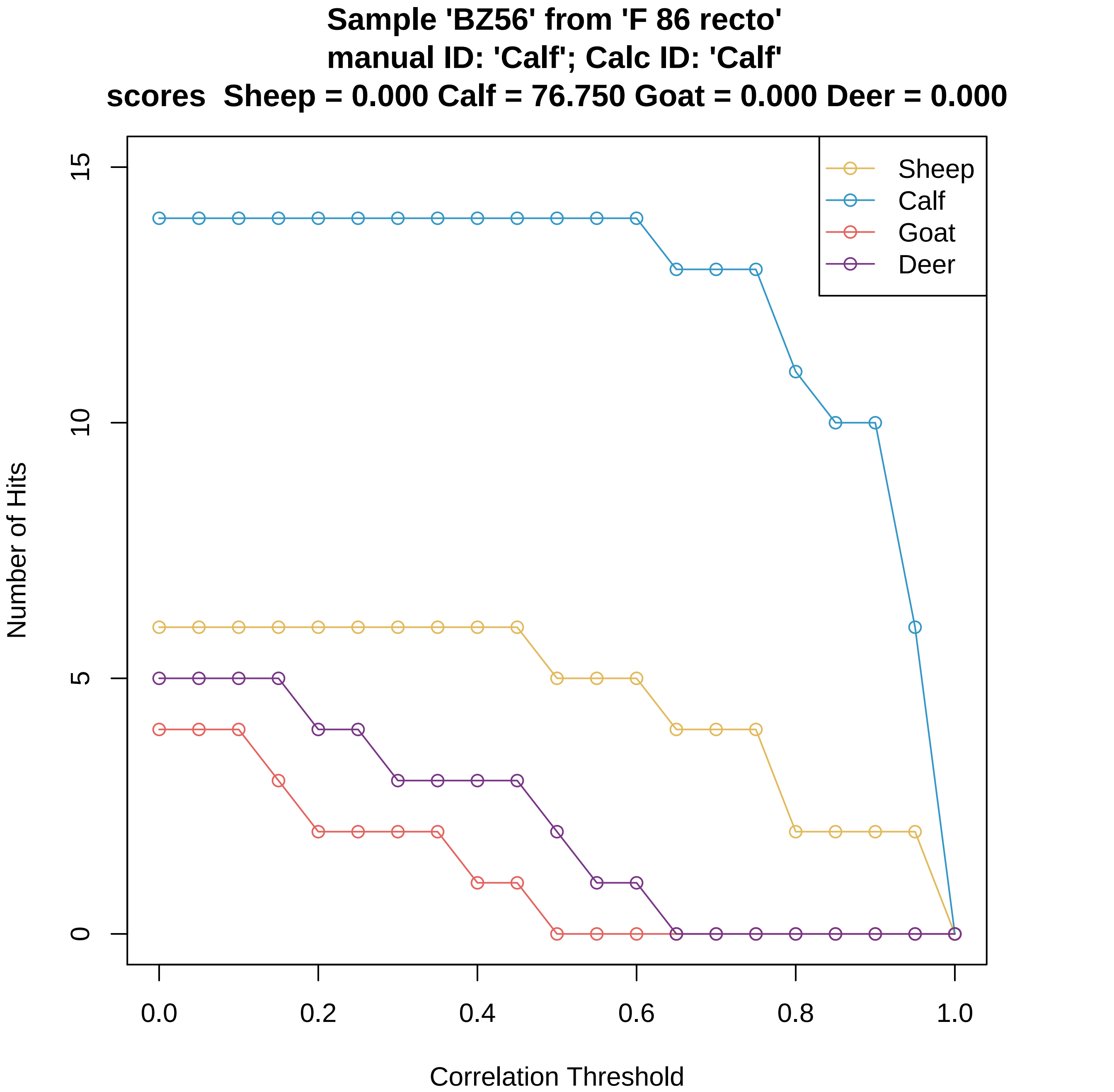
Sample 'BZ56' from 'F 86 recto'
manual ID: 'Calf'; Calc ID: 'Calf'
scores Sheep = 0.000 Calf = 76.750 Goat = 0.000 Deer = 0.000
Number of Hits
Correlation Threshold
0
5
10
15
0.0
0.2
0.4
0.6
0.8
1.0
Sheep
Calf
Goat
Deer

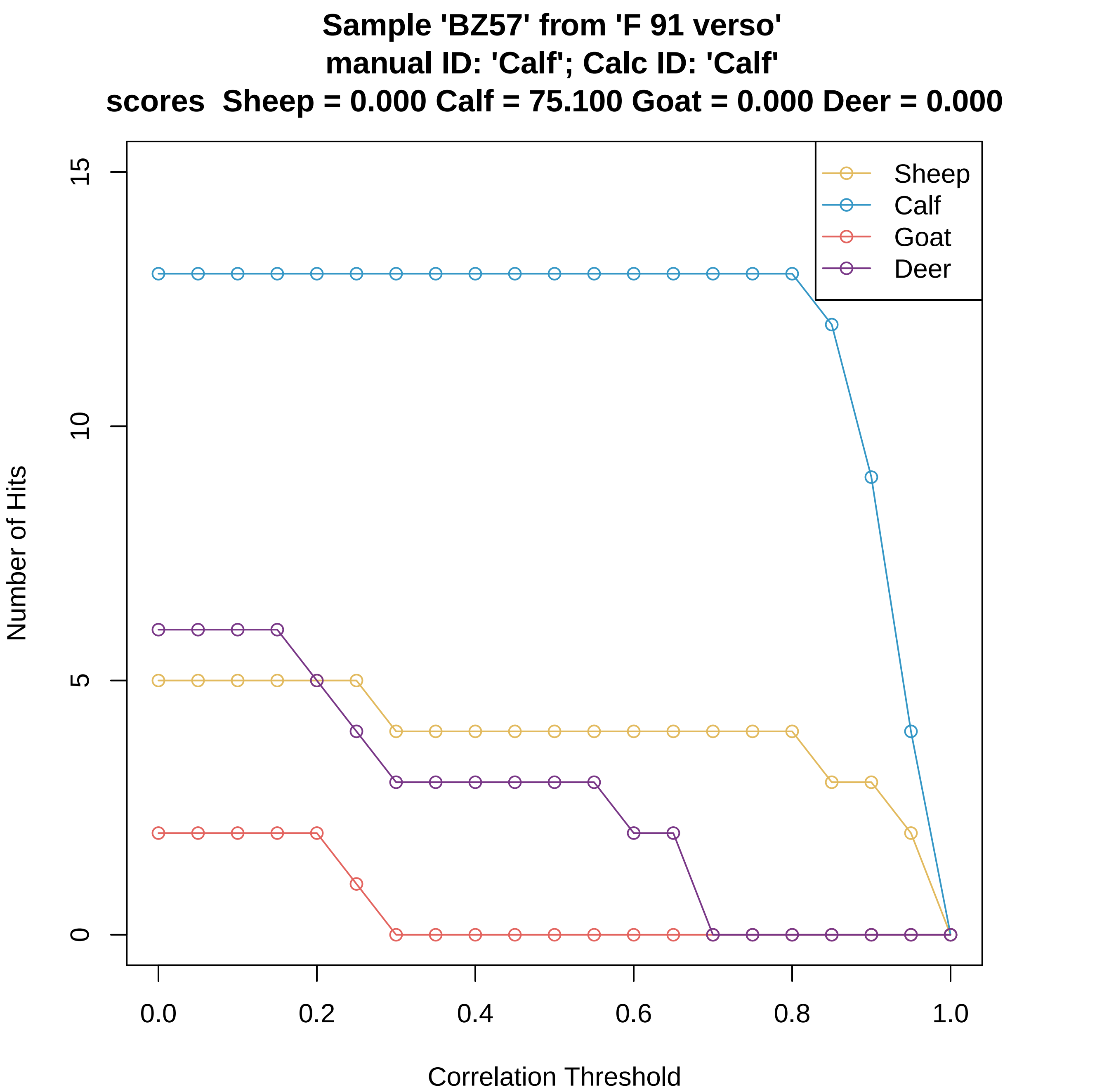
Sample 'BZ57' from 'F 91 verso'
manual ID: 'Calf'; Calc ID: 'Calf'
scores Sheep = 0.000 Calf = 75.100 Goat = 0.000 Deer = 0.000
Sheep
Calf
Goat
Deer
Number of Hits
Correlation Threshold
0
5
10
15
0.0
0.2
0.4
0.6
0.8
1.0

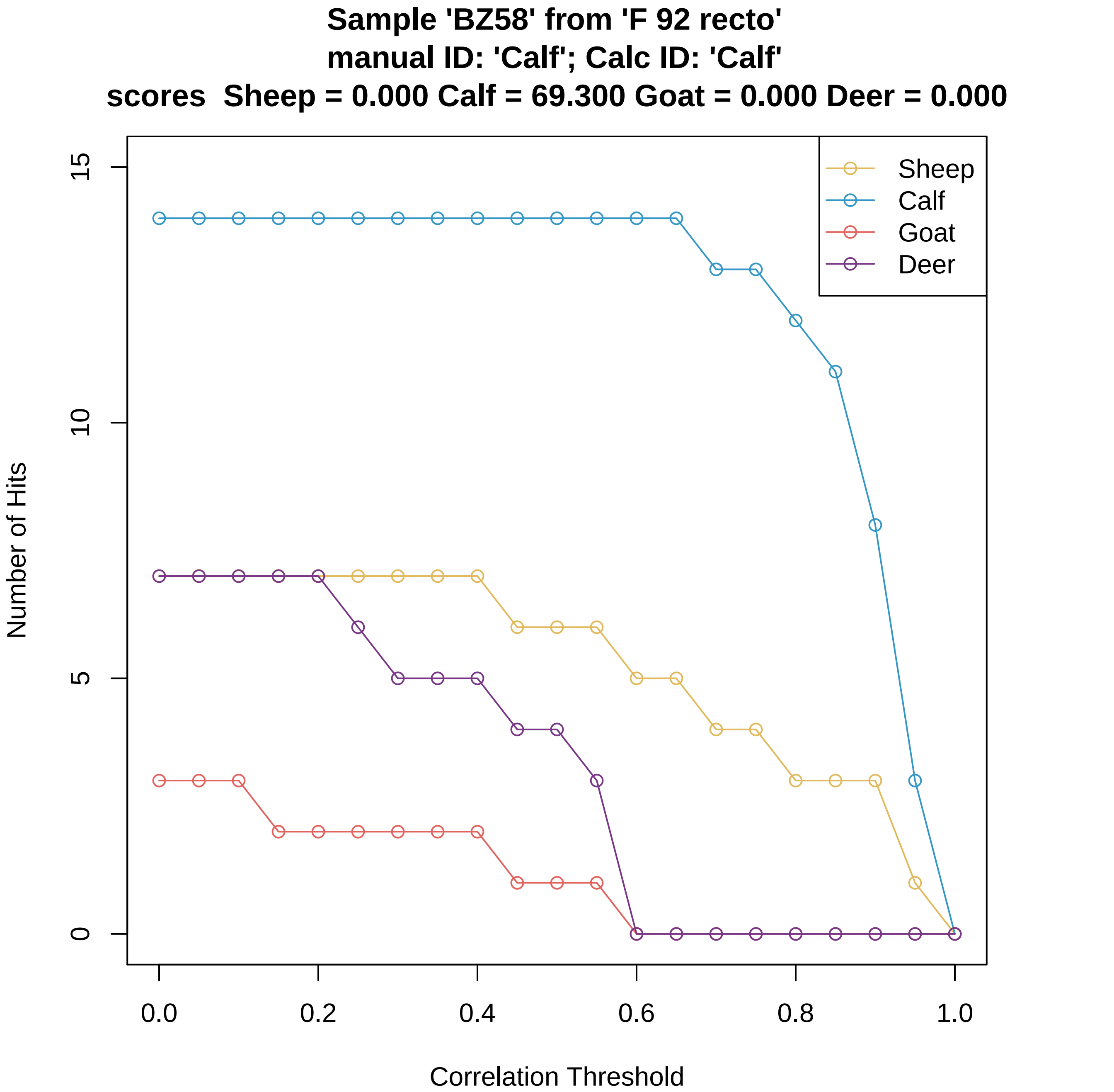

Sample 'BZ58' from 'F 92 recto'
manual ID: 'Calf'; Calc ID: 'Calf'
scores Sheep = 0.000 Calf = 69.300 Goat = 0.000 Deer = 0.000
Sheep
Calf
Goat
Deer
Number of Hits
0
5
10
15
0.0
0.2
0.4
0.6
0.8
1.0
Correlation Threshold

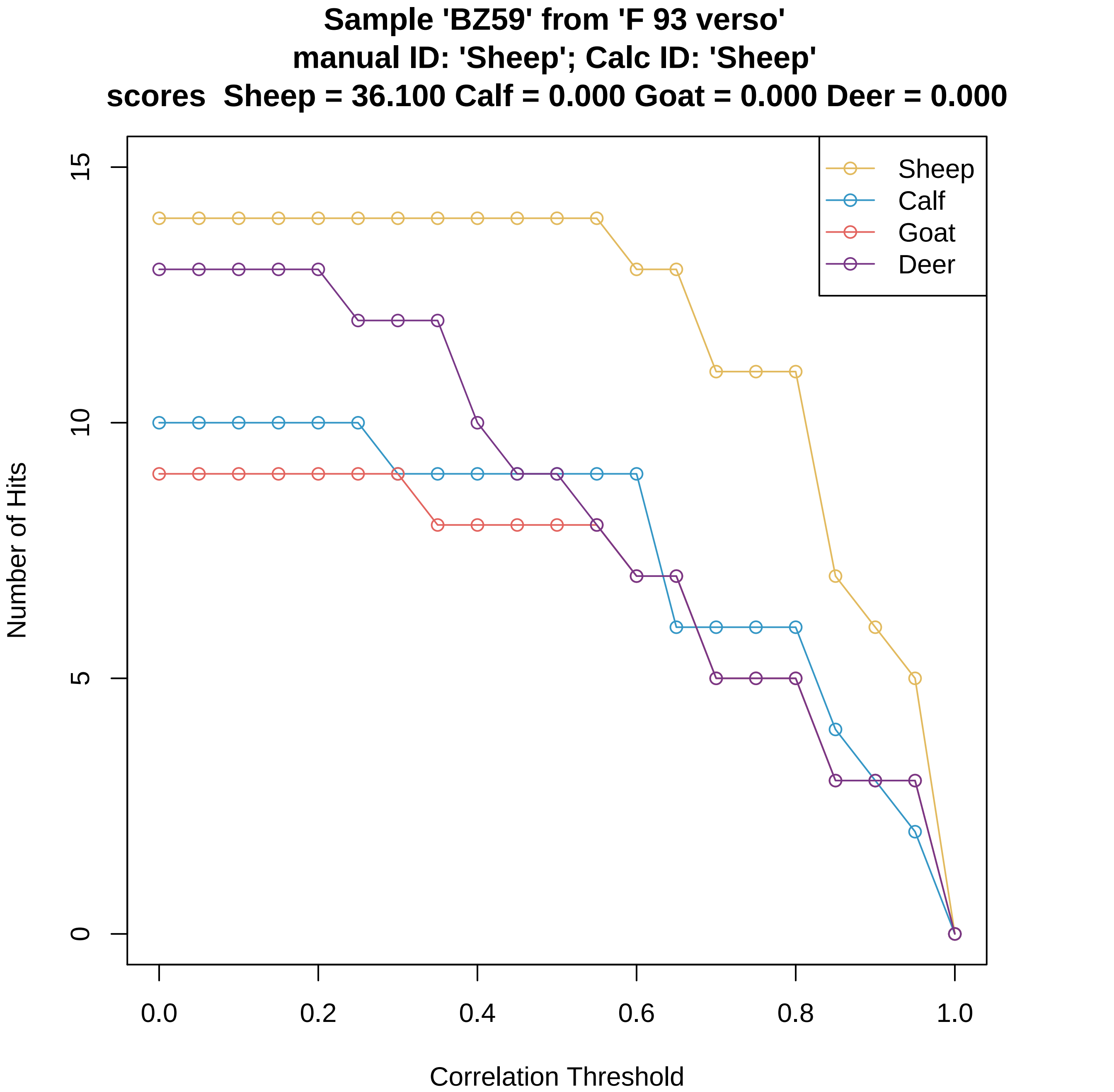
Sample 'BZ59' from 'F 93 verso'
manual ID: 'Sheep'; Calc ID: 'Sheep'
scores Sheep = 36.100 Calf = 0.000 Goat = 0.000 Deer = 0.000
Sheep
Calf
Goat
Deer
Number of Hits
Correlation Threshold
0
5
10
15
0.0
0.2
0.4
0.6
0.8
1.0

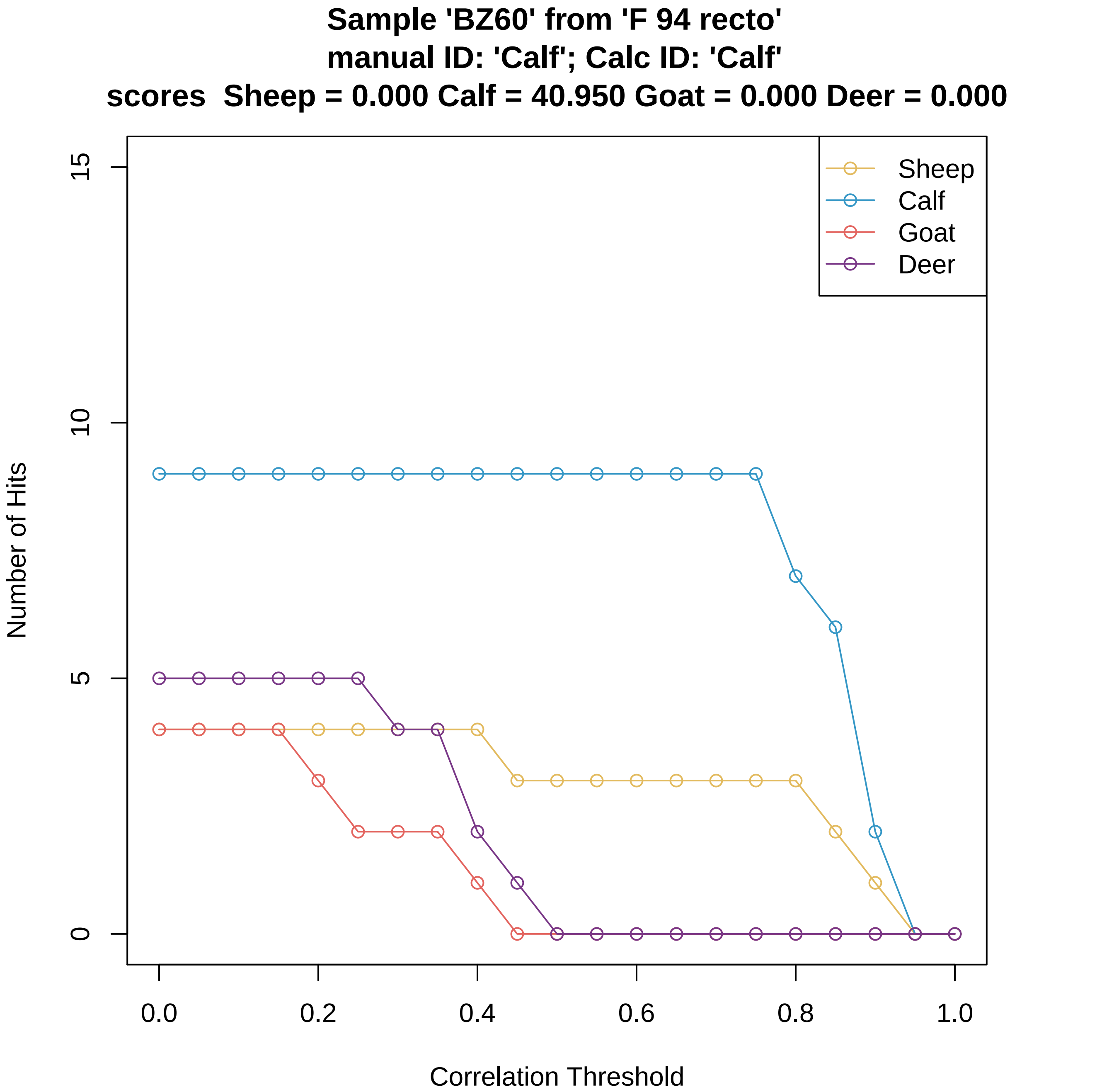

Sample 'BZ60' from 'F 94 recto'
manual ID: 'Calf'; Calc ID: 'Calf'
scores Sheep = 0.000 Calf = 40.950 Goat = 0.000 Deer = 0.000
Sheep
Calf
Goat
Deer
Number of Hits
0
5
10
15
0.0
0.2
0.4
0.6
0.8
1.0
Correlation Threshold

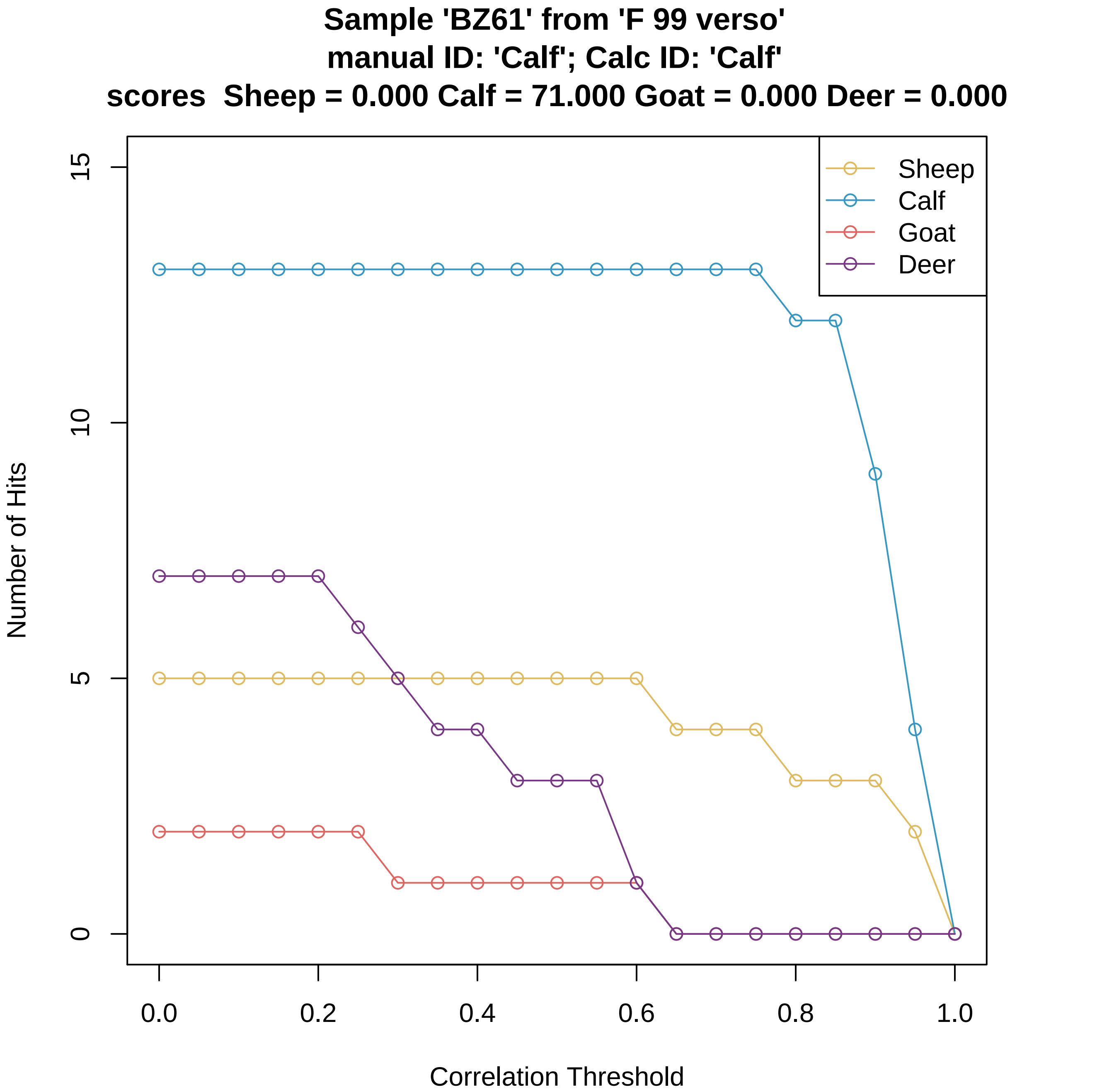
Sample 'BZ61' from 'F 99 verso'
manual ID: 'Calf'; Calc ID: 'Calf'
scores Sheep = 0.000 Calf = 71.000 Goat = 0.000 Deer = 0.000
Sheep
Calf
Goat
Deer
Number of Hits
0
5
10
15
0.0
0.2
0.4
0.6
0.8
1.0
Correlation Threshold

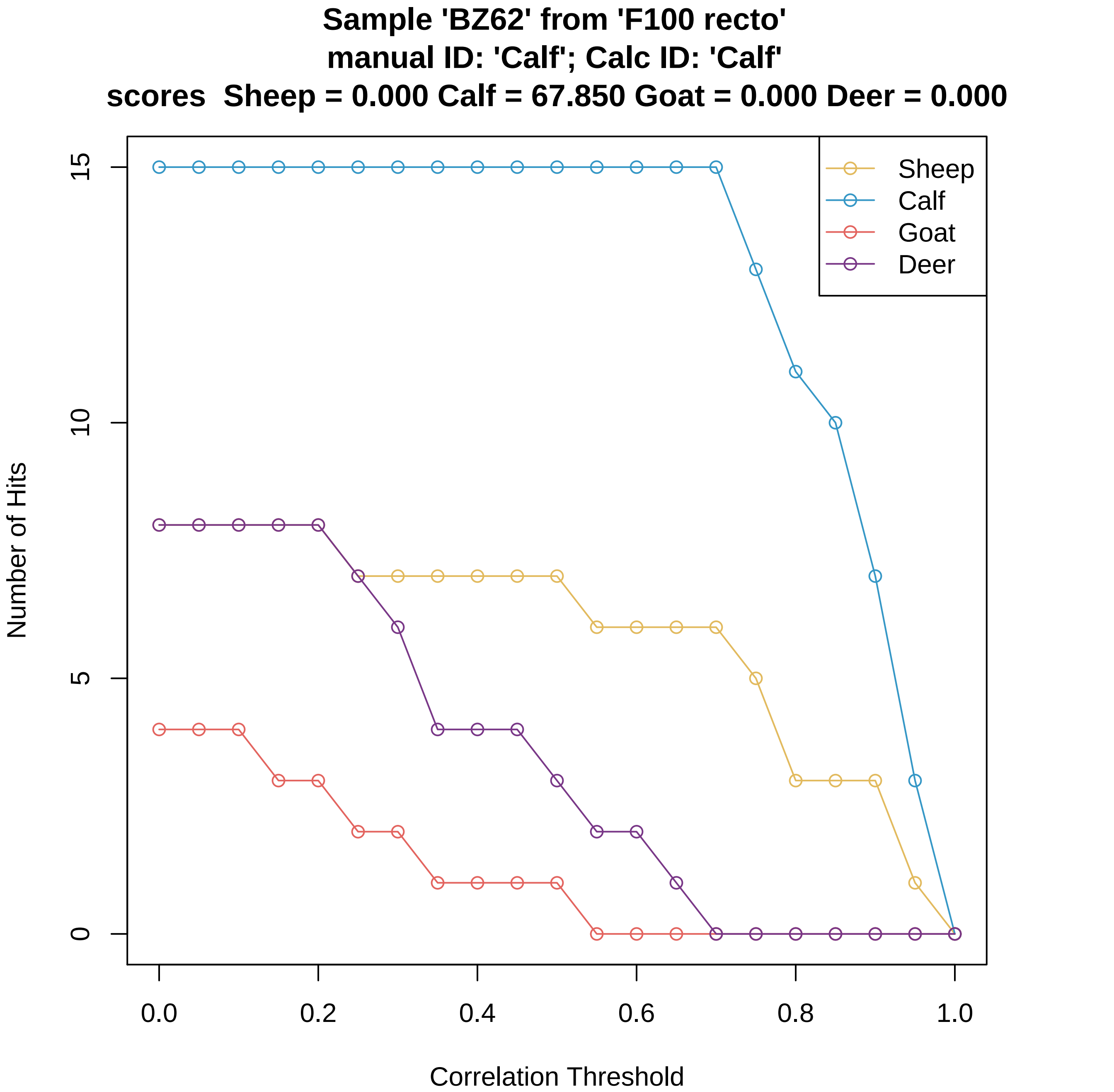

Sample 'BZ62' from 'F100 recto'
manual ID: 'Calf'; Calc ID: 'Calf'
scores Sheep = 0.000 Calf = 67.850 Goat = 0.000 Deer = 0.000
Sheep
Calf
Goat
Deer
Number of Hits
Correlation Threshold
0
5
10
15
0.0
0.2
0.4
0.6
0.8
1.0

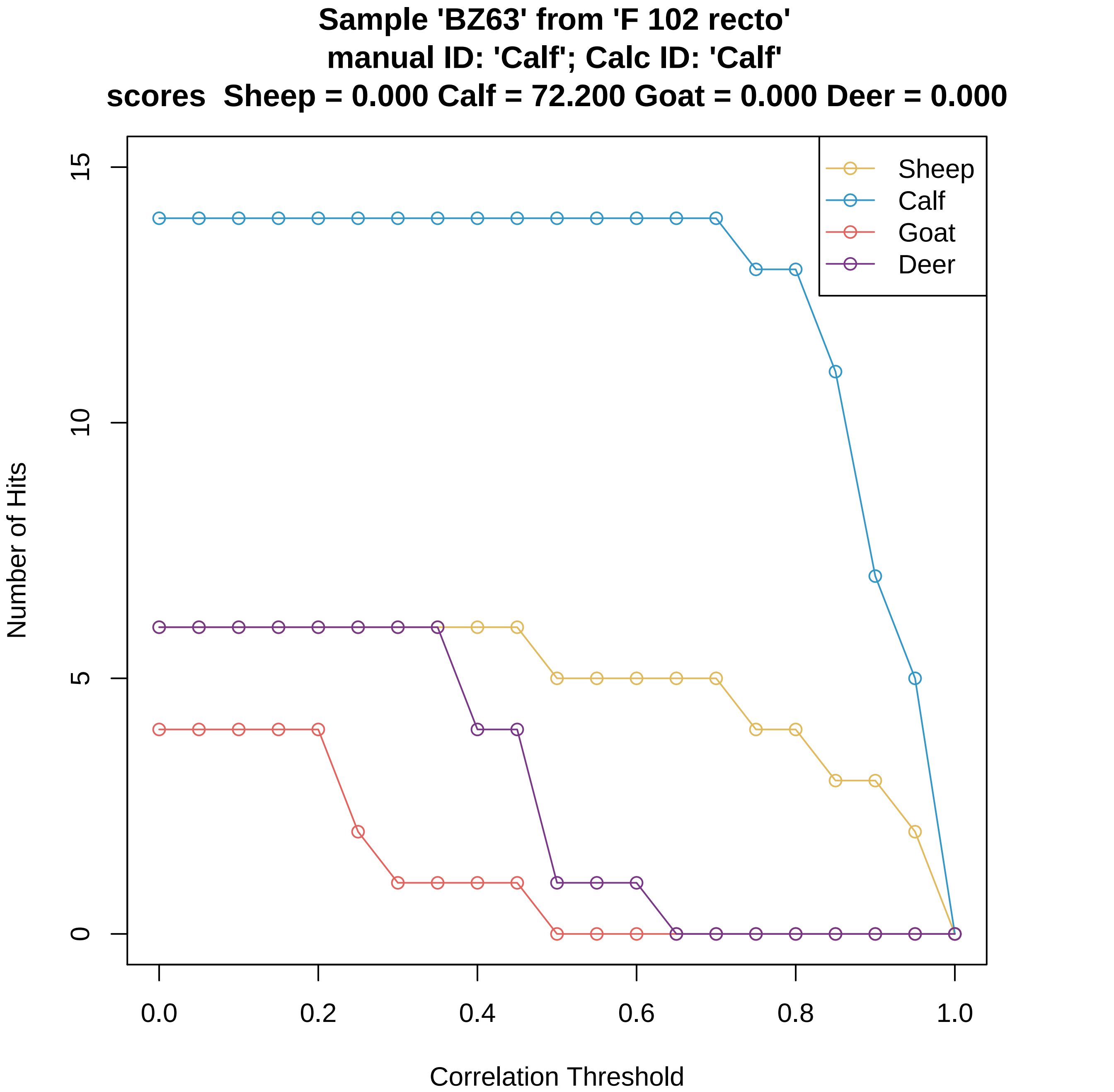
Sample 'BZ63' from 'F 102 recto'
manual ID: 'Calf'; Calc ID: 'Calf'
scores Sheep = 0.000 Calf = 72.200 Goat = 0.000 Deer = 0.000
Sheep
Calf
Goat
Deer
Number of Hits
0
5
10
15
0.0
0.2
0.4
0.6
0.8
1.0
Correlation Threshold

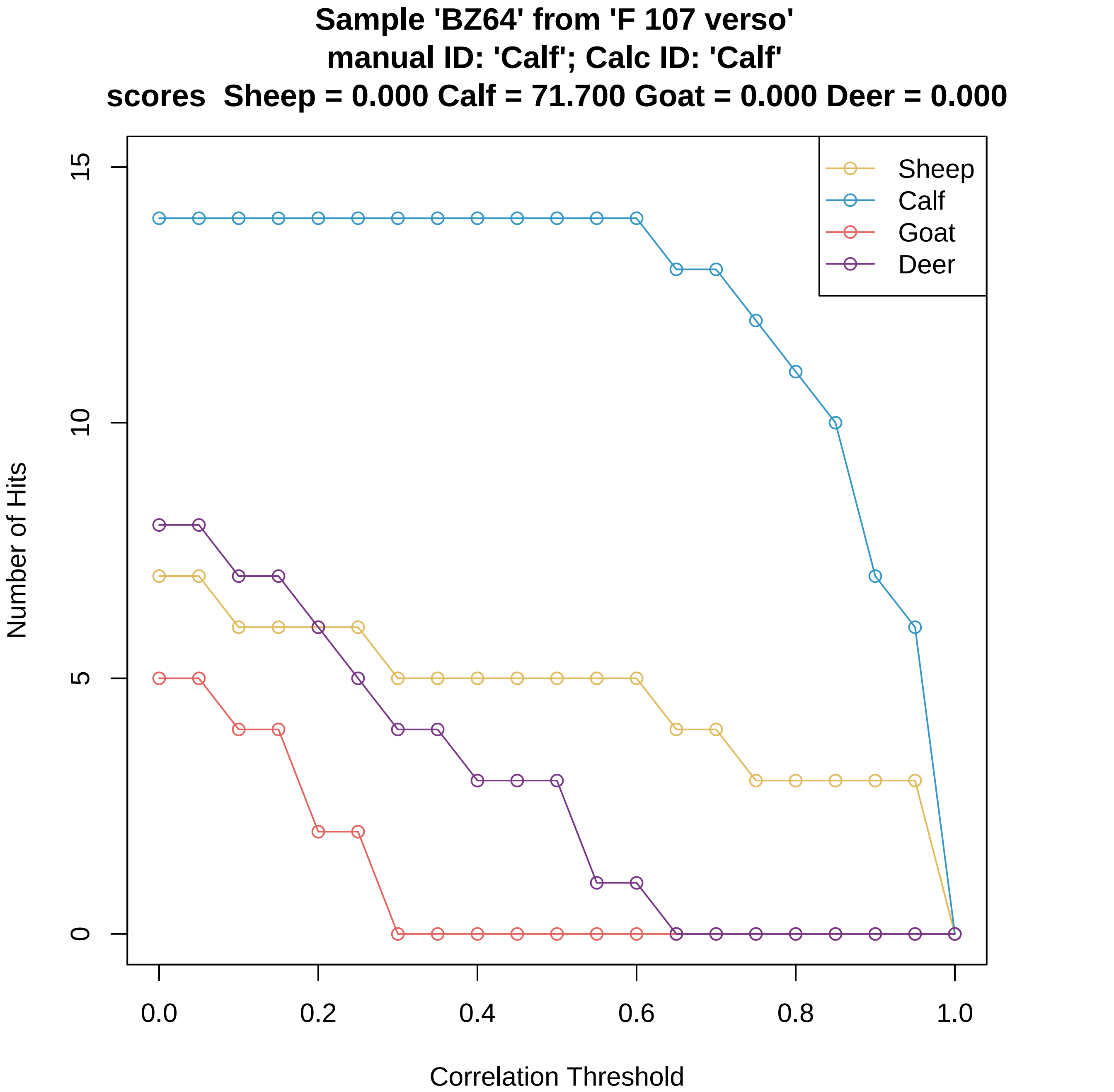

Sample 'BZ64' from 'F 107 verso'
manual ID: 'Calf'; Calc ID: 'Calf'
scores Sheep = 0.000 Calf = 71.700 Goat = 0.000 Deer = 0.000
Sheep
Calf
Goat
Deer
Number of Hits
0
5
10
15
0.0
0.2
0.4
0.6
0.8
1.0
Correlation Threshold

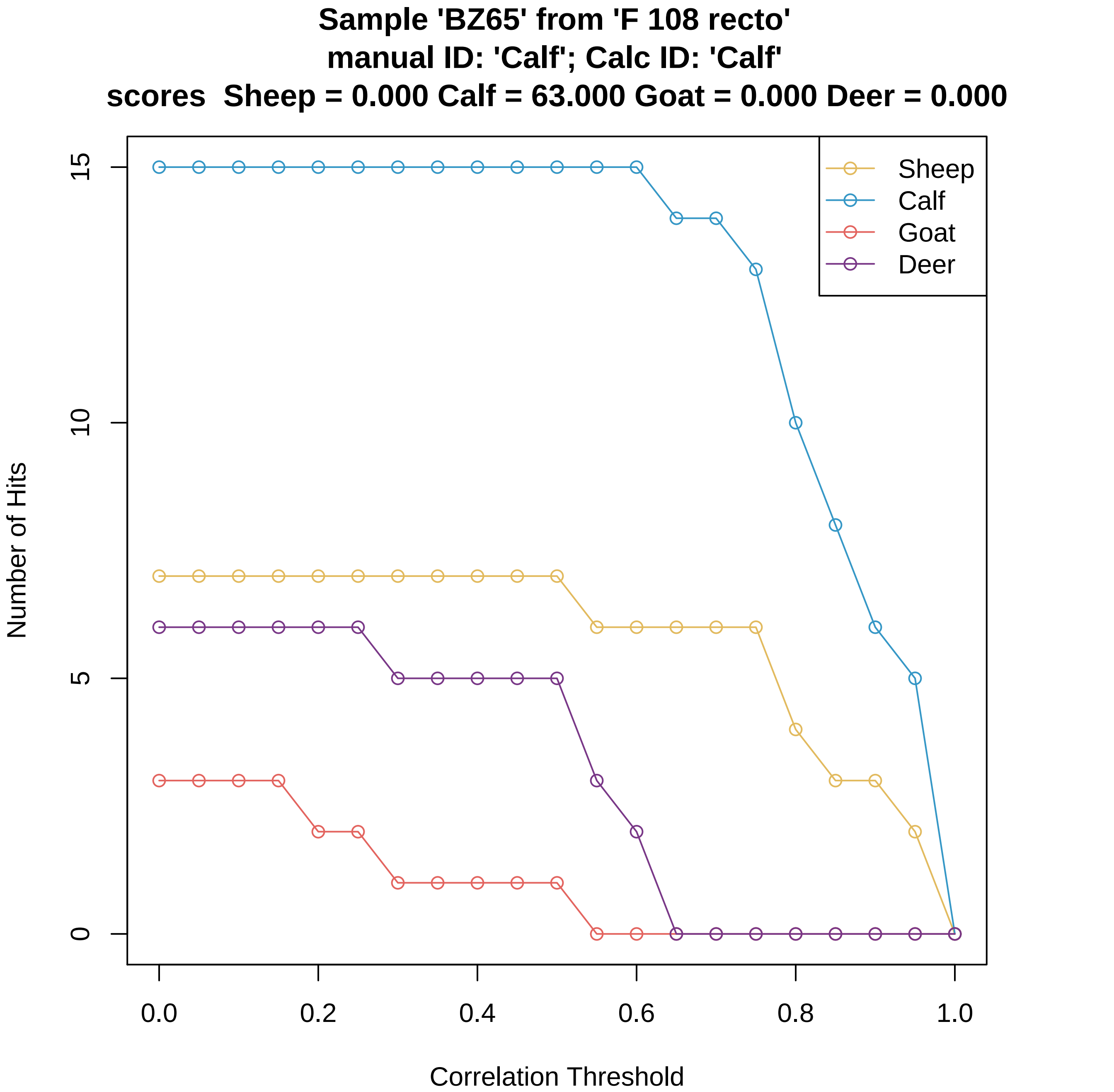

Sample 'BZ65' from 'F 108 recto'
manual ID: 'Calf'; Calc ID: 'Calf'
scores Sheep = 0.000 Calf = 63.000 Goat = 0.000 Deer = 0.000
Sheep
Calf
Goat
Deer
Number of Hits
0
5
10
15
0.0
0.2
0.4
0.6
0.8
1.0
Correlation Threshold

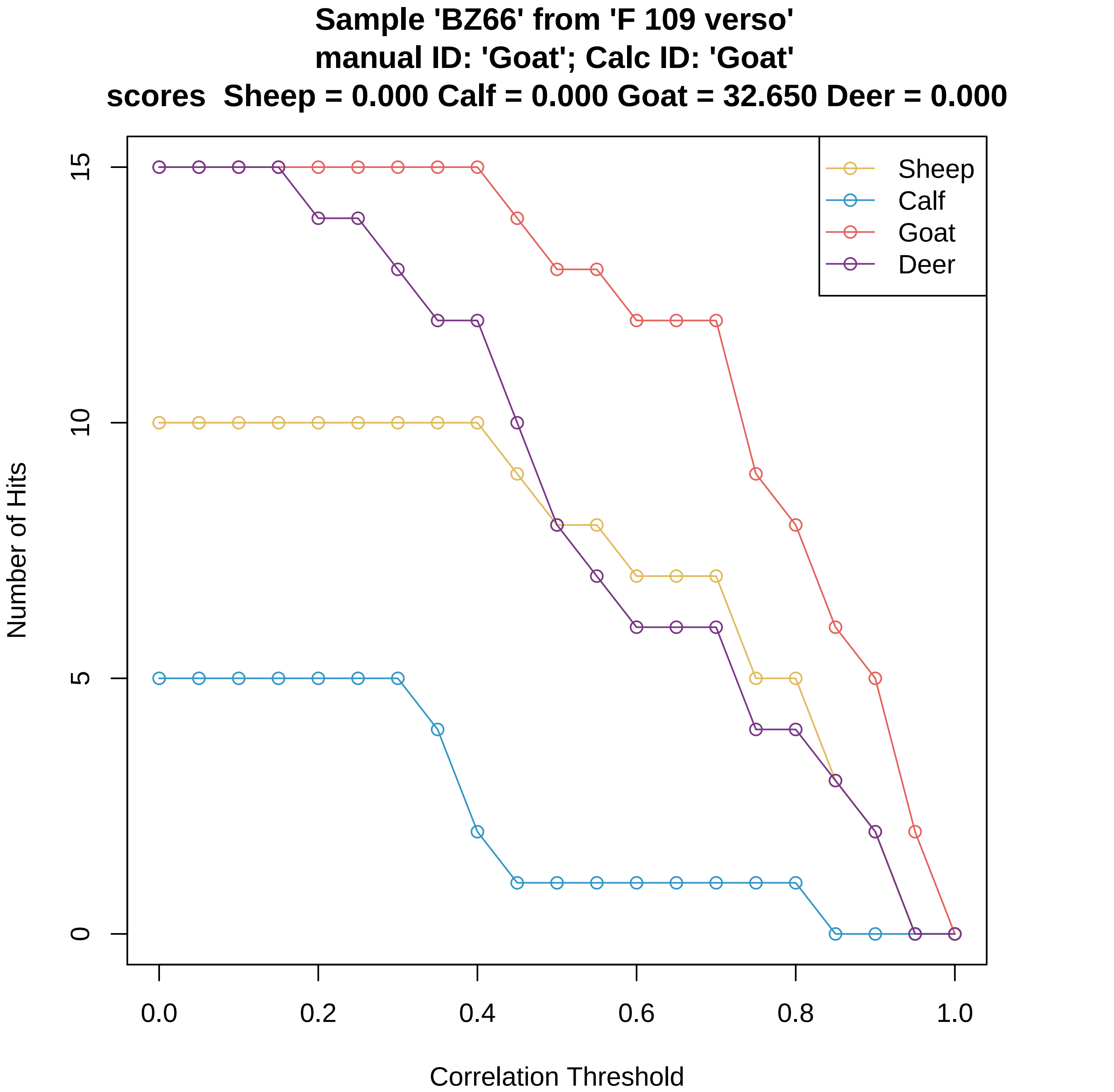
Sample 'BZ66' from 'F 109 verso'
manual ID: 'Goat'; Calc ID: 'Goat'
scores Sheep = 0.000 Calf = 0.000 Goat = 32.650 Deer = 0.000
Sheep
Calf
Goat
Deer
Number of Hits
0
5
10
15
0.0
0.2
0.4
0.6
0.8
1.0
Correlation Threshold

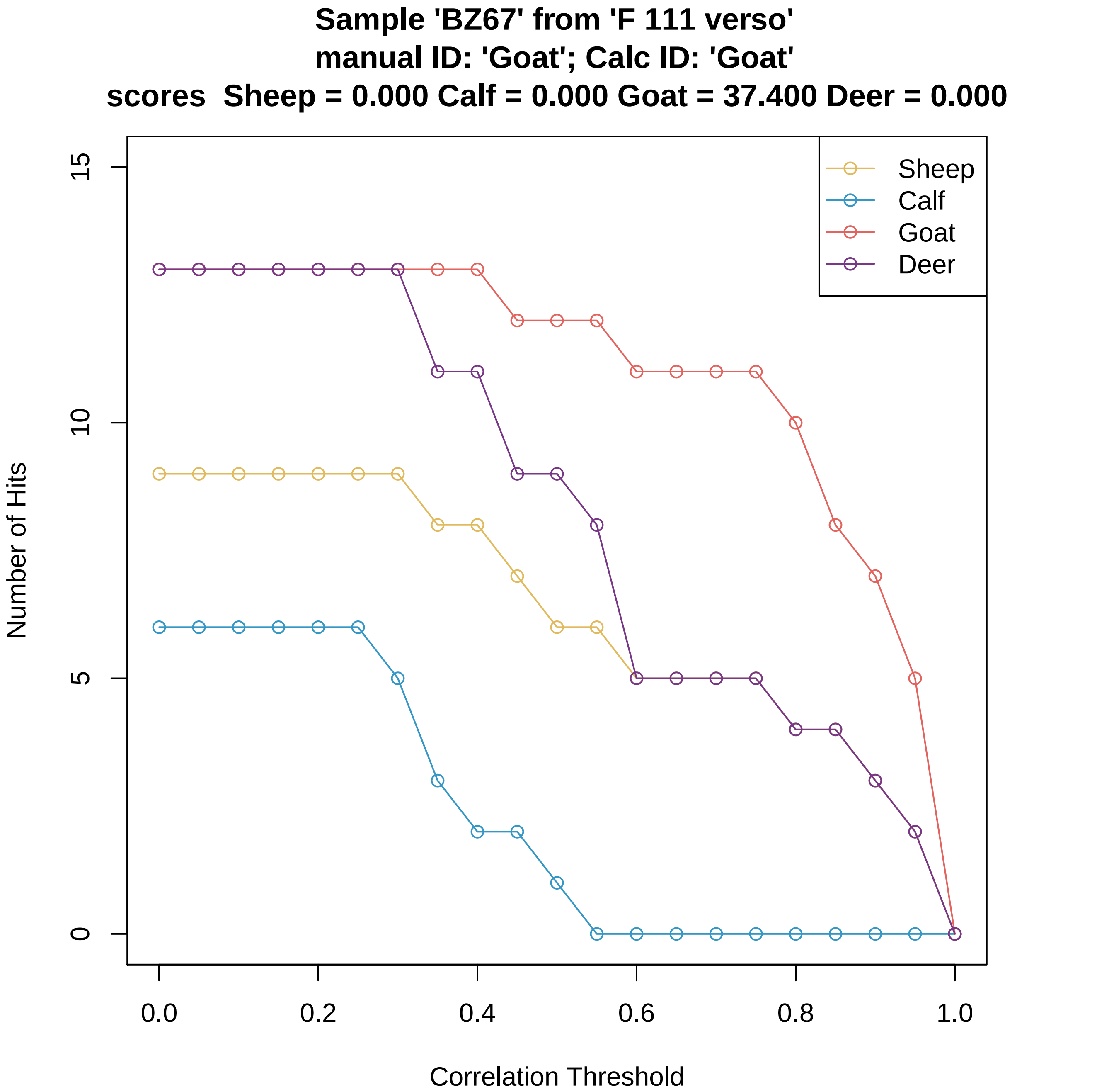
Sample 'BZ67' from 'F 111 verso'
manual ID: 'Goat'; Calc ID: 'Goat'
scores Sheep = 0.000 Calf = 0.000 Goat = 37.400 Deer = 0.000
Sheep
Calf
Goat
Deer
Number of Hits
0
5
10
15
0.0
0.2
0.4
0.6
0.8
1.0
Correlation Threshold

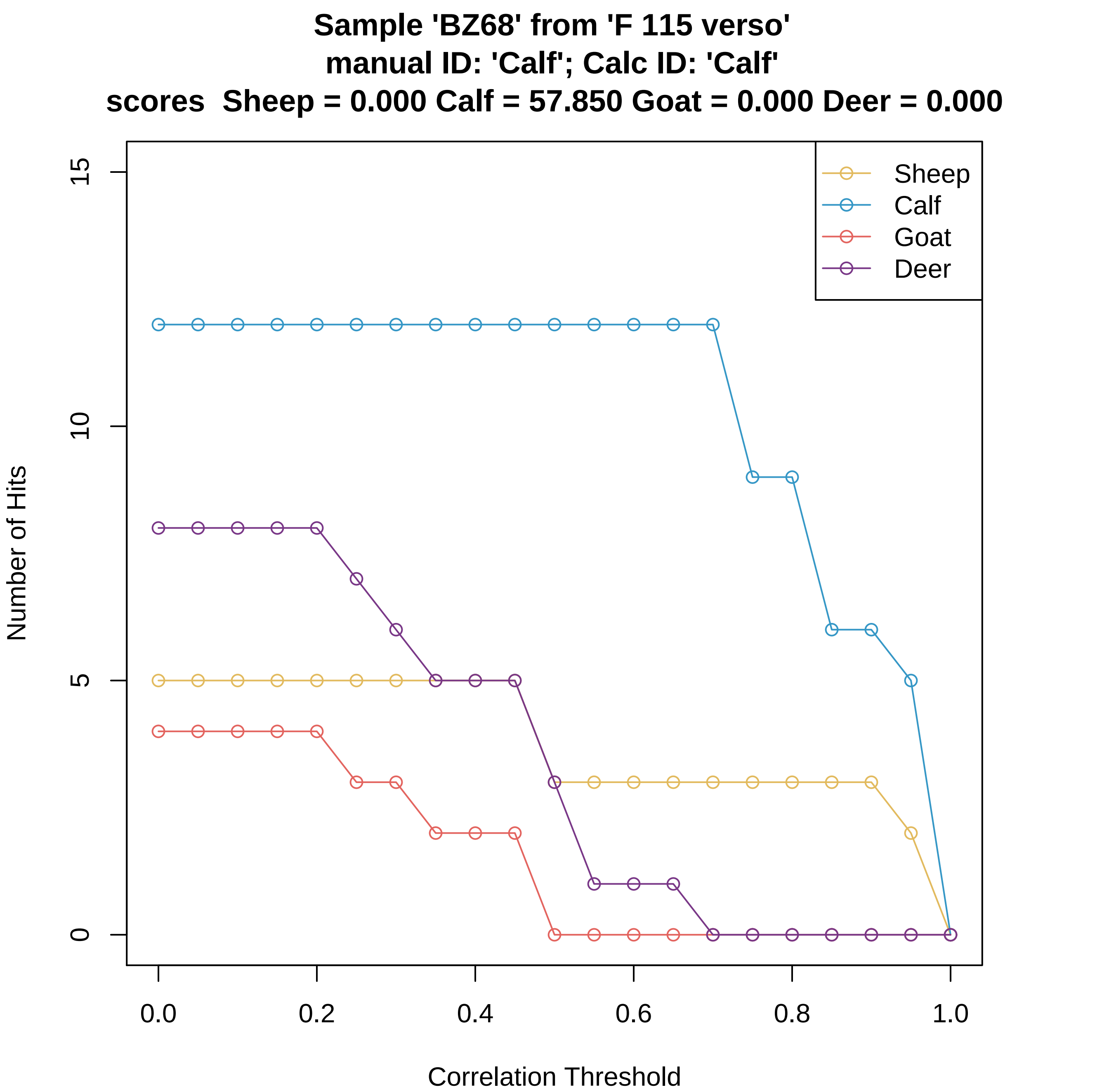
Sample 'BZ68' from 'F 115 verso'
manual ID: 'Calf'; Calc ID: 'Calf'
scores Sheep = 0.000 Calf = 57.850 Goat = 0.000 Deer = 0.000
Sheep
Calf
Goat
Deer
Number of Hits
0
5
10
15
0.0
0.2
0.4
0.6
0.8
1.0
Correlation Threshold

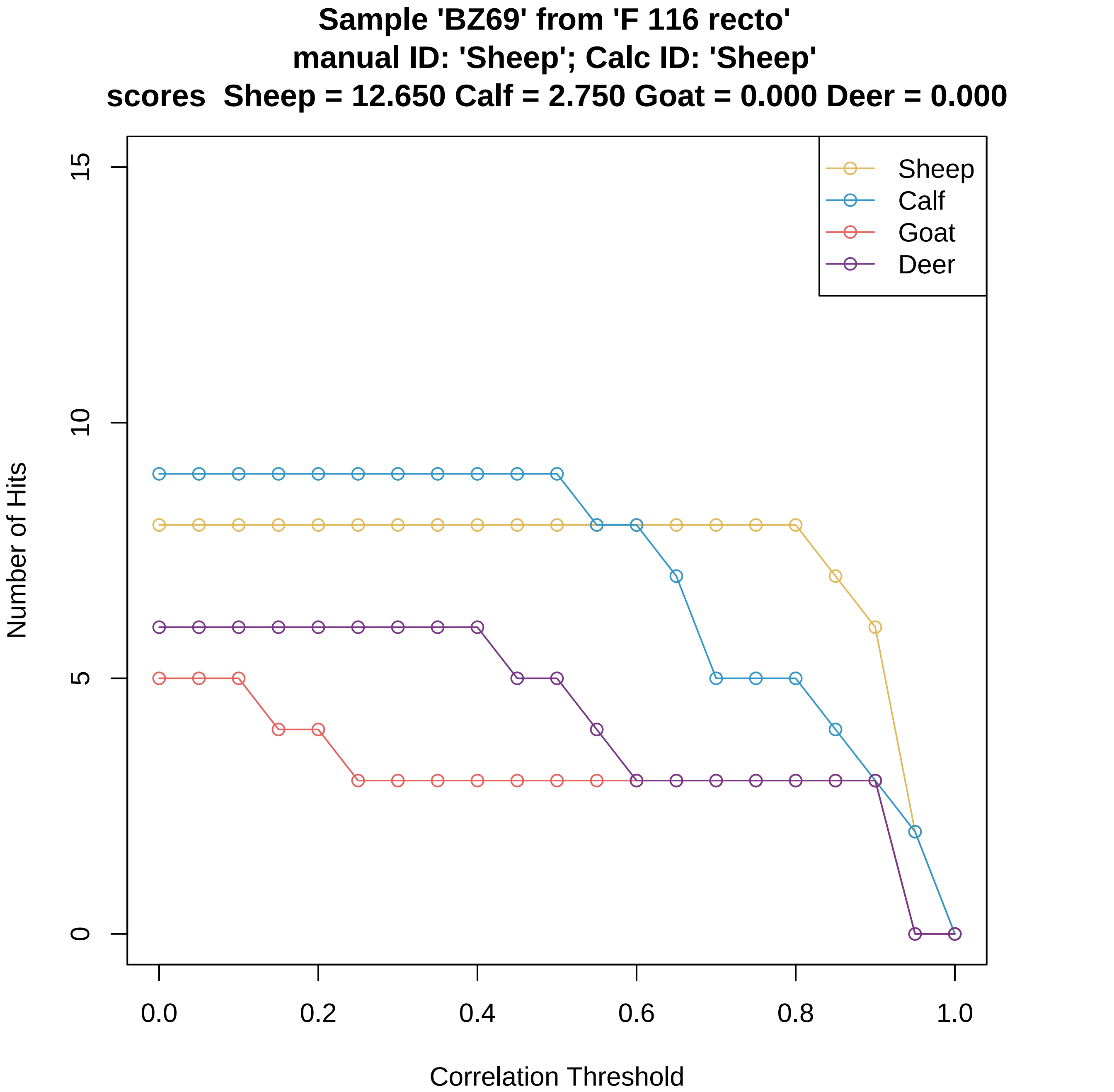

Sample 'BZ69' from 'F 116 recto'
manual ID: 'Sheep'; Calc ID: 'Sheep'
scores Sheep = 12.650 Calf = 2.750 Goat = 0.000 Deer = 0.000
Sheep
Calf
Goat
Deer
Number of Hits
0
5
10
15
0.0
0.2
0.4
0.6
0.8
1.0
Correlation Threshold

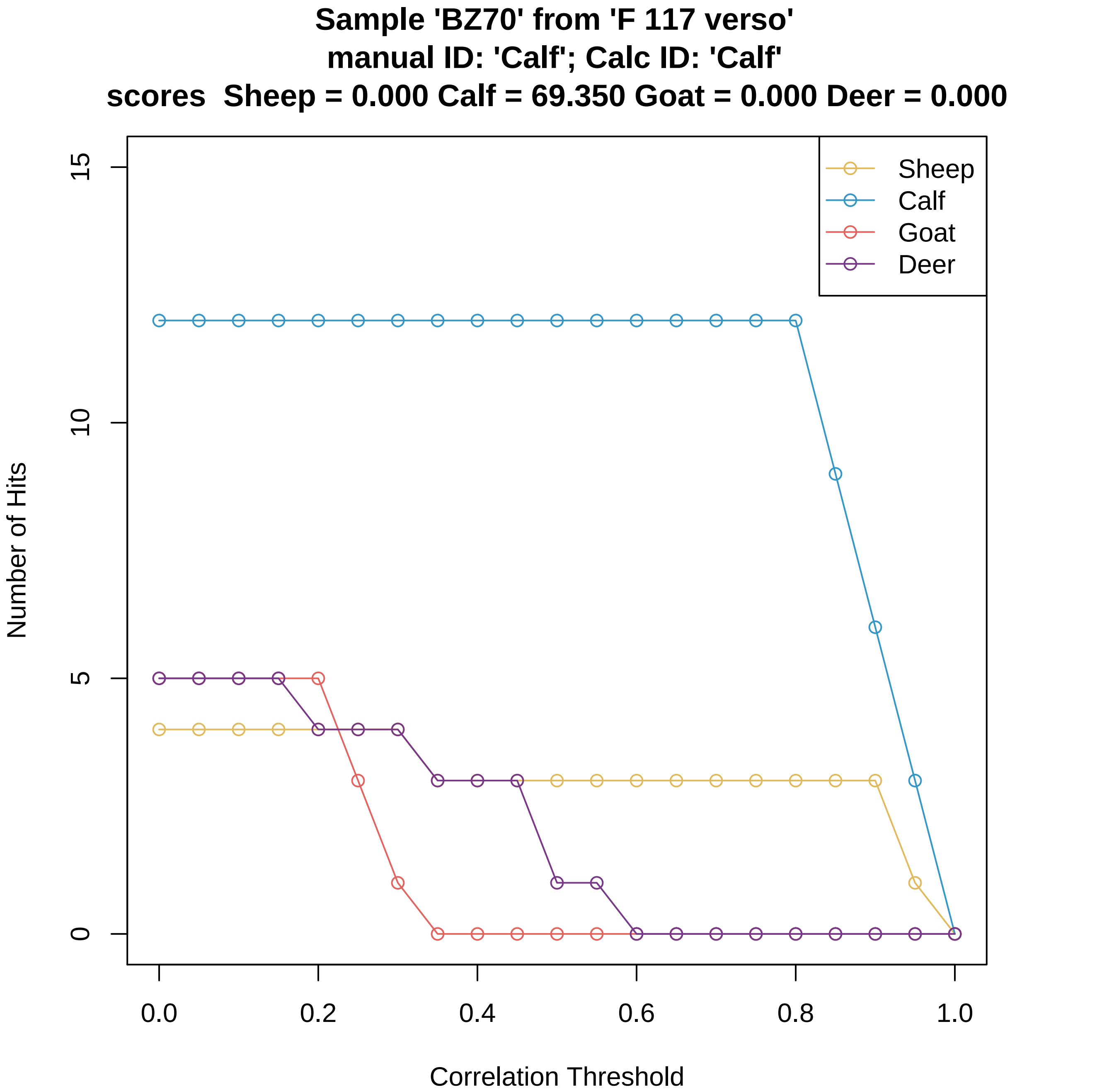

Sample 'BZ70' from 'F 117 verso'
manual ID: 'Calf'; Calc ID: 'Calf'
scores Sheep = 0.000 Calf = 69.350 Goat = 0.000 Deer = 0.000
Sheep
Calf
Goat
Deer
Number of Hits
0
5
10
15
0.0
0.2
0.4
0.6
0.8
1.0
Correlation Threshold

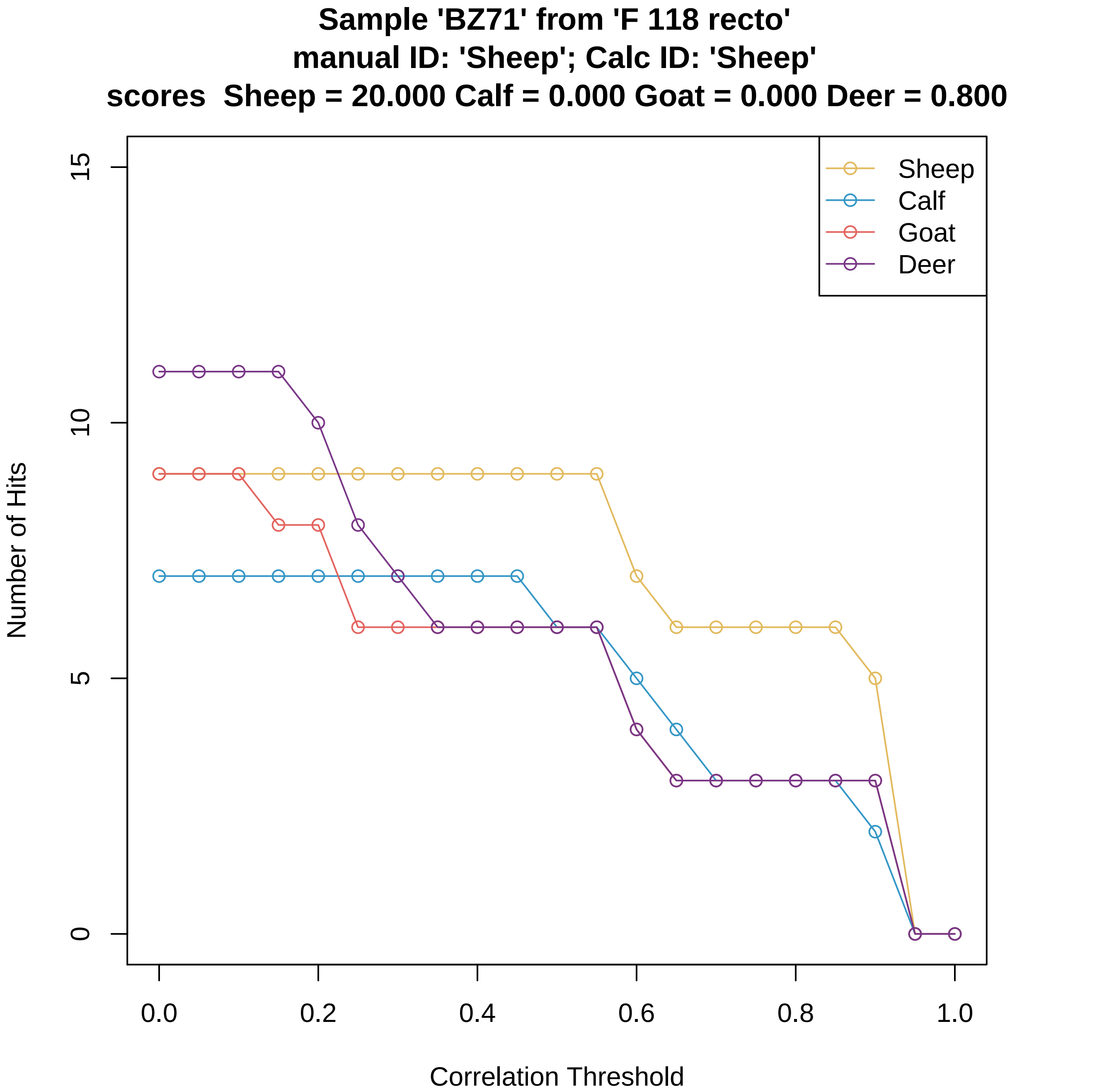

Sample 'BZ71' from 'F 118 recto'
manual ID: 'Sheep'; Calc ID: 'Sheep'
scores Sheep = 20.000 Calf = 0.000 Goat = 0.000 Deer = 0.800
Sheep
Calf
Goat
Deer
Number of Hits
0
5
10
15
0.0
0.2
0.4
0.6
0.8
1.0
Correlation Threshold

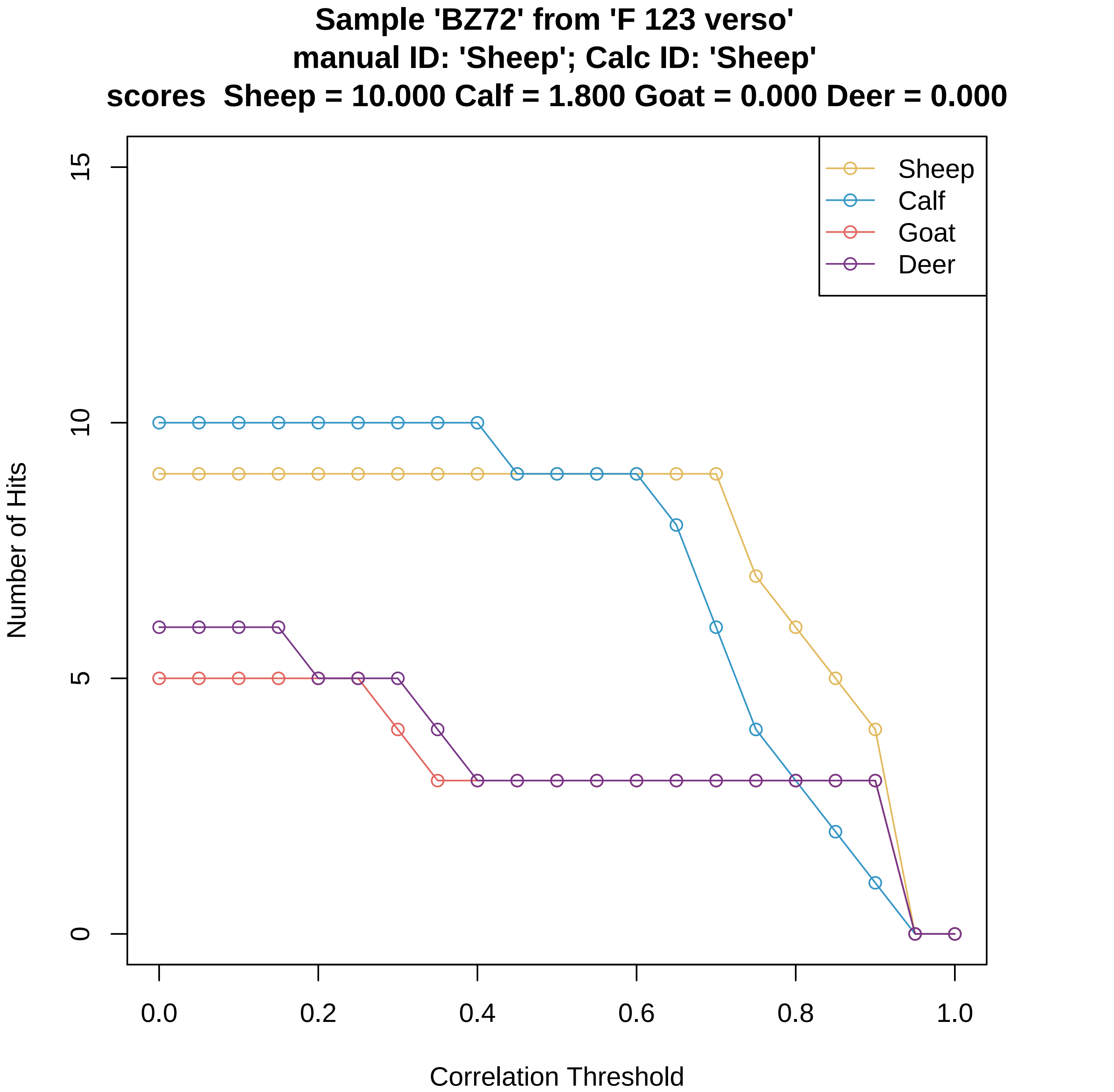
Sample 'BZ72' from 'F 123 verso'
manual ID: 'Sheep'; Calc ID: 'Sheep'
scores Sheep = 10.000 Calf = 1.800 Goat = 0.000 Deer = 0.000
Sheep
Calf
Goat
Deer
0
5
10
15
Number of Hits
0.0
0.2
0.4
0.6
0.8
1.0
Correlation Threshold

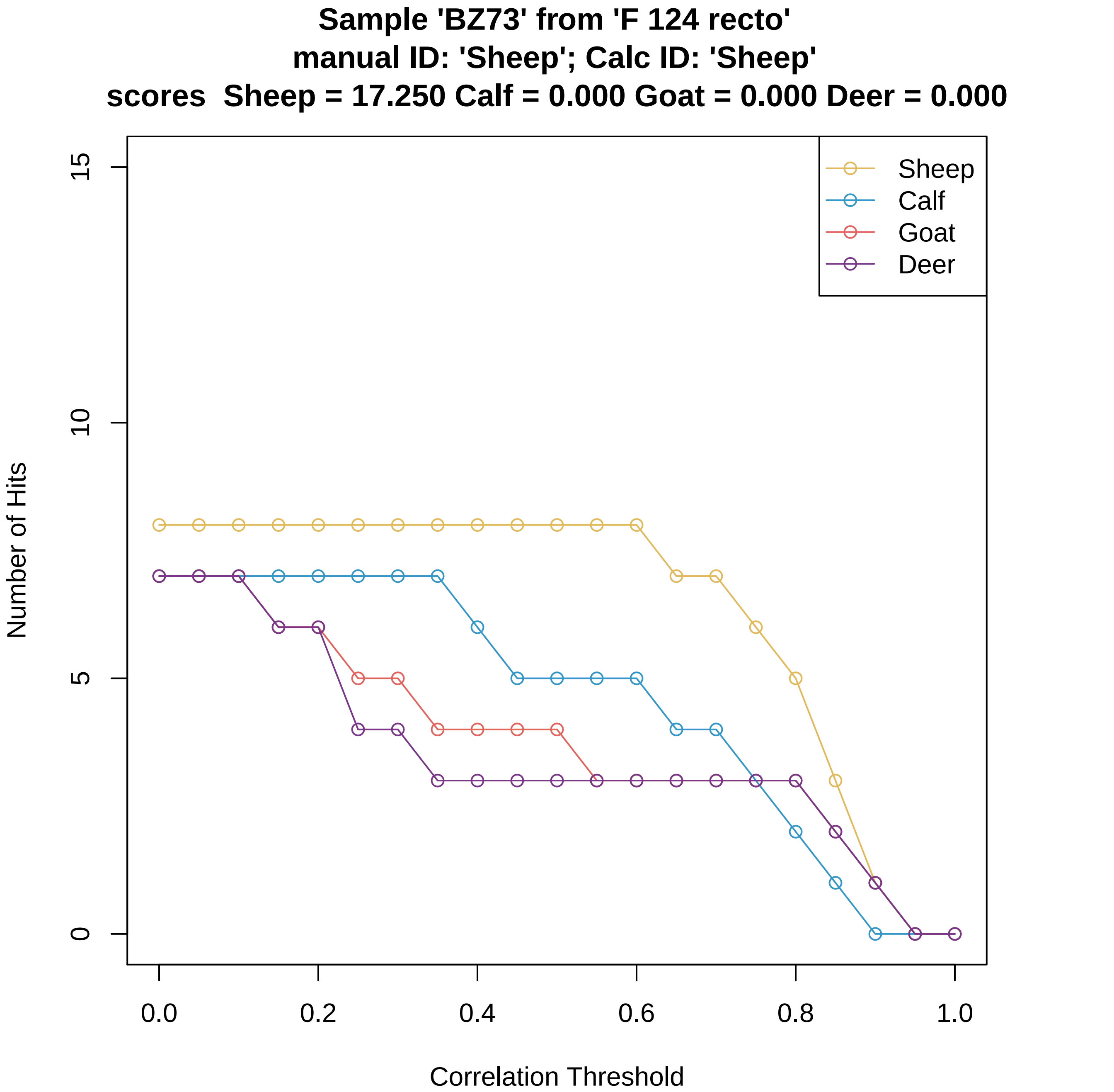
Sample 'BZ73' from 'F 124 recto'
manual ID: 'Sheep'; Calc ID: 'Sheep'
scores Sheep = 17.250 Calf = 0.000 Goat = 0.000 Deer = 0.000
Sheep
Calf
Goat
Deer
Number of Hits
0
5
10
15
0.0
0.2
0.4
0.6
0.8
1.0
Correlation Threshold

Sample 'BZ74' from 'F 125 verso'
manual ID: 'Sheep'; Calc ID: 'Sheep'
scores Sheep = 12.200 Calf = 0.000 Goat = 0.000 Deer = 0.000

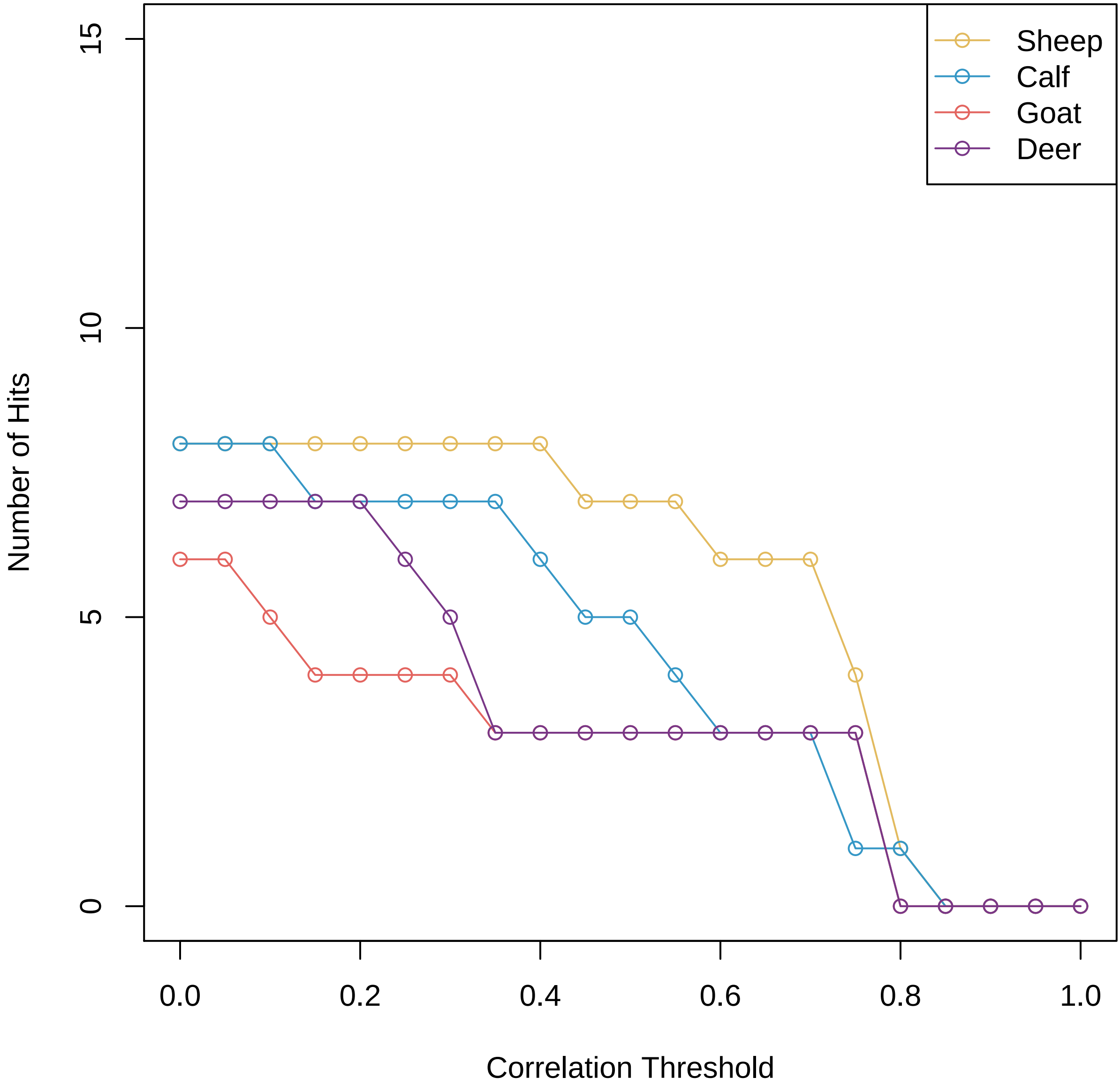

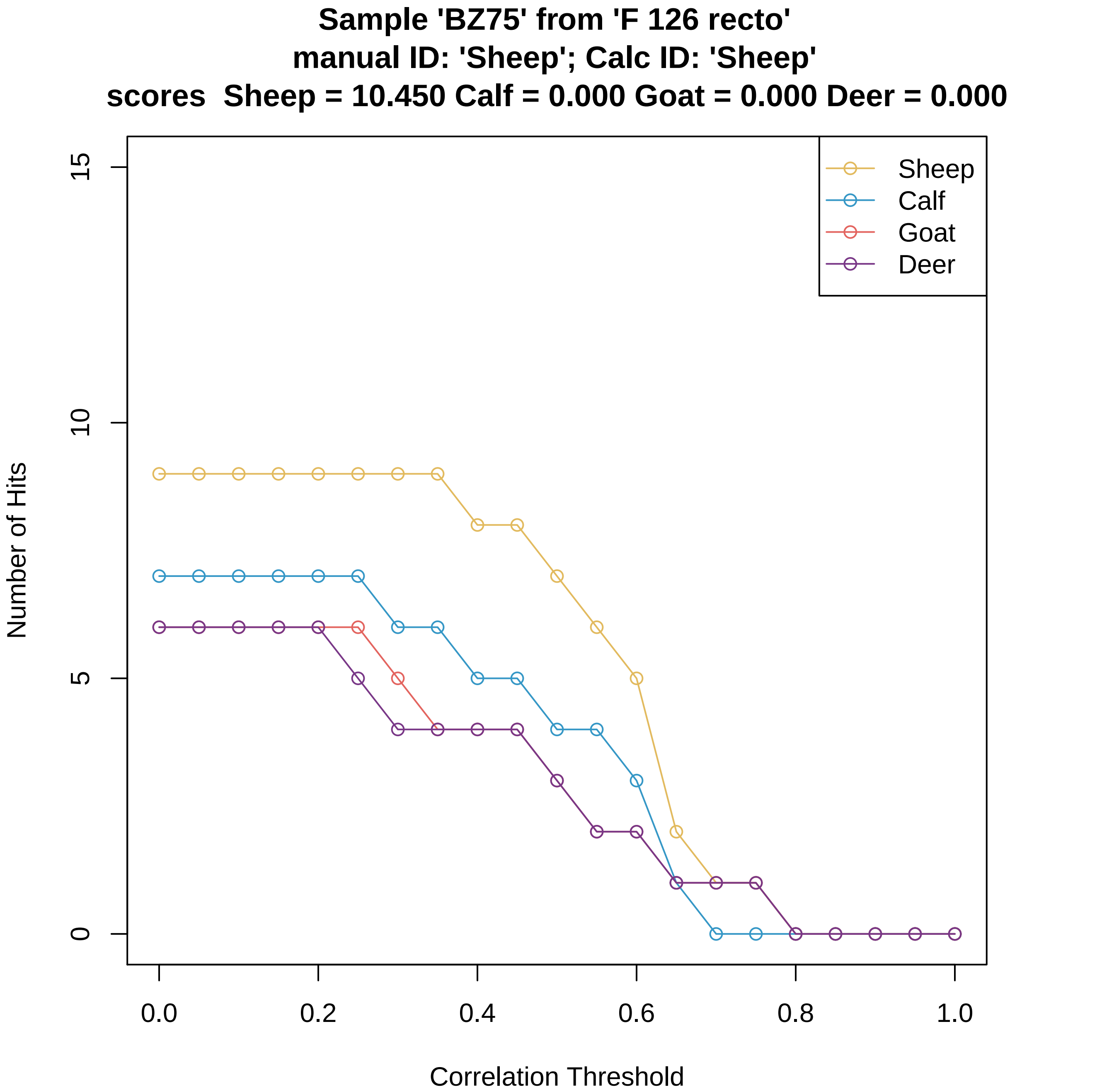

Sample 'BZ75' from 'F 126 recto'
manual ID: 'Sheep'; Calc ID: 'Sheep'
scores Sheep = 10.450 Calf = 0.000 Goat = 0.000 Deer = 0.000
Sheep
Calf
Goat
Deer
0
5
10
15
0.0
0.2
0.4
0.6
0.8
1.0
Number of Hits
Correlation Threshold

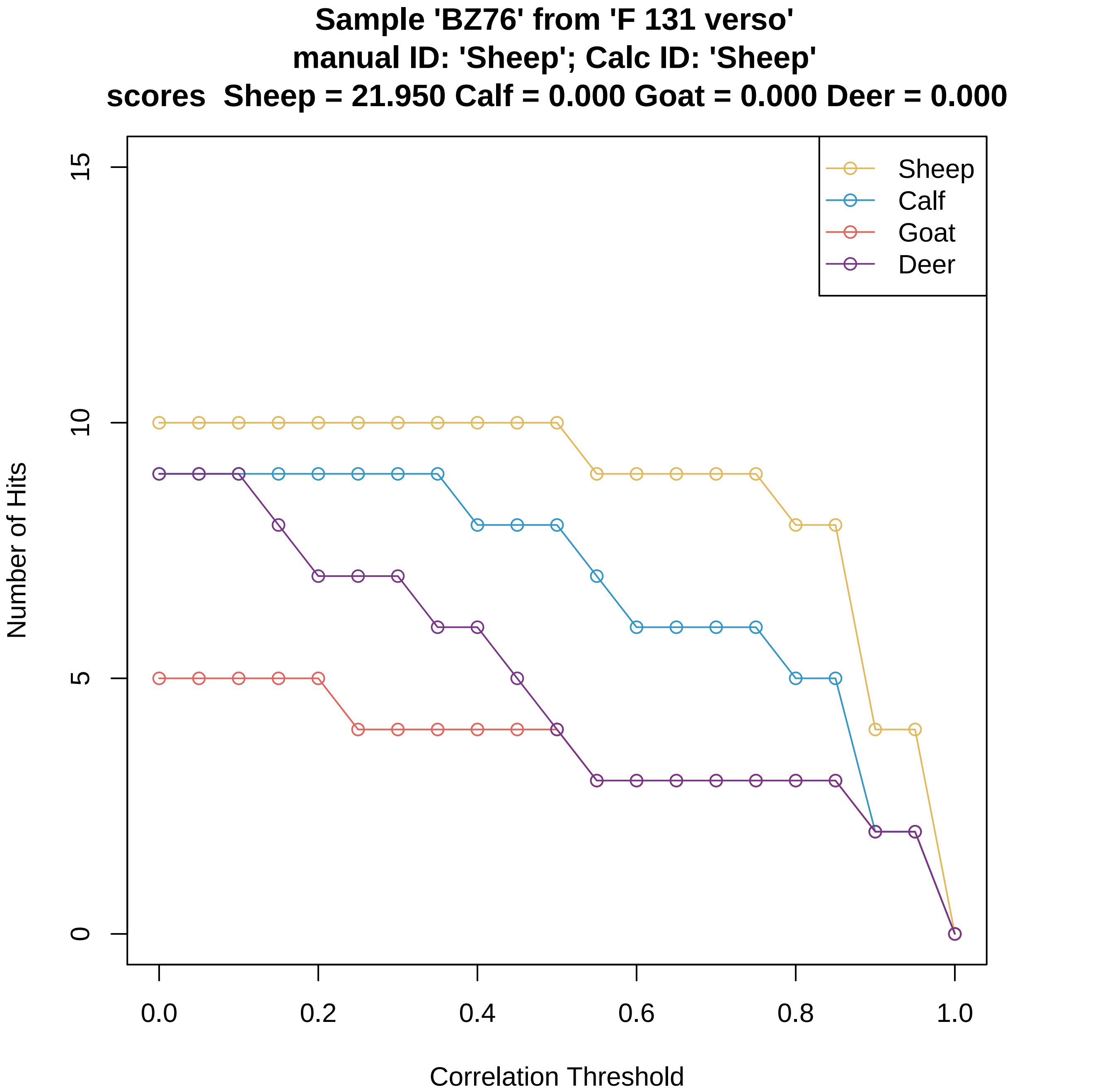

Sample 'BZ76' from 'F 131 verso'
manual ID: 'Sheep'; Calc ID: 'Sheep'
scores Sheep = 21.950 Calf = 0.000 Goat = 0.000 Deer = 0.000
Sheep
Calf
Goat
Deer
Number of Hits
0
5
10
15
0.0
0.2
0.4
0.6
0.8
1.0
Correlation Threshold

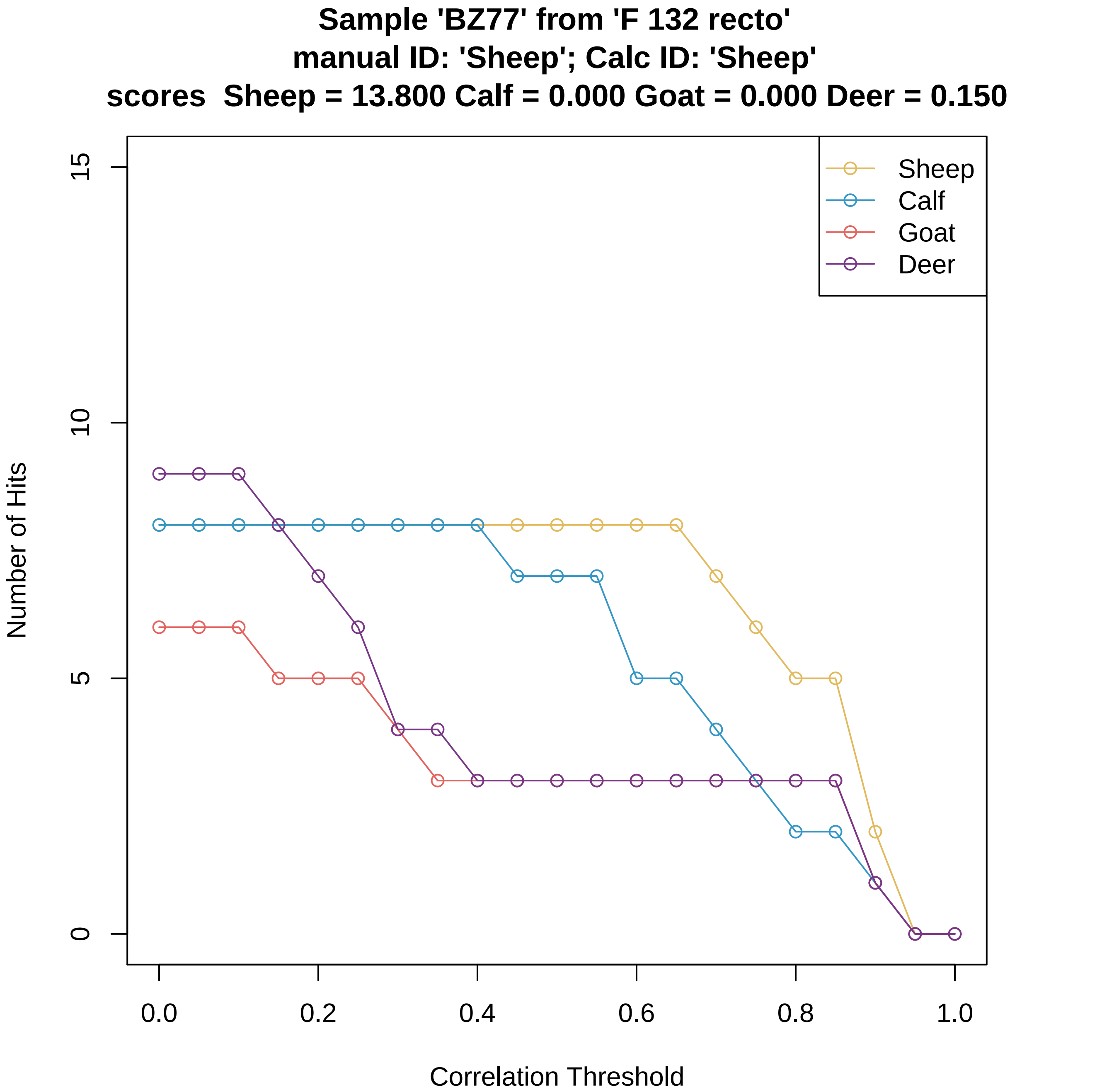
Sample 'BZ77' from 'F 132 recto'
manual ID: 'Sheep'; Calc ID: 'Sheep'
scores Sheep = 13.800 Calf = 0.000 Goat = 0.000 Deer = 0.150
Sheep
Calf
Goat
Deer
Number of Hits
Correlation Threshold
0
5
10
15
0.0
0.2
0.4
0.6
0.8
1.0

Sample 'BZ78' from 'F 133 verso'
manual ID: 'Sheep'; Calc ID: 'Sheep'
scores Sheep = 33.750 Calf = 0.000 Goat = 0.000 Deer = 0.000

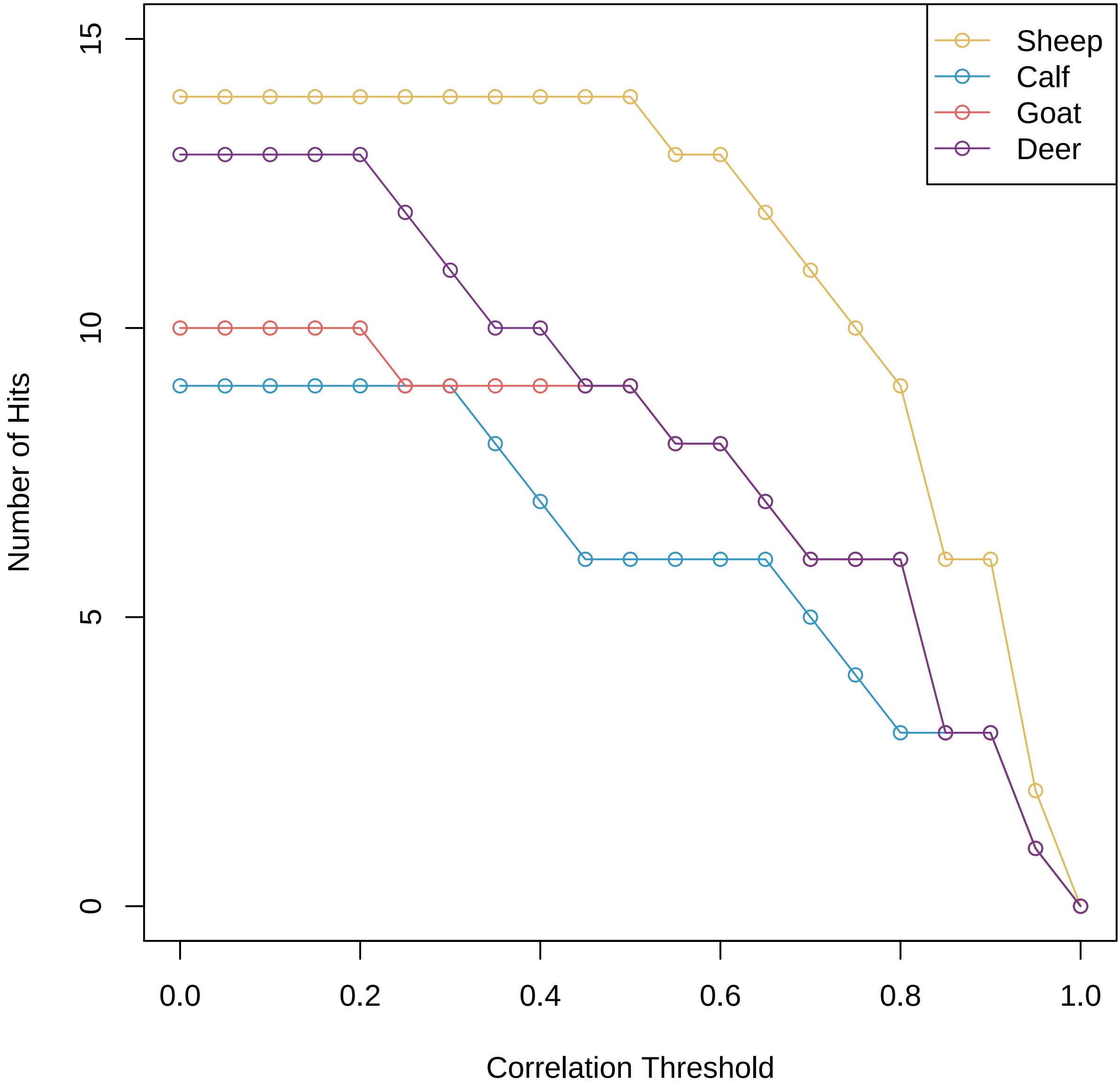

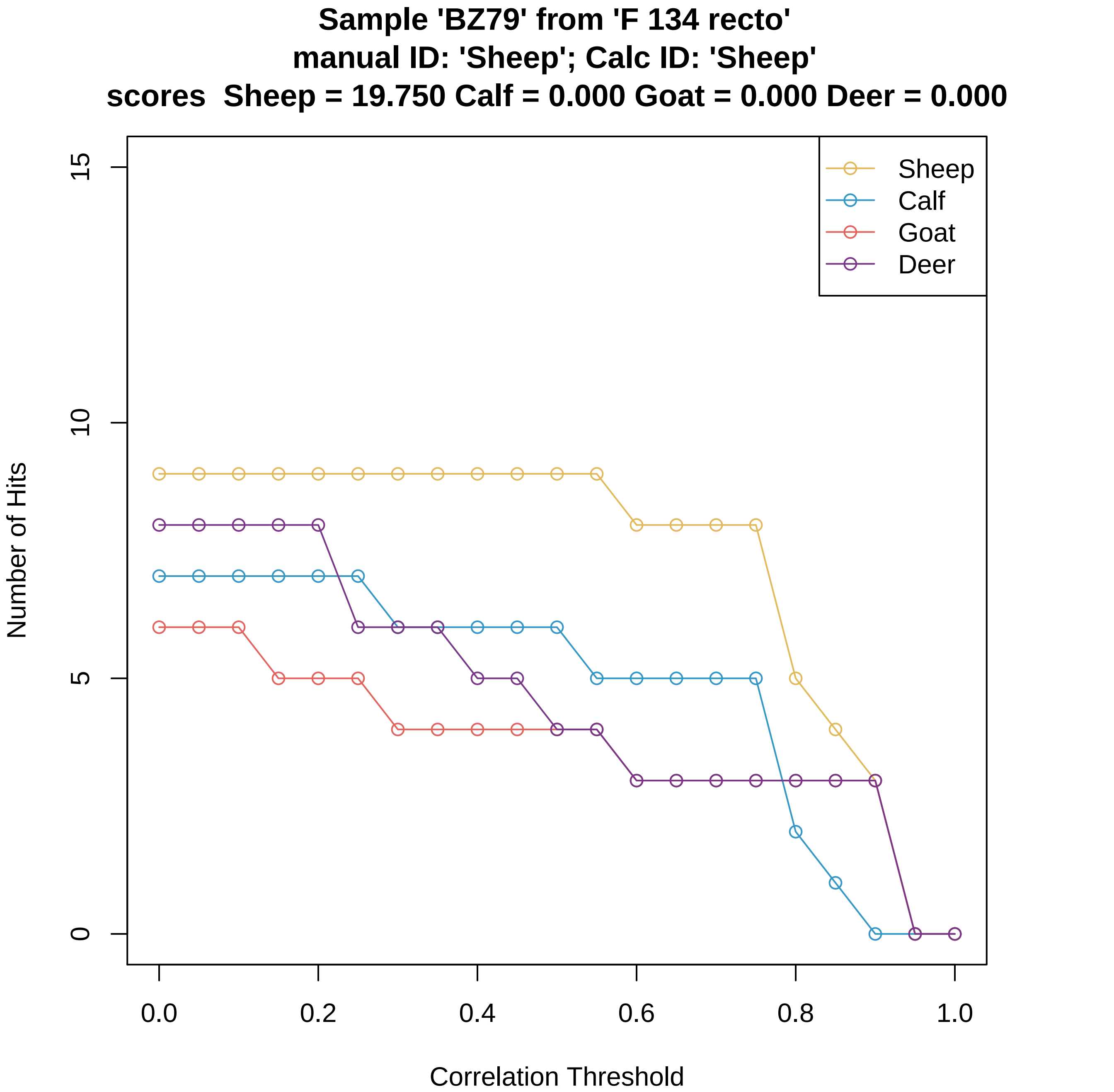
Sample 'BZ79' from 'F 134 recto'
manual ID: 'Sheep'; Calc ID: 'Sheep'
scores Sheep = 19.750 Calf = 0.000 Goat = 0.000 Deer = 0.000
Sheep
Calf
Goat
Deer
Number of Hits
0
5
10
15
0.0
0.2
0.4
0.6
0.8
1.0
Correlation Threshold

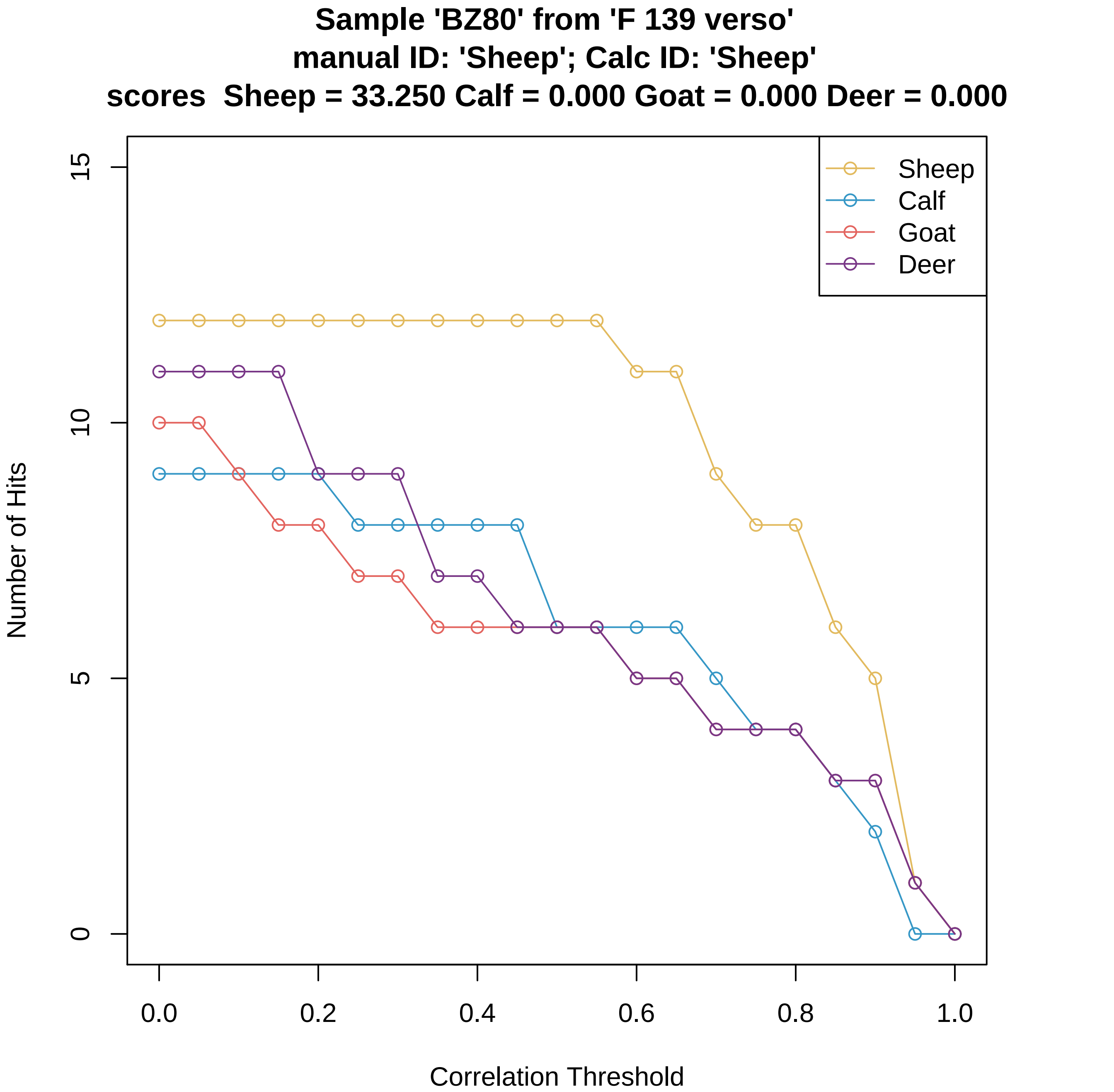

Sample 'BZ80' from 'F 139 verso'
manual ID: 'Sheep'; Calc ID: 'Sheep'
scores Sheep = 33.250 Calf = 0.000 Goat = 0.000 Deer = 0.000
Sheep
Calf
Goat
Deer
Number of Hits
0
5
10
15
0.0
0.2
0.4
0.6
0.8
1.0
Correlation Threshold

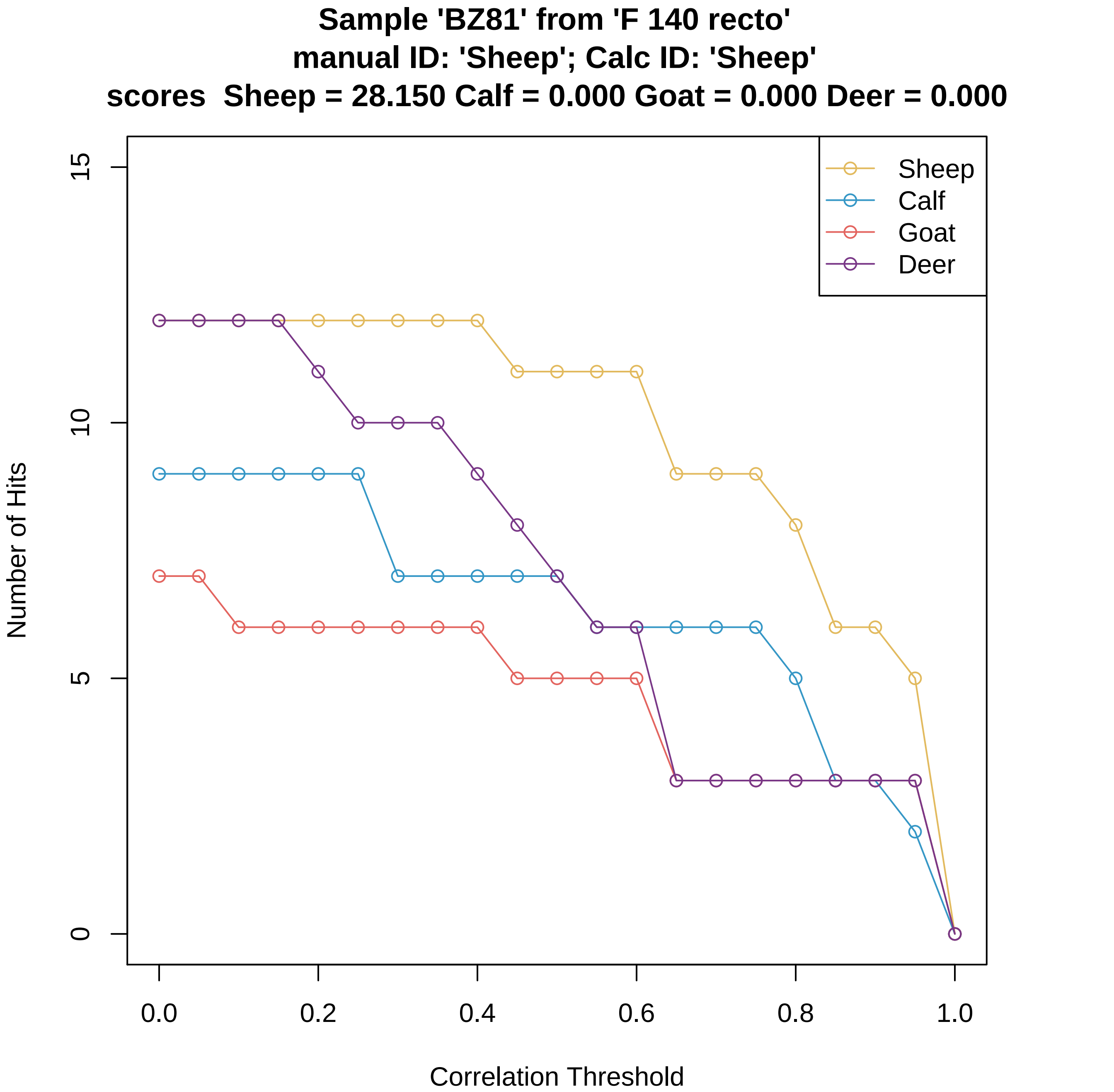
Sample 'BZ81' from 'F 140 recto'
manual ID: 'Sheep'; Calc ID: 'Sheep'
scores Sheep = 28.150 Calf = 0.000 Goat = 0.000 Deer = 0.000
Number of Hits
Correlation Threshold
0
5
10
15
0.0
0.2
0.4
0.6
0.8
1.0
Sheep
Calf
Goat
Deer

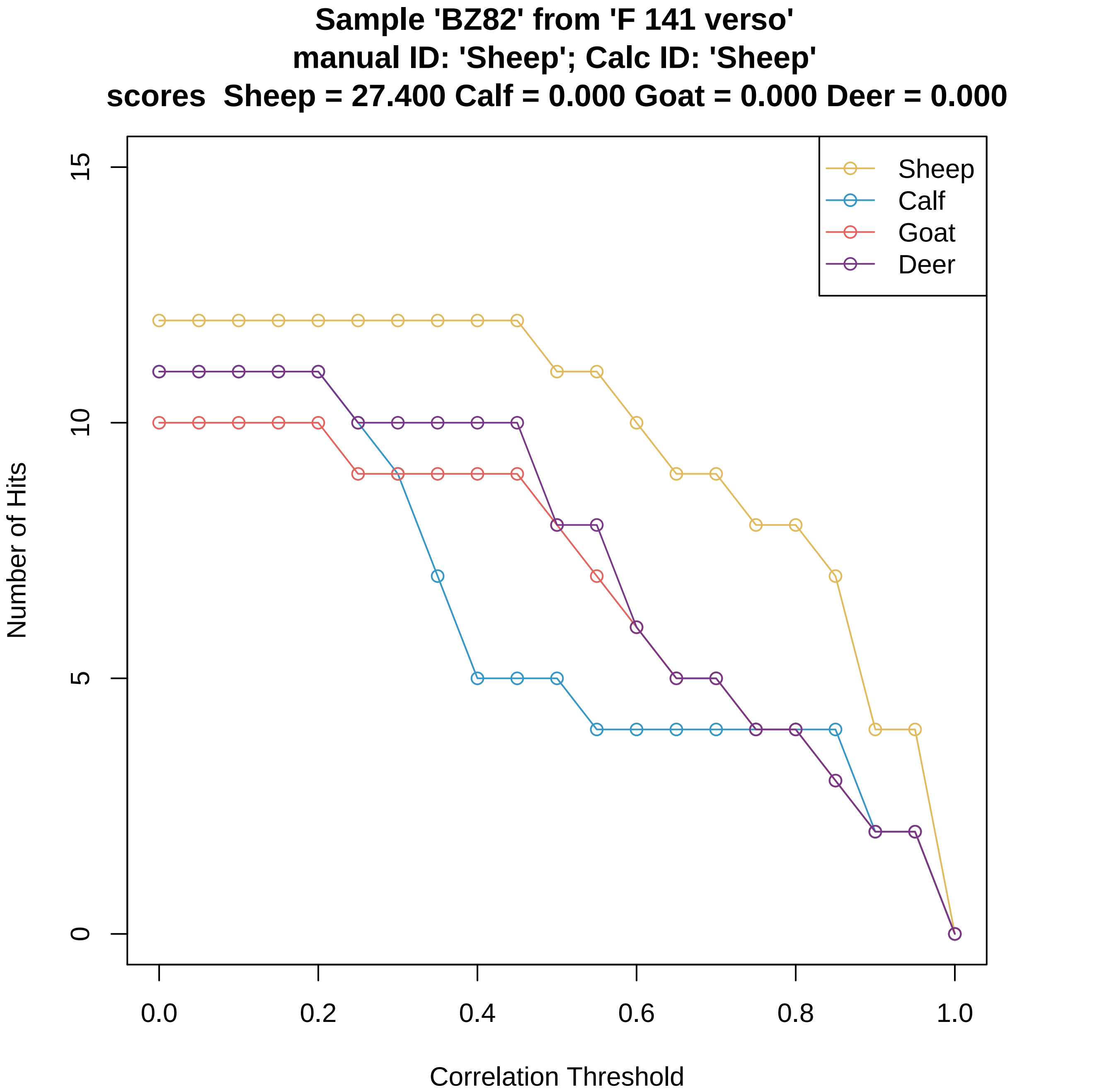
Sample 'BZ82' from 'F 141 verso'
manual ID: 'Sheep'; Calc ID: 'Sheep'
scores Sheep = 27.400 Calf = 0.000 Goat = 0.000 Deer = 0.000
Sheep
Calf
Goat
Deer
Number of Hits
0
5
10
15
0.0
0.2
0.4
0.6
0.8
1.0
Correlation Threshold

Sample 'BZ83' from 'F 142 recto'
manual ID: 'Sheep'; Calc ID: 'Sheep'
scores Sheep = 22.250 Calf = 0.000 Goat = 0.000 Deer = 0.000

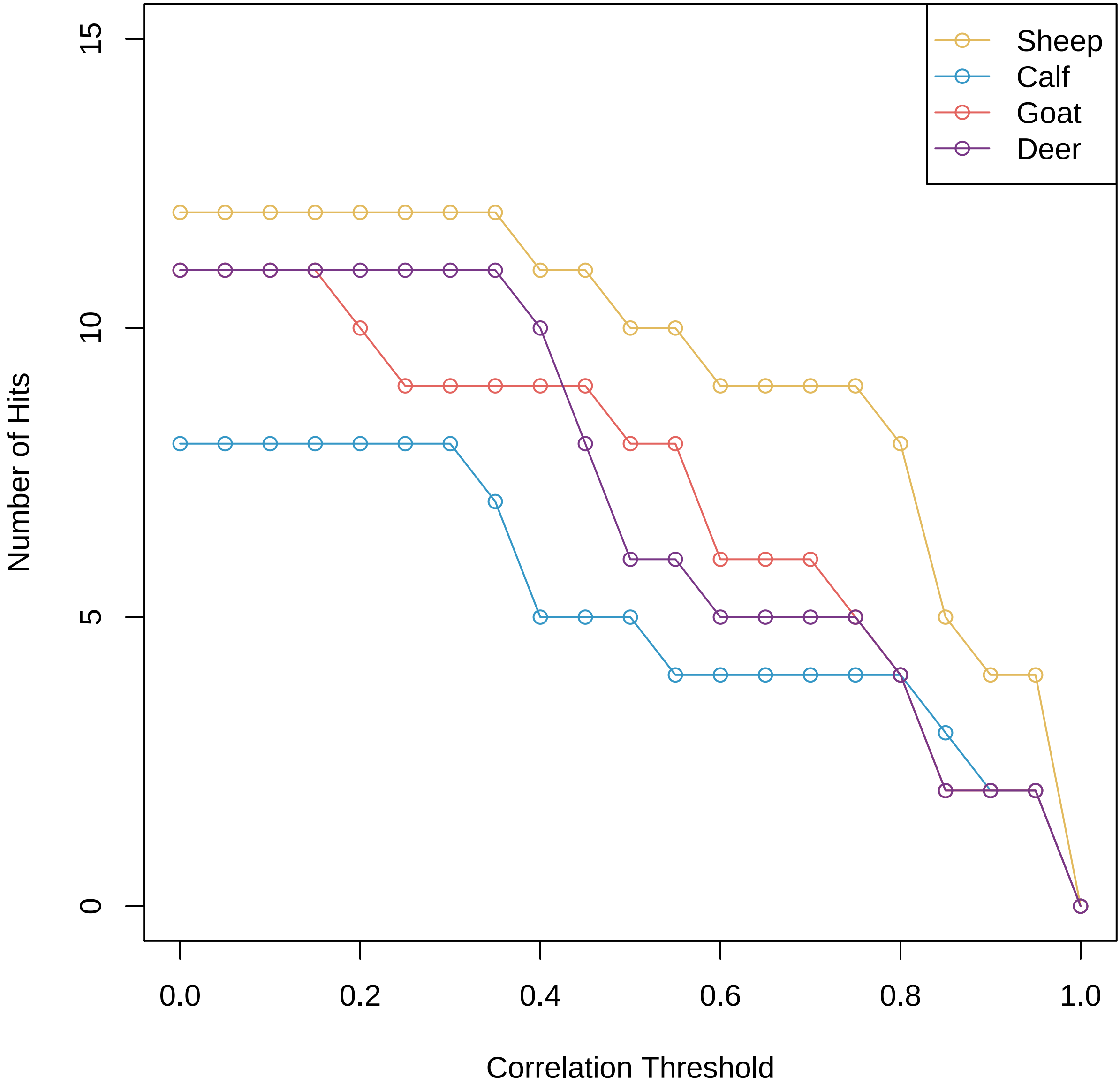

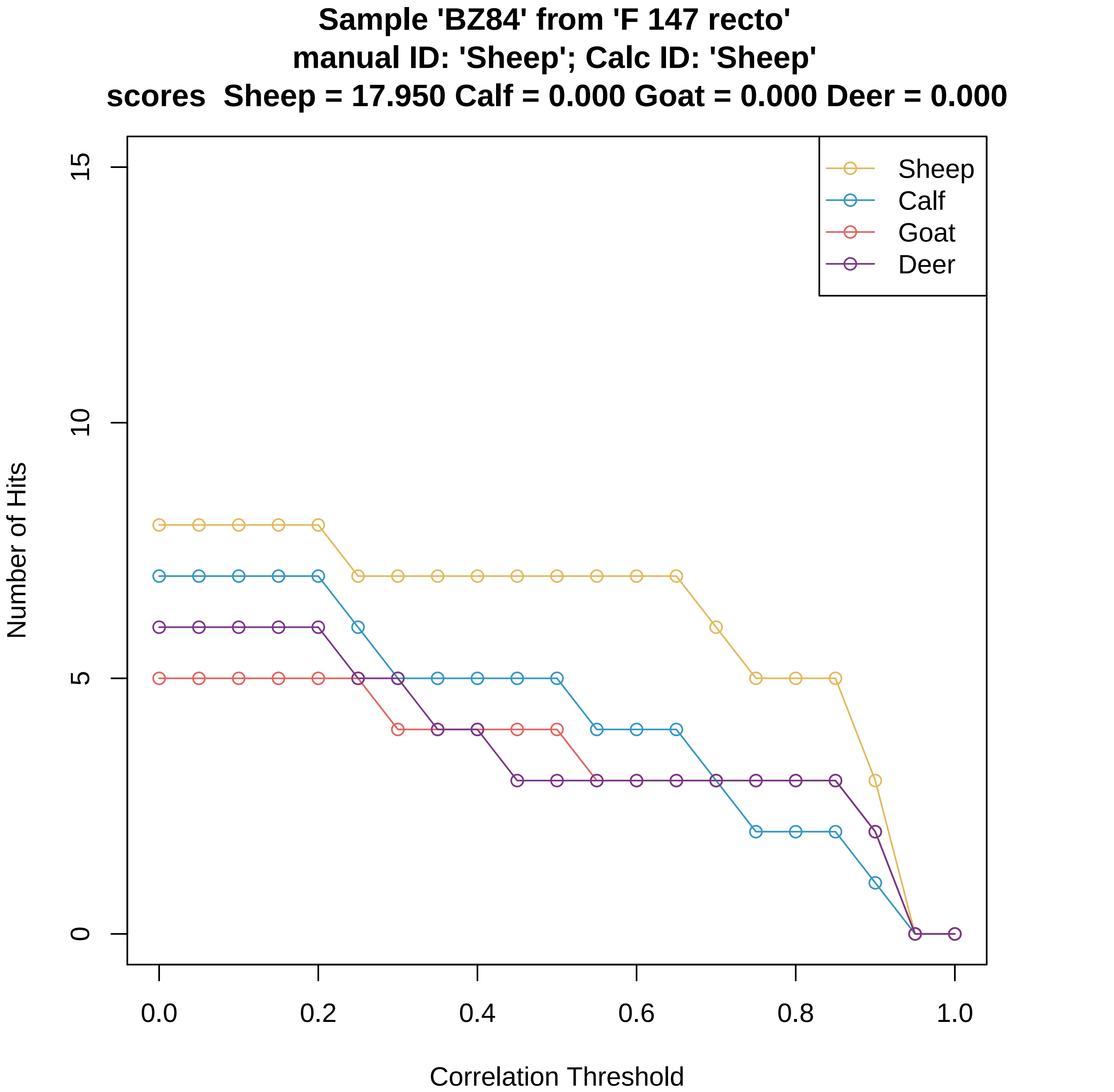

Sample 'BZ84' from 'F 147 recto'
manual ID: 'Sheep'; Calc ID: 'Sheep'
scores Sheep = 17.950 Calf = 0.000 Goat = 0.000 Deer = 0.000
Sheep
Calf
Goat
Deer
0
5
10
15
Number of Hits
0.0
0.2
0.4
0.6
0.8
1.0
Correlation Threshold

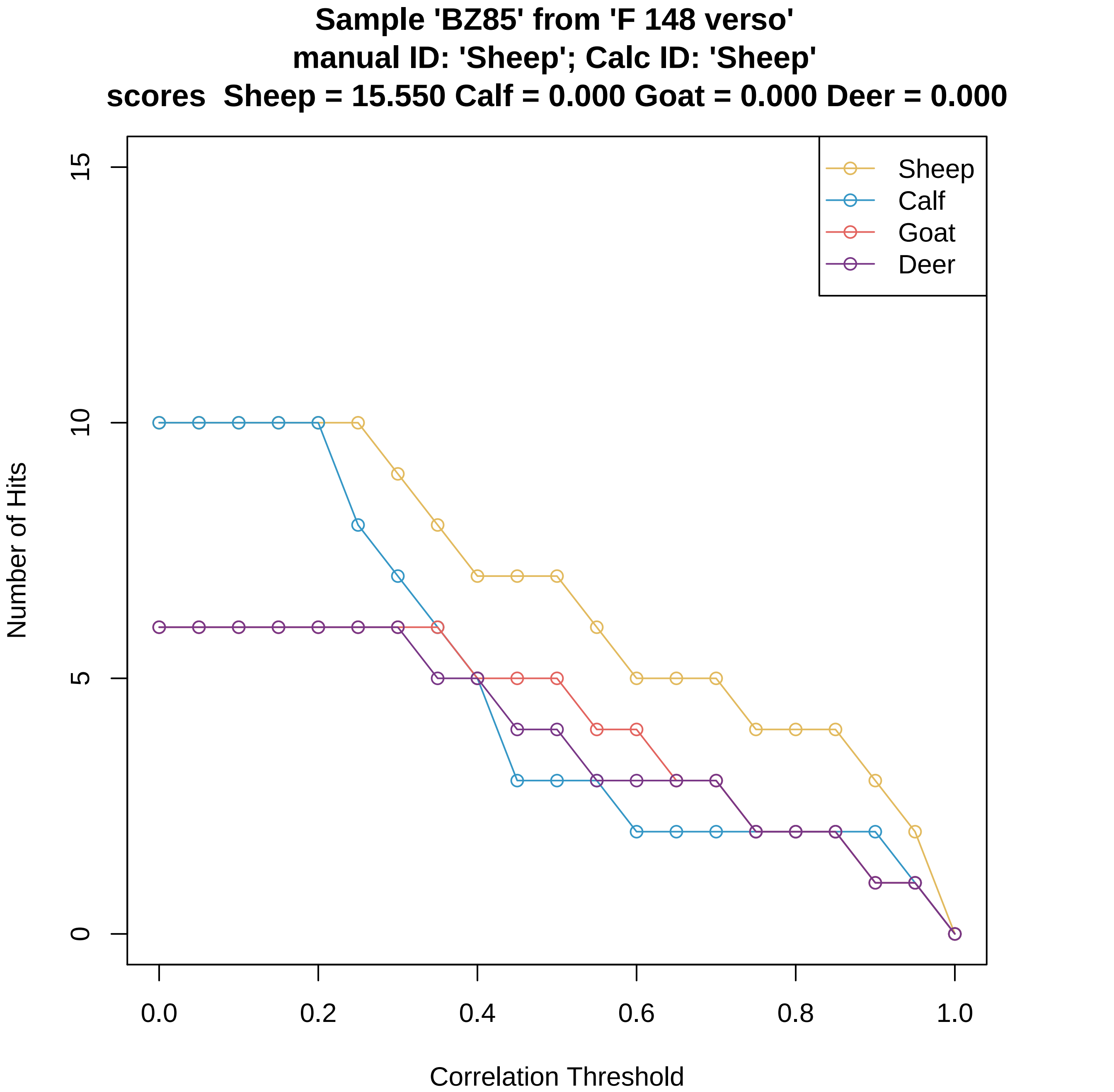

Sample 'BZ85' from 'F 148 verso'
manual ID: 'Sheep'; Calc ID: 'Sheep'
scores Sheep = 15.550 Calf = 0.000 Goat = 0.000 Deer = 0.000
Sheep
Calf
Goat
Deer
Number of Hits
0
5
10
15
0.0
0.2
0.4
0.6
0.8
1.0
Correlation Threshold

Sample 'BZ86' from 'F 149 recto'
manual ID: 'Sheep'; Calc ID: 'Sheep'
scores Sheep = 15.550 Calf = 0.000 Goat = 0.000 Deer = 0.000

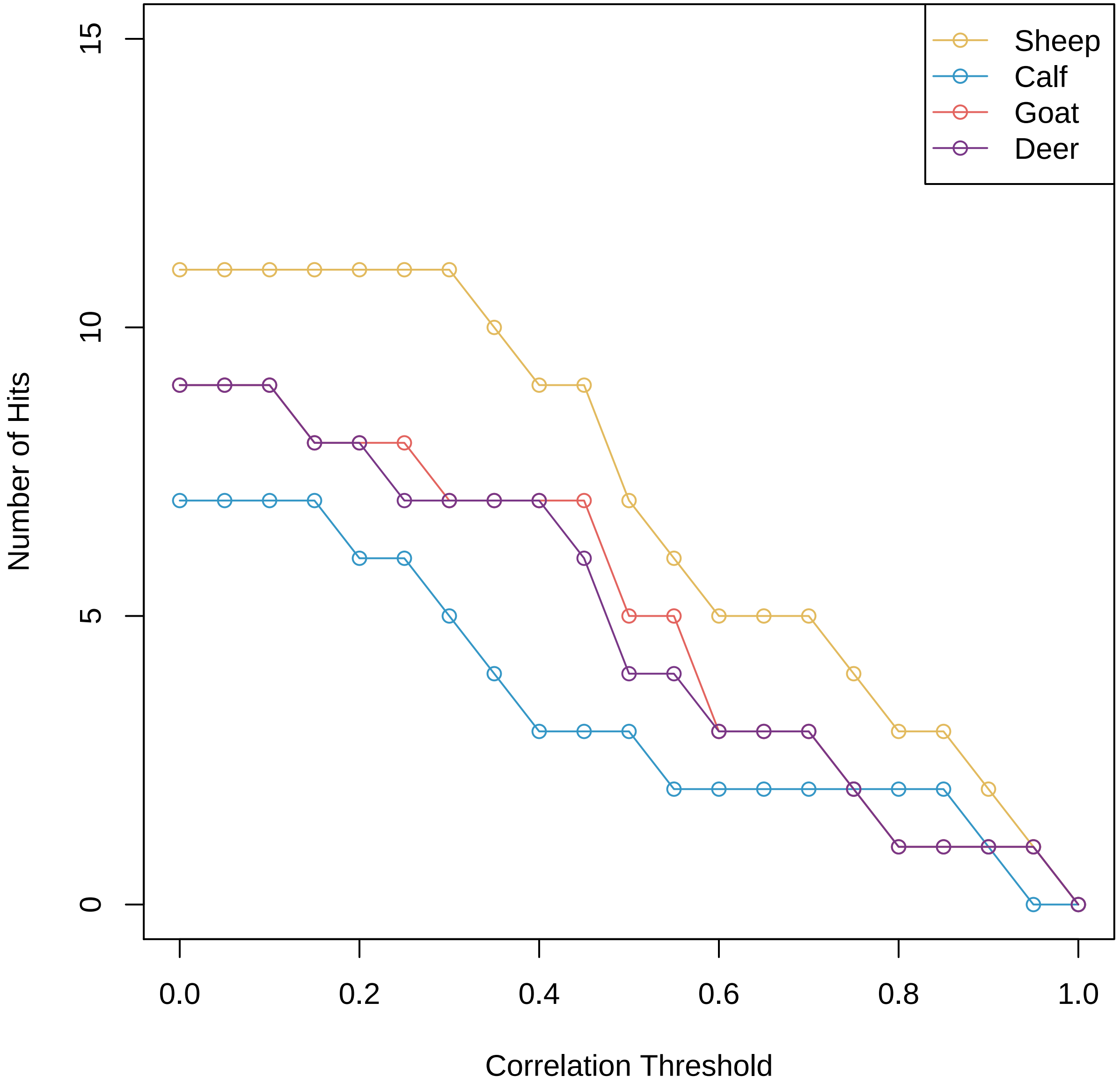

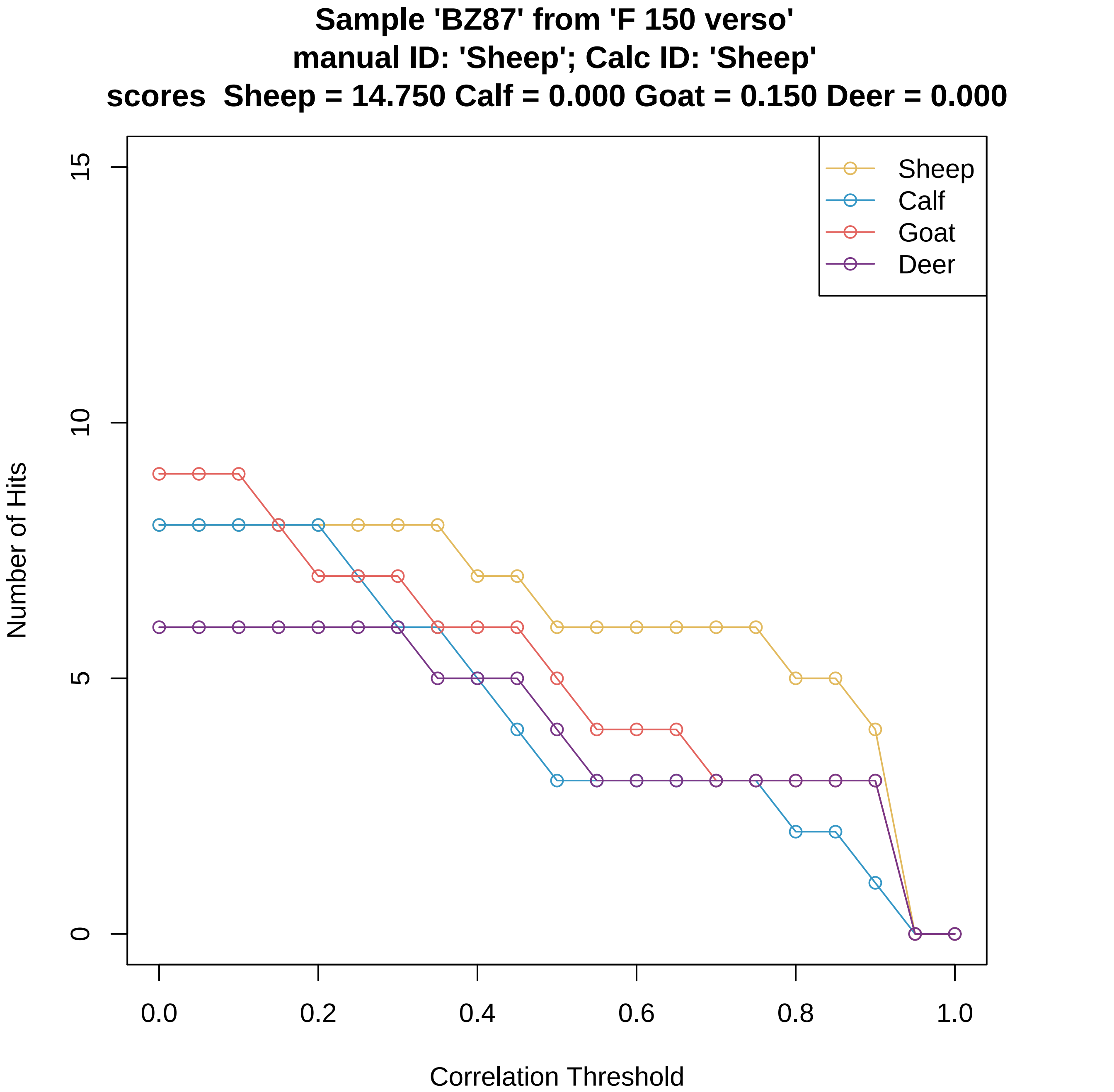
Sample 'BZ87' from 'F 150 verso'
manual ID: 'Sheep'; Calc ID: 'Sheep'
scores Sheep = 14.750 Calf = 0.000 Goat = 0.150 Deer = 0.000
Sheep
Calf
Goat
Deer
Number of Hits
0
5
10
15
0.0
0.2
0.4
0.6
0.8
1.0
Correlation Threshold

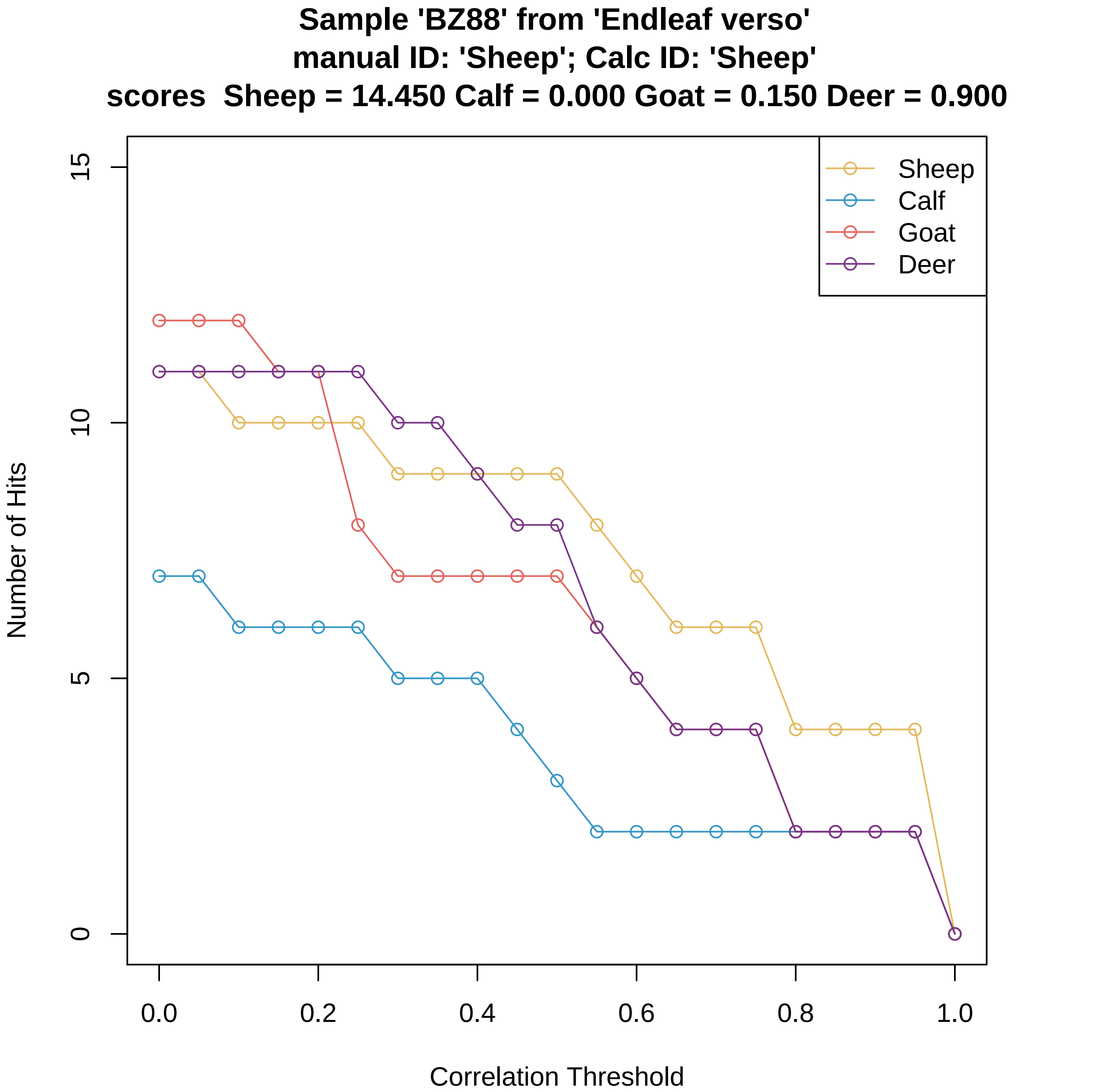

Sample 'BZ88' from 'Endleaf verso'
manual ID: 'Sheep'; Calc ID: 'Sheep'
scores Sheep = 14.450 Calf = 0.000 Goat = 0.150 Deer = 0.900
Sheep
Calf
Goat
Deer
Number of Hits
Correlation Threshold
0
5
10
15
0.0
0.2
0.4
0.6
0.8
1.0
